# Room-Temperature Cu(II) Radical-Triggered Alkyne C–H Activation

**DOI:** 10.1021/jacsau.1c00310

**Published:** 2021-10-06

**Authors:** Jack Devonport, Lauren Sully, Athanassios K. Boudalis, Storm Hassell-Hart, Matthew C. Leech, Kevin Lam, Alaa Abdul-Sada, Graham J. Tizzard, Simon J. Coles, John Spencer, Alfredo Vargas, George E. Kostakis

**Affiliations:** † Department of Chemistry, School of Life Sciences, University of Sussex, Brighton BN1 9QJ, U.K.; ‡ Institut de Chimie de Strasbourg (UMR 7177, CNRS-Unistra), Université de Strasbourg, 4 rue Blaise Pascal, CS 90032, F-67081 Strasbourg, France; § Université de Strasbourg, CNRS, Institut de Physique et Chimie des Matériaux de Strasbourg (IPCMS), UMR 7504, F-67000 Strasbourg, France; ∥ School of Science, Department of Pharmaceutical Chemical and Environmental Sciences, University of Greenwich, Central Avenue, Chatham Maritime ME4 4TB, U.K.; ⊥ UK National Crystallography Service, Chemistry, University of Southampton, Southampton SO1 71BJ, U.K.

**Keywords:** copper, ligand design, C−H activation, catalysis, radical, DFT, EPR, propargylamines

## Abstract

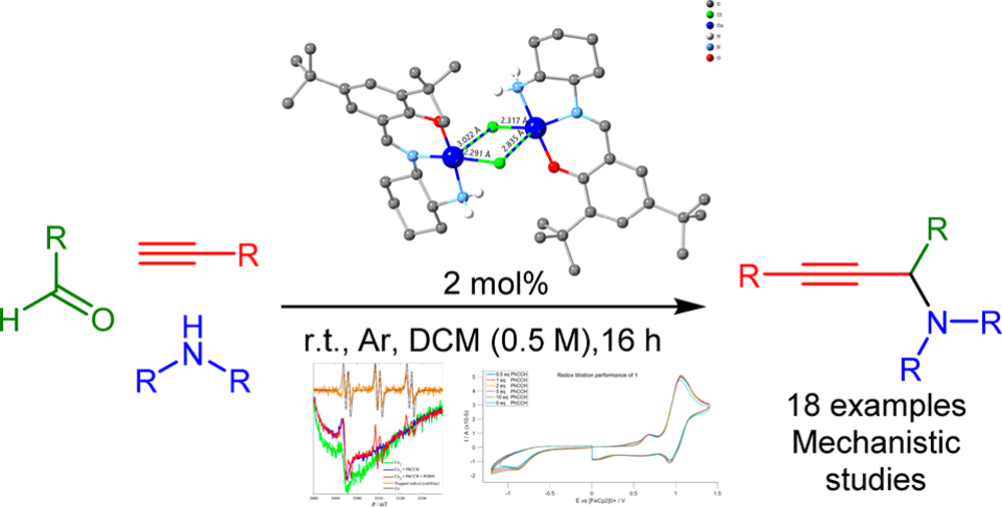

A dimeric Cu(II) complex [Cu(II)_2_L_2_(μ_2_-Cl)Cl] (**1**) built from an asymmetric tridentate ligand (2-(((2-aminocyclohexyl)imino)methyl)-4,6-di-*tert*-butylphenol) and weakly coordinating anions has been synthesized and structurally characterized. In dichloromethane solution, **1** exists in a monomeric [Cu(II)LCl] (**1′**) (85%)–dimeric (**1**) (15%) equilibrium, and cyclic voltammetry (CV) and electron paramagnetic resonance (EPR) studies indicate structural stability and redox retention. Addition of phenylacetylene to the CH_2_Cl_2_ solution populates **1′** and leads to the formation of a transient radical species. Theoretical studies support this notion and show that the radical initiates an alkyne C–H bond activation process via a four-membered ring (Cu(II)–O···H–C_alkyne_) intermediate. This unusual C–H activation method is applicable for the efficient synthesis of propargylamines, without additives, within 16 h, at low loadings and in noncoordinating solvents including late-stage functionalization of important bioactive molecules. Single-crystal X-ray diffraction studies, postcatalysis, confirmed the framework’s stability and showed that the metal center preserves its oxidation state. The scope and limitations of this unconventional protocol are discussed.

## Introduction

Copper catalysis is frequently used for molecular transformations of natural products, bioactive molecules, agrochemicals, and organic functional materials.^1−5^ Coupling reactions involving terminal alkynes in the construction of new C_sp_–X bonds (where X = C, O, or N)^3^ are vital in the design of important organic scaffolds. Several studies have investigated the mechanism of these transformations initiated by well-characterized or *in situ* generated catalytic species. These studies aimed to optimize catalyst performance either by fine-tuning the coordination environment of the catalyst or by varying coligands, solvents, and temperature. Copper is redox-plural (0/I/II/III), so the oxidation state in the starting component may differ from those in the reaction intermediates.^6,7^ However, particular attention has been given to Cu(I) derivatives due to the well-documented use of Cu(I) alkyne and alkynide complexes in substrate activation.^8−10^ Mixed-valent paradigms of well-characterized or *in situ* generated polynuclear catalytic species are known. For example, a mixed-valent Cu(I/II) complex was crystallographically characterized from a Glaser coupling,^11^ and dicopper Cu(I/I) and Cu(I/II) complexes were used in click chemistry.^12^ Furthermore, monitoring of in situ generated species suggests a Cu(II)/Cu(I) synergistic cooperation for alkyne C–H activation^13^ or direct observation of reduction of Cu(II) to Cu(I) by terminal alkynes.^14^ The general notion in organocopper chemistry is that the Cu(II)–carbon σ-bond is unstable and subject to spontaneous decomposition; however, it may be a key step in organic transformations.^4,5,15^ These ideas suggest that more examples are needed to fully understand the role of Cu(II) species in terminal alkyne activation processes. This is a challenging task since Cu(II)–aryl complexes are rare. Only recently, Cu(II) aryl complexes were found to promote C–C^16^ and C–O^17^ bond formation or have been proposed as vital intermediates in catalytic cycles.^18,19^

**Scheme 1:**
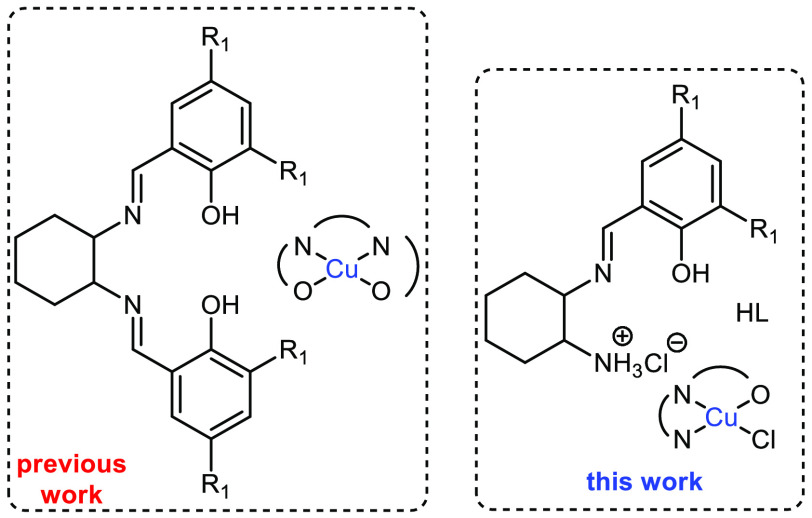
Well-Characterized Salen-Based Examples for Efficient A3 Coupling

The successful design of catalytically efficient Cu(I) and Cu(II) complexes requires the design of specifically tailored ligands to cater for each oxidation state. For a Cu(I) species, bi- or tridentate ligands are chosen and for Cu(II) complexes ligands with higher denticity are used. Consequently, a broader library of ligands can be used in the latter case, and targeted screening protocols can result in useful conclusions and optimized experimental conditions. Moreover, catalytic protocols involving well-characterized catalysts benefit from low loadings, reaction monitoring, and absence of additives. We have developed two different families of well-characterized Cu(II) components for alkyne C–H activation in the absence of additives,^20−24^ using a variety of polydentate ligands. The first series incorporated mono- or bidentate electron-deficient ligands.^20−22,24^ In this case, activation required elevated temperatures, but for the bidentate examples, activation at room temperature under N_2_ was observed. This unusual performance was rationalized by a structural rearrangement of the complex and in situ reduction of Cu(II) to Cu(I), possibly triggered by the alkyne.^14,24^ For the second series, inspired by pioneering research on galactose oxidase and the use of Cu(II)-radical based complexes,^25−27^ we showed that a reusable Cu(II) complex, built from a tetradentate N_2_O_2_ salen-based ligand, promoted alkyne C–H activation at room temperature, within 72 h, or 0.5 h at elevated temperature.^23^ Mechanistic and theoretical studies suggested a radical-based and single-electron transfer (SET) mechanism. Based on this evidence, we hypothesized that fine-tuning of the salen ligand and its respective Cu(II) complex would be key for an optimized C–H activation protocol. Inspired by recent results on an indium complex, built from a tridentate ligand, that was shown to facilitate a copolymerization reaction^28^ and the excellent performance of a similar tridentate ligand in an asymmetric Grignard synthesis of tertiary alcohols,^29^ we reasoned that the use of the tridentate monoprotic ligand HL (N_2_O)(Scheme 1) would provide a Cu(II) complex efficient for alkyne activation at room temperature. The ability to generate a phenoxide ion in the ligand would facilitate radical formation while a neutral amino group could temporarily accommodate the abstracted acetylenic proton.^23,30^ The reaction of HL with a Cu(II)X_2_ salt could yield the [Cu(II)LX] and/or [CuL_2_] species, and the latter would be likely to be catalytically inactive. Therefore, selecting the appropriate metal salt and metal/ligand ratio are crucial parameters in the design of the catalyst. In this work, we report the synthesis and characterization of a new Cu(II) complex [Cu(II)_2_L_2_(μ_2_-Cl)Cl] (**1**) and its ability to activate alkynes at room temperature. Cyclic voltammetry, EPR, single-crystal X-ray diffraction (SXRD), and theoretical studies corroborate a radical alkyne activation process via a four-membered ring (Cu–O···H–C_alkyne_).

## Results and Discussion

**Scheme 2:**
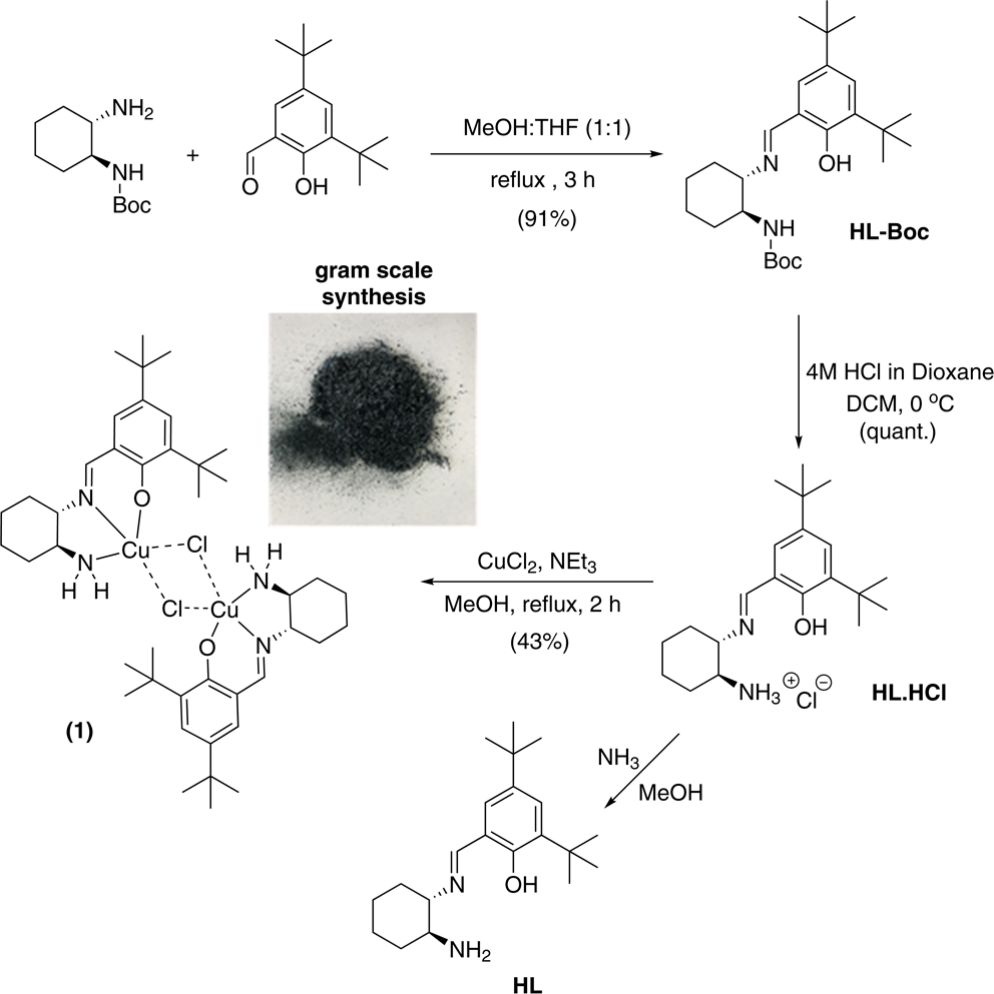
Synthesis of Dimeric Copper Catalyst [Cu(II)_2_L_2_(μ_2_-Cl)Cl] (**1**)

**Figure 1:**
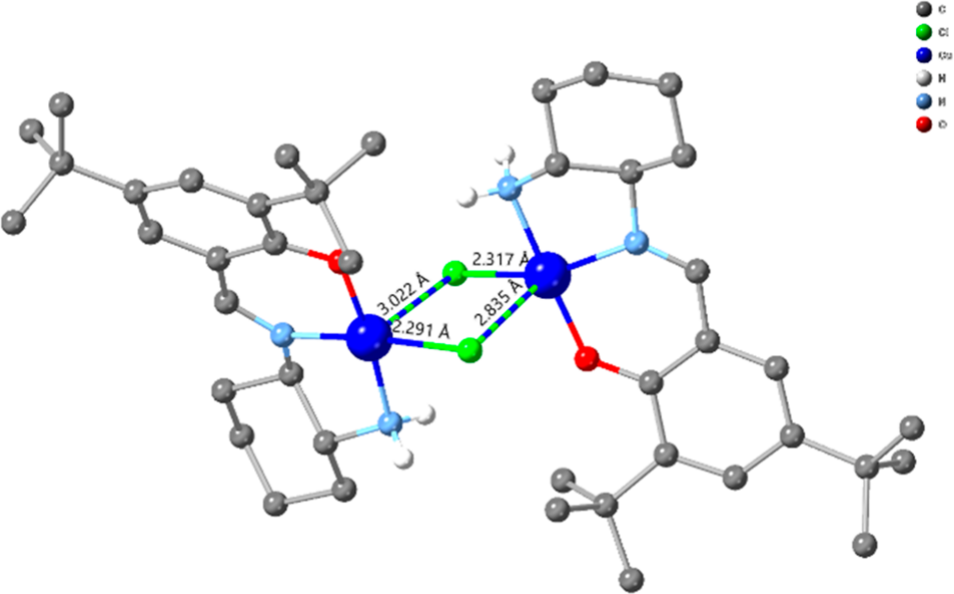
Crystal structure of **1**. All H atoms except NH_2_ are omitted for clarity. The elongated Cu–Cl bonds are shown with dashed lines.

*trans*-2-Aminocyclohexyl(imino)methyl)-4,6-di-*tert*-butylphenol hydrochloride, **HL·HCl**, was synthesized in two steps from the commercially available racemic *trans*-*N*-boc-1,2-cyclohexanediamine (Scheme 2). First, condensation with 3,5-di-*tert*-butylsalicylaldehyde affords the Boc-protected Schiff base, **HL-Boc**, which is then deprotected with HCl to afford **HL** as its HCl salt in quantitative yield. Due to the susceptibility of the **HL·HCl** ligand to hydrolyze to the corresponding amine and aldehyde as observed by ^1^H NMR, it was employed directly in the synthesis of the copper catalyst, **1**, without further purification. Attempts to synthesize the desired copper complex from the free base of **HL** failed due to the insolubility of the latter in suitable protic solvents. Given the monoprotic character of the ligand, we chose the weakly binding chloride anion to prevent the formation of the CuL_2_ species. The open-air reaction of **HL·HCl**, CuCl_2_, and Et_3_N, in MeOH, in a molar ratio 1:1:3, afforded compound **1** in 43% yield. The air-stable green crystalline material can be synthesized on a gram scale (Figure S6) and was characterized by SXRD (Figure 1), IR, UV–vis, electron paramagnetic resonance (EPR) spectroscopy, and cyclic voltammetry (CV) (see Figures S7–S14).

Compound **1**, in the solid-state, consists of an asymmetric dimer (Figure 1). The Cu(II) coordination shows two different elongated Cu–Cl bonds. The Cu(1)–Cl(2) bond length is 2.8345(10) Å, whereas the Cu(2)–Cl(1) bond length [3.0217(9)Å] is significantly longer. However, based on a literature survey^31−34^ and experimental data (see EPR discussion), we consider Cl(2) and Cl(1) as bridging and terminal chlorides, respectively. Geometrical calculations (see Table S1) show that the coordination geometry of Cu(1) is a hybrid of a square pyramidal or vacant octahedron (N_2_OCl_2_), while that of Cu(2) is square planar (N_2_OCl). The Cu–O, Cu–N, and Cu–Cl bond distances (Table S2) are typical for a Cu(II) compound, while bond valence calculations analysis (Table S3) suggests that both Cu centers are in oxidation state 2. The C–C and C=N and C–O bonds are typical for a salen-based ligand.^35−50^ The bridging chloride ion brings the two Cu(II) centers together with Cu···Cu 3.6639(6) Å, and the salen ligands complete the two coordination spheres.

**Figure 2:**
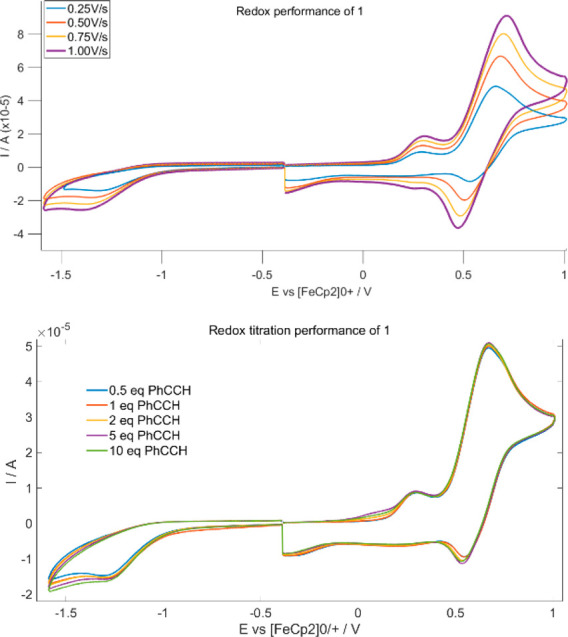
CV of compound **1** (upper) in the presence of phenylacetylene (lower).

Elemental (CHN) and thermogravimetric analysis (TG) confirmed macroscopic purity and thermal stability up to 202 °C respectively. Electron spray ionization mass spectrometry (ESI-MS) of **1** showed two peaks in the time-of-flight (TOF-MS-ES) positive-ion mode at *m*/*z* = 831.3768 and 392.1826, which, respectively, correspond to the dimeric disodium species [Cu_2_L_2_]Na_2_ and monomeric [CuL] species (Figure S8). The UV–vis spectrum in CH_2_Cl_2_ is typical for a CuN_2_OCl chromophore (Figure S10). To further understand the solution behavior of **1**, we performed CV studies in CH_2_Cl_2_ under a N_2_ atmosphere and with different current rates. The cyclic voltammograms of **1** display two well-separated one-electron reversible redox waves at *E*^1^_1/2_ = 0.30 V and *E*^2^_1/2_ = 0.71 V vs Fc^+^/Fc (Figure 2 upper);^35,46,51−55^ however, in the reductive region, a nonreversible process is feasible.

Based on literature evidence,^35,48,56,57^ the oxidation process shows a typical behavior for this kind of Cu(II) complexes compounds. It may correspond to either oxidation of a π-radical that would lead to the formation of an iminobenzoquinone from the iminobenzosemiquinone unit or oxidation of the Cu(II)-coordinated phenolate unit that would generate a Cu(II)–phenoxyl radical.^51^ Cyclic voltammetry studies of **HL** show that the ligand exhibits no reductive process (Figure S11). To confirm this hypothesis and before continuing to catalytic studies, we studied the compound in solution by EPR (Figures S13 and S14). Spectra from frozen CH_2_Cl_2_ solutions (0.77 mM for [Cu_2_]), collected at 100 K, revealed a principal component of axial, hyperfine-split spectra, characteristic of mononuclear Cu(II) complexes. Superposed on these were hyperfine-split, half-field transitions, indicating interacting species in solution. An additional feature at *g* = 1.85 supports this notion. Given the low concentration of the solution, these could stem from intramolecular interactions within dinuclear species, rather than intermolecular interactions between mononuclear species. This suggests that a fraction of the complex retains its dinuclear structure upon dissolution. The spectra were simulated considering a mixture of mononuclear (85%) and dinuclear (15%) species. For the dinuclear species, given the crystal structure, which reveals a superexchange pathway through the Cu(II) ions’ nonmagnetic orbitals, only dipolar interactions were considered (*J* = 0). For these, the relative orientations of the two *g*-tensors were explicitly taken into consideration, assuming two identical ions, whose *g*-tensors were collinear with their *A*-tensors. Assuming Cu(1) as a frame of reference with the *z*-axis coinciding with the d_*z*2_ orbital orientation, then the free variables were the angle of the Cu(2) concerning the *z*-axis, the intermolecular distance *r*. Attempts were made to simulate a tilt of the *g*_2_-tensor with respect to its local *y*-axis, but these did not afford significant improvements to the fits^58^ (Figures S12 and S13).

**Figure 3:**
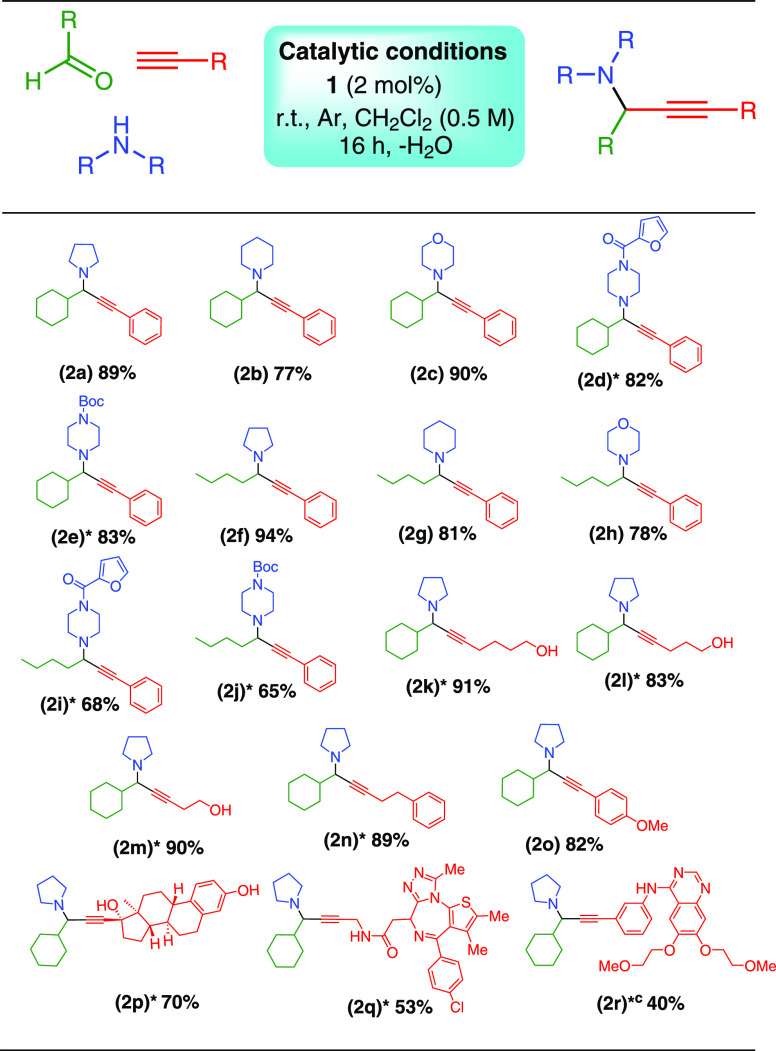
Substrate scope of the reaction with aldehydes, secondary amines, and alkynes.^a,b^ (a) Isolated yield, after purification. (b) Reaction conditions; catalyst (2 mol %), 1.0 mmol aldehyde, 1.1 mmol amine, 1.2 mmol alkyne, molecular sieves 4 Å, 16 h, Ar atmosphere, room temperature, solvent dichloromethane, concentration 0.5 M. (c) An extra 1 equiv of pyrrolidine was used to desalt the starting material.

Propargylamines^59,60^ are essential precursors in the synthesis of high value products, including isoindolines,^61^ oxazolidines,^62^ pyridones,^63,64^ and alkaloids.^65−67^ Following our work on Cu(II) protocols in A^3^ couplings,^23,24^ we selected cyclohexanecarboxaldehyde, pyrrolidine, and phenylacetylene as model substrates. Each catalytic reaction was performed in duplicate, and reproducibility and yields are given as an average with deviations of <10%. Screenings of concentration, time, and solvent are given in the Supporting Information (Table S5). Conversion to the desired product was complete within 16 h at room temperature under an Ar atmosphere and a significant improvement over our previous work (72 h). The present protocol is, to the best of our knowledge,^68,69^ an example of a Cu(II) A^3^-coupling catalyst that is efficient at room temperature over a relatively short time. The scope of the reaction is shown in Figure 3, applicable to aliphatic aldehydes/secondary amines and alkynes. Yields of the resulting propargylic amines were good to excellent. The reactions with aryl aldehydes or aliphatic amines at room temperature are unsuccessful; however, by increasing the temperature of the reaction (reflux) the corresponding products are obtained in good to moderate yields, but on this occasion a different catalytic pathway may be followed. Therefore, we decided not to include these data in the current article.

Purification was achieved using normal-phase chromatography with hexane and ethyl acetate as eluents with the compounds characterized by ^1^H and ^13^C NMR spectroscopy and HRMS. This method was used in the isolation of eleven previously unknown compounds (* in Figure 3), demonstrating the potential of this catalytic protocol in the synthesis of new organic compounds.

Ethinylestradiol is widely used as an estrogen-based oral contraceptive.^70^ Copper-catalyzed alkyne azide cycloaddition reactions have been used to develop triazole derivatives of ethinylestradiol. Related derivatives have cytotoxic activity against human cancer cell lines and can act as positron emission tomography (PET) imaging agents for estrogen receptor positive (ER^+^) breast cancer.^71,72^ To demonstrate the powerful scope of our catalyst in an A^3^ reaction, **1** was employed in the synthesis of a propargylamine steroid derivative, **2p**, which was isolated in good yield as a white solid. Other pharmaceutically relevant examples include A^3^ reactions on a (BRD4) bromodomain inhibitor JQ1 analogue,^73^ to afford **2q**, and on the clinically used epidermal growth factor receptor kinase inhibitor erlotinib, to afford **2r**. Therefore, we have shown that this chemistry can have impactful, late-stage applications relevant to the pharmaceutical industry, particularly as A^3^-couplings can introduce new sp^3^ chiral centers, with escape from flatland applications that are often employed to improve the physiochemical properties of compounds.^74^ Moreover, these reactions occur in the presence of other competing nucleophilic groups including alcohol, phenol, aniline, and amides.

## Mechanistic Understanding

**Figure 4:**
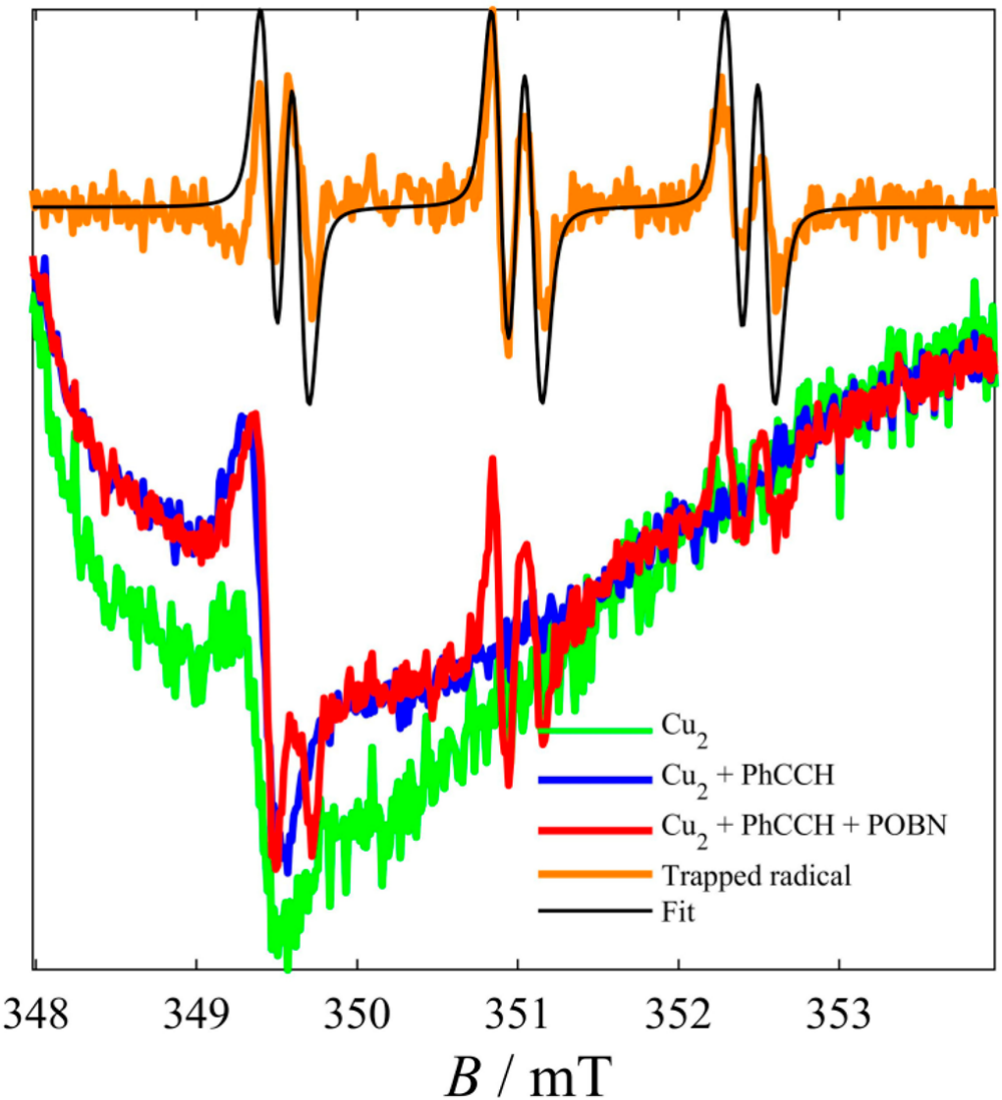
EPR spectra of fluid CH_2_Cl_2_ solutions (300 K) of **1** (green line), **1** + Ph–C≡C–H (10%) (blue line), and **1** + Ph–C≡C–H (10%) + POBN (10%) (red line). The trapped radical (orange line) is the difference between the red and blue spectra. Experimental conditions: *C* = 0.50 mM, *T* = 300 K, *f*_MW_ = 9.855 GHz; *P*_MW_ = 4.53 mW, *B*_mod_ = 0.5 G_pp_.

We designed in situ CV, IR, and EPR studies in a sense to mimic the catalytic reaction; the catalyst/alkyne ratio is 2:100. Our attempts to record the corresponding UV–vis or NMR data of highly concentrated and paramagnetic samples failed to provide conclusive results. Τitration CV studies on **1**, under an N_2_ atmosphere, with PhC≡C–H (0.5, 1, 2, 5, and 10 equiv) showed no significant effects on the oxidative process (Figure 2 lower) and only a slightly modified cathodic behavior, which suggests that during the PhC≡C–H addition complex **1** is stable. The metal center retains its oxidation state; thus, this one electron reduction process may correspond to reduction of the {**1**–PhCCH) complex. Following previous experiments,^23^ we monitored these titrations by EPR (Figure 4). Frozen and fluid solution EPR studies on pure **1** gave a hyperfine-split axial signal typical of a mononuclear Cu^II^ complex in a Jahn–Teller elongated ligand field. In addition, they revealed a minor component of hyperfine-split half-field transitions and a *g* = 1.85 absorption. The latter component was analyzed as derived from a dipole-coupled species of Cu^II^ ions (see the SI for fitting details). These results demonstrated that ∼85% of the complex decomposes into mononuclear species in solution, while a smaller fraction of dinuclear **1** persists. We performed PhC≡C–H loading experiments in the absence and presence of a spin trap α-(4-pyridyl *N*-oxide)-*N*-*tert*-butylnitrone (POBN). These show that alkyne loading further promotes the dimer’s dissociation into monomers. We conclude that complex **1**, upon dissolution, exists in an equilibrium of 85% (monomer)–15% (dimer); however, the alkyne loading drives the equilibrium to 100% (monomer) - 0% (dimer). In the absence of the spin trap, no additional signals were detected. In the presence of POBN, a characteristic signal was recorded which, after subtraction of the Cu(II) complex spectrum, was nicely fitted to a radical system coupled to a nitrogen and proton nuclei, with parameters *g* = 2.006, *A*_N_ = 41.2 MHz (14.71 G), *A*_1H_ = 5.96 MHz (2.13 G). These values are characteristic of POBN-radical species (Scheme S2).^75^ The typical values of this trapped radical do not allow further characterization, although, based on the prior art, formation of a Cu(II)- phenoxyl radical species seems likely.^54,76^ However, the key conclusion of this experiment is that the Cu center retains its oxidation state during this process.

**Figure 5:**
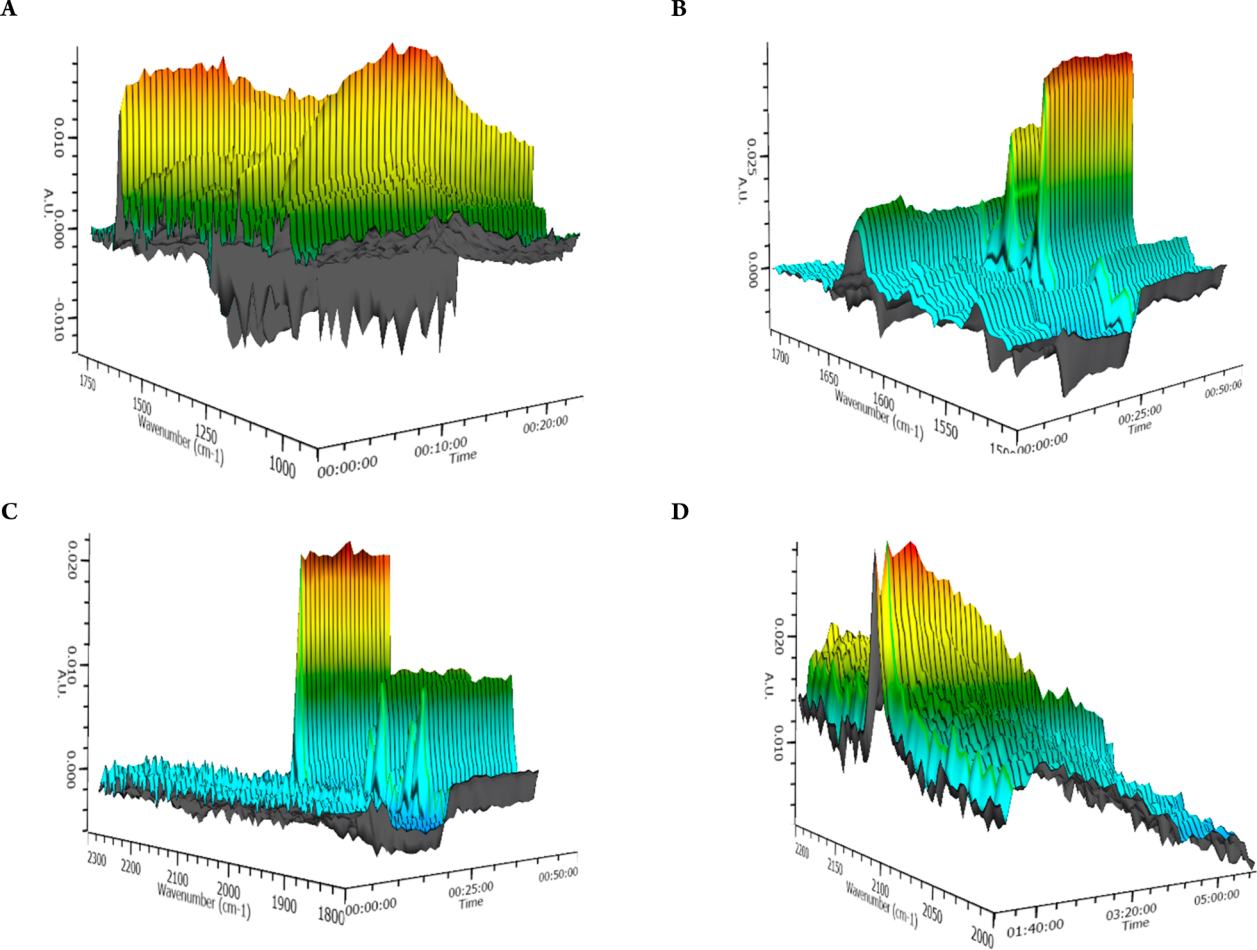
*In situ* IR studies of **1** (A), **1** and phenylacetylene (1:50 ratio) (B,C), and **1** and cyclohexanecarboxaldehyde, pyrrolidine, and phenylacetylene (1:50:50:50) (D).

To further understand the solution behavior of **1**, we performed time-dependent *in situ* IR studies at room temperature, under N_2_ atmosphere and CH_2_Cl_2_ solvent (Figure 5A). Monitoring of the dark green solution for 20 min identifies a structural change. The intensity of the peaks at 1627 and 1055 cm^–1^ indicative for the C=N and C–O bonds, respectively, fluctuates. This differentiation possibly can be attributed to the above-mentioned dimeric-monomeric equilibrium. Then, upon phenyacetylene addition in catalytic loading (1:50 ratio), the intensity of the peak at 1627 cm^–1^ decreases over time (Figure 5B) and possibly indicates the population of the monomeric species (**1′**), as noted in the EPR studies. At the same time, the intensity of the peak at 2112 cm^–1^, indicative of the C≡C stretching bond, decreases over time (Figure 5C), indicating interaction of the phenylacetylene moiety with **1′**. Finally, after the addition of the remaining substrates (cyclohexanecarboxaldehyde and pyrrolidine, Figure 5D), the intensity of the C≡C stretching bond peak declines over time, thus supporting the room temperature C–H activation process.

Given that the catalytic and CV studies were performed at room temperature and the EPR and SXRD studies were performed at 100 K, we considered that a variable-temperature SXRD study would shed light on structural features and behavior of compound **1**. SXRD data of compound **1** at three temperatures 200 K (**1**^**200**^), 298 K (**1**^**298**^), and 330 K (**1**^**330**^) showed only slight changes in the bond distances and angles (Tables S1 and S2, Figure S14) round the Cu centers. It is evident that compound **1**, at 330 K, exists as a monomer in the solid state, whereas the bond distances and angles of the organic platform, in all temperatures, are unaffected. The SXRD data at 298 K, at which the catalytic conversion takes place, indicate elongation of the Cu(1)–Cl(2) bond to 2.9562(14), (the Cu(2)–Cl(1) bond is 3.0806(14)), suggesting very weak interaction to form a dimeric species. From these studies, it is evident that in the solution phases at 298 K, compound **1** predominantly exists as a monomer (**1′**).

We performed control synthetic experiments to establish a reasonable mechanistic pathway. The open-air reaction (entry 2, Table 1) yielded **2a** in a moderate yield suggesting the need for an inert atmosphere. The different yields (entries 1 and 2, Table 1) may be due to catalyst structural changes, or a different mechanistic pathway. Both hypotheses could be rationalized if we take into account that similar complexes in the open air can be used as one-electron oxidized models of galactose oxidase^25−27^ and that the existence of Cu–O_2_–Cu species, that may inhibit alkyne binding, cannot be excluded.^77−80^ The EPR experiments (see above) confirmed a radical presence; therefore, we studied the reaction in the presence of a radical trap TEMPO (10%) under an Ar atmosphere (entry 3, Table 1), which led to **2a** in 16% yield. The significant difference in yields can be rationalized by consideration of a 2-fold TEMPO role, either to suppress the radical formation, as noted in our previous studies,^23^ or to oxidize the amine.^81^ The catalytic reaction with *in situ* blending of **HL** and CuCl_2_ gives **2a** in 26% yield (entry 4, Table 1). This result indicates that other noncatalytic Cu_*x*_L_*y*_Cl_*z*_ species may form, thus establishing the need for a precisely characterized species. However, to examine the ligand’s contribution to the catalytic activity, we experimented with **HL** (2 mol %) alone, without Cu (entry 5, Table 1), and observed no product formation. We then examined the efficacy of copper salts in different oxidation states (entries 6 and 7, Table 1) in 2% loadings, which yielded **2a** in 5% (using CuCl_2_) and 16% (using CuCl). In these experiments, the catalytic pathway may be a different, and the reduction of Cu(II) to Cu(I) by terminal alkynes is feasible.^13,14^ Next, we attempted to expand the scope of the reaction to primary amines; however, the reaction with aniline (entry 8, Table 1) failed to yield the desired product.

**Table 1:**
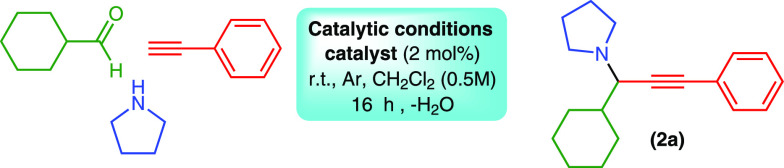
Various Experiments to Elucidate the Mechanism

entry	atmosphere	additives	yield of **2a**^a^^,^^b^ (%)
1	Ar		90
2	open air		56
3	Ar	TEMPO (10 mol %)	16
4	Ar		26^c^
5	Ar		0^d^
6	Ar		5^e^
7	Ar		16^f^
8	Ar		NR^g^
9	Ar		16^h^
10	open Air/N_2_		43^23^^,^^i^

a Isolated yields.

b Reaction conditions, catalyst (2 mol %), 1.0 mmol of aldehyde, 1.1 mmol of amine, 1.2 mmol of alkyne, molecular sieves 4 Å, solvent; dichloromethane 2 mL, concentration 0.5 M, room temperature, Ar atmosphere, 16 h.

c In situ formation of the catalyst HL/CuCl_2_.

d Reaction with **HL** (2 mol %).

e Reaction with CuCl_2_ (2 mol %) in place of **1**.

f Reaction with CuCl (2 mol %) in place of **1**.

g Reaction with aniline in place of pyrrolidine.

h Reaction with recovered material **3** (2 mol %) (vide supra), conversion by ^1^H NMR.

i Reaction completed in 24 h.

**Figure 6:**
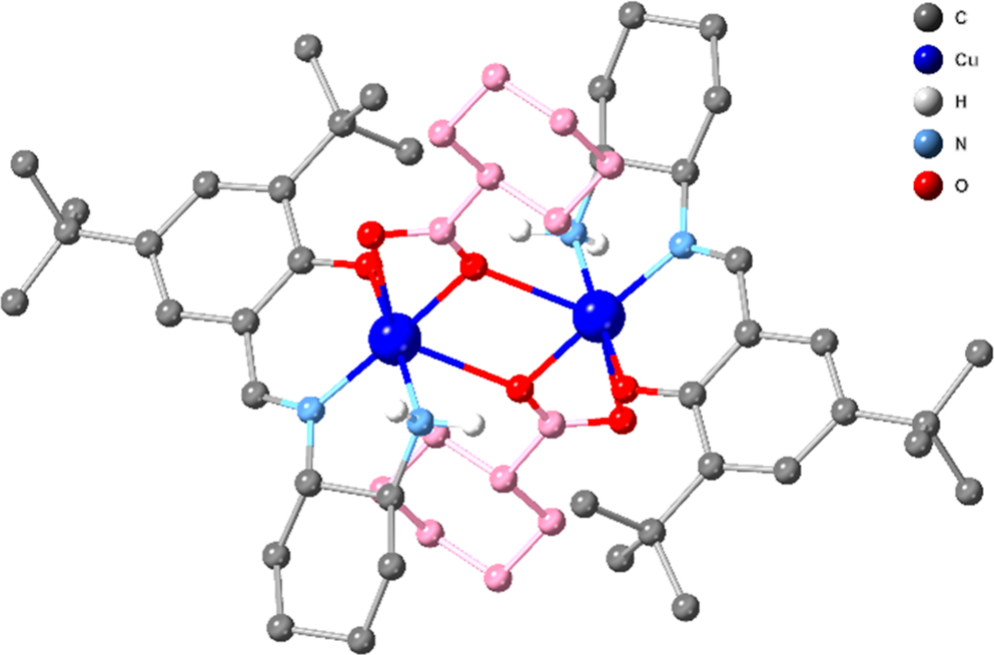
Crystal structure of the recovered material **3**. All H-atoms except NH_2_ are omitted, and C atoms of carboxylate unit are drawn in light purple for clarity.

Last, we attempted to recrystallize a green solid obtained from all the control experiments, post catalysis, with dichloromethane. From the reactions in open air (entry 2, Table 1), small green crystals were obtained, and their crystal structure was determined. The SXRD data at 120 K reveal the existence of a different dimeric complex formulated as [Cu(II)_2_L_2_(C_6_H_10_CO_2_)_2_] (**3**,Figure 6). Compound **3** is built from the starting organic ligand and cyclohexanecarboxylate bridging anions. We assume the carboxylate formation is the result of the oxidation of unreacted cyclohexanecarboxaldehyde during the workup process. For the reactions performed under an O_2_ atmosphere, compound **3** is recovered almost quantitatively. Interestingly, the use of **3** in 2% loading as catalyst in the parent reaction yielded **2a** in 16% (conversion), indicating that the presence of strongly binding anions inhibits the catalytic activity which may be the cause of lower yields observed in open air (entry 9, Table 1). Lastly, in none of the reactions was the redox A^3^-product observed in the crude ^1^H NMR.^82^

**Scheme 3:**
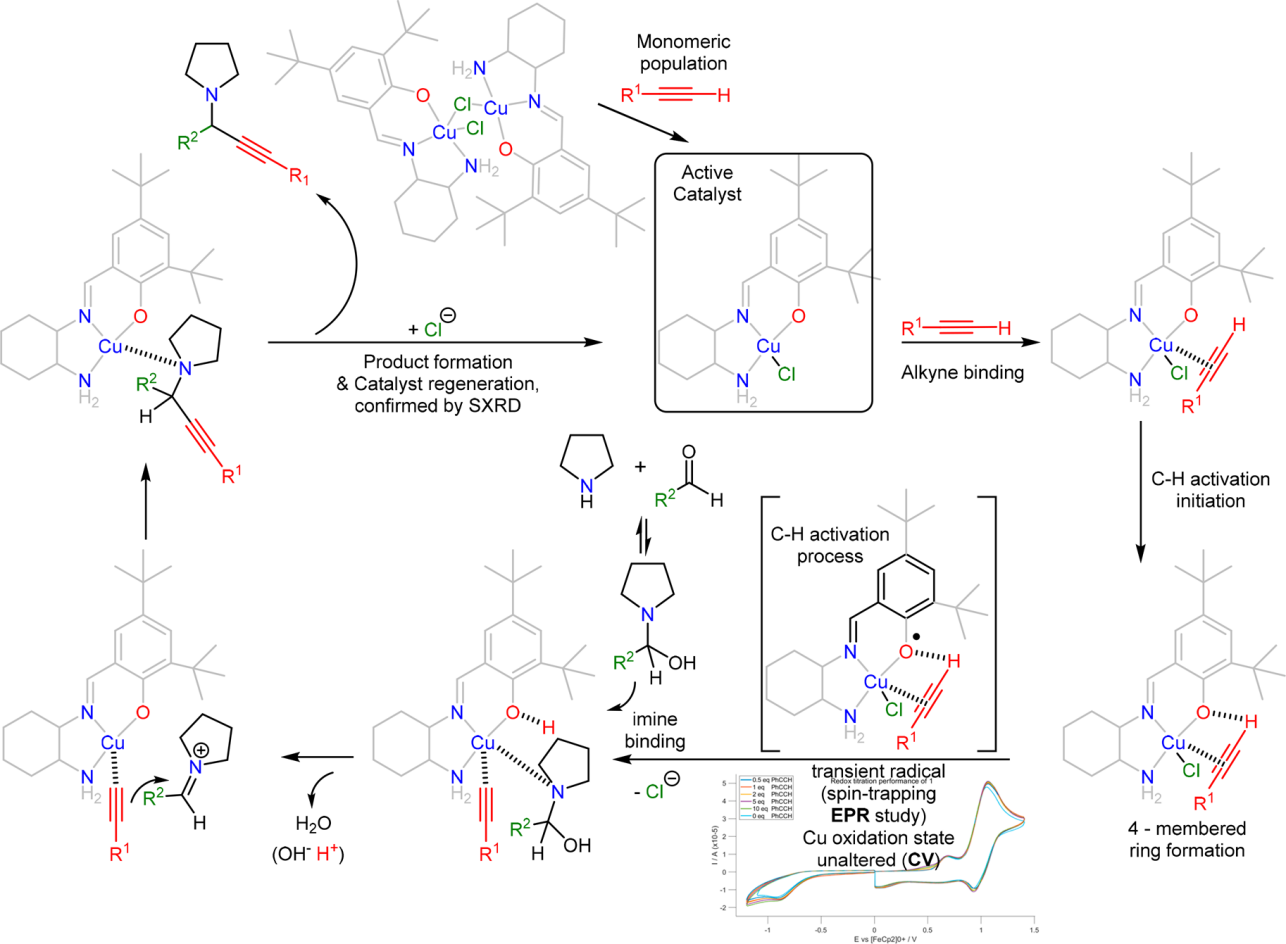
Proposed Mechanism for the Cu(II)-Promoted A^3^ Coupling The transient radical is formed during C–H activation not the one directly observed by EPR; instead, we observe its spin-trapped adduct formed by reaction with POBN (Scheme S2).

Taking all the above into account and considering the following observations, (i) the absence of side products (heterocoupling, bis-adduct) or metallic Cu precipitate in the crude NMRs, (ii) the EPR experiments, discounting the possibility of two monomers activating the Ph–C≡C–H entity, or the existence of a dimeric species, (iii) that the coordinating metal labilizes the terminal hydrogen, allowing a weak base (Cl^–^ anion or N/O heteroatoms of the ligand) to deprotonate the alkyne, thus forming the activated acetylide as has been confirmed by IR and NMR studies,^83,84^ (iv) the fact that the substituted phenolate and primary amines have similar p*K*_b_ values,^85−88^ and (v) that the activated acetylide subsequently couples with the corresponding imine,^89−91^ we propose the following mechanism (Scheme 3). Compound **1** in solution exists as an 85–15% monomer–dimer equilibrium, and the excess of Ph–C≡C–H populates **1′** (step 1). Then the alkyne binds to the monomer (step 2) and initiates activation (step 3). This procedure follows a 4-membered ring formation, which subsequently triggers the radical formation to complete the activation process while the imine moiety replaces the Cl^–^ anion (step 4). Then, the acetylide couples with the *in situ* generated iminium species, which is formed via H^+^-mediated elimination of water^90^ (step 5) to form the propargylamine product (step 6). The final step involves catalyst regeneration and product release (step 7). The proposed mechanism resembles the one proposed by Knochel et al.;^89^ however, the major difference is that in the present case Cu in the precatalyst is in oxidation state II.

## Theoretical Calculations

**Figure 7:**
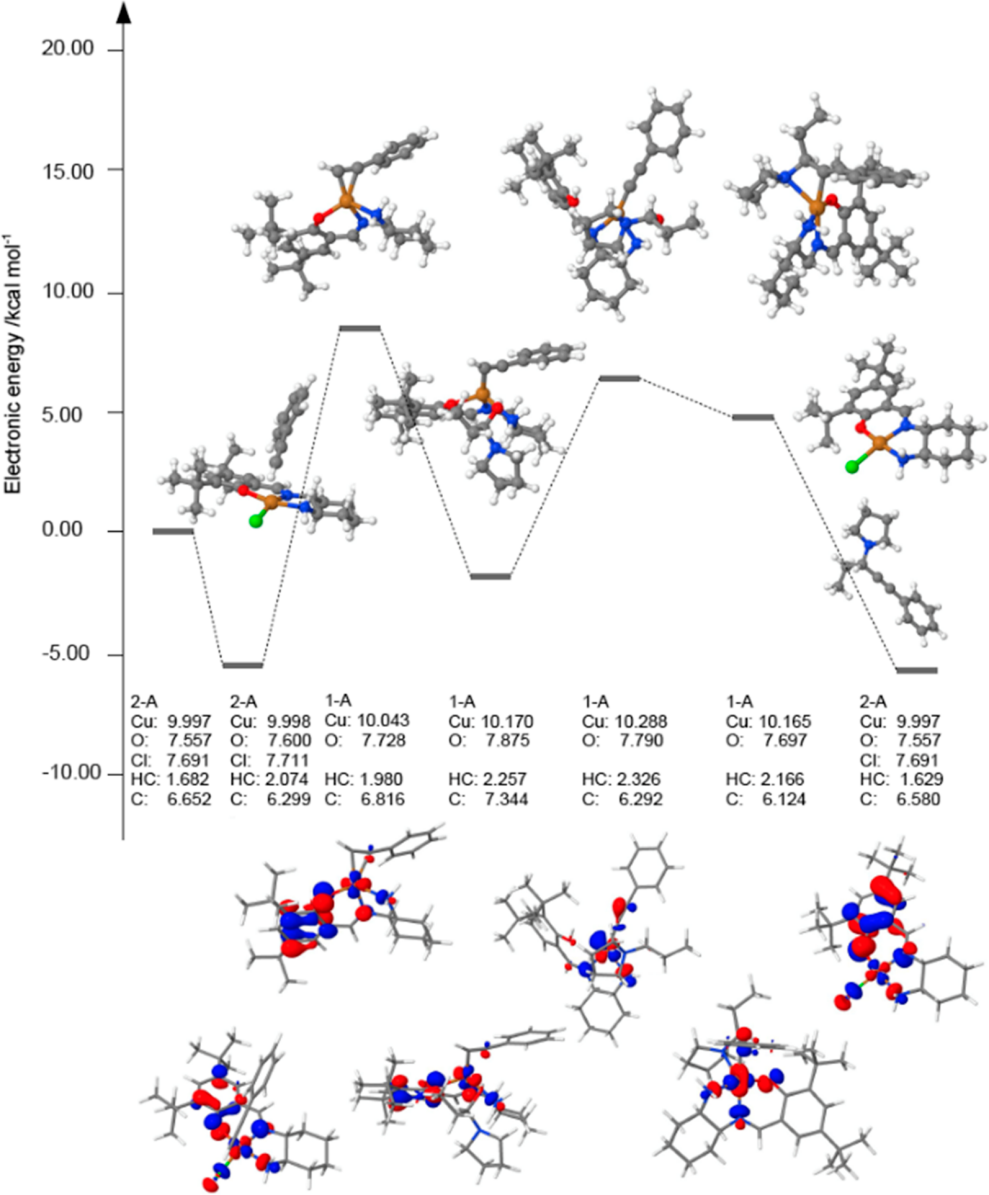
Proposed reaction profile for the Cu(II)-promoted A^3^ coupling: total electronic energies, electronic state, highest occupied molecular orbitals, and Bader charge analysis.

Given the peculiar catalytic process observed, we further carried out theoretical calculations within Kohn–Sham density functional theory (DFT) at the OLYP/def2-SVP D3 level with PCM (dichloromethane) implicit solvation. Figure 7 showcases a qualitative reaction profile and a description of the electronic structure of key species. As mentioned earlier in the manuscript, initial activation of the alkyne by the monomeric neutral doublet 1 takes place by way of through-space interaction and surprisingly brings the resulting van der Waals (v-d-W) complex to about 5 kcal/mol more stable. We tentatively ascribe this to dispersion interactions and the relaxation toward quasi-tetrahedral symmetry of the copper center. Full activation through dative alkyne capture with concomitant leaving of neutral chlorine occurs, bringing the system to some 8 kcal/mol more energetic. Calculations highly suggest that the now nonhalogenated system needs to switch its state from doublet ^2^A to singlet ^1^A. Combining this with the fact that (a) to be consistent in terms of full electronic energy, the departing chlorine has to be treated as nonsolvated species, (b) upon activation as mentioned in the previous step, the Cl–Cu bond distance increases, and (c) the number of electrons increases around the oxygen adjacent to the metal, we conclude that there is homolytic-like Cu–Cl bond breaking, and subsequently, a nondirectional interaction between the Cl and the catalytic adduct takes place. To further support this, we optimized the activation structure with Br in lieu of Cl; the Cu–N bonds are not affected; however, the distance between the two radical centers, the Cu and the adjacent O, is dependent on the nature of the halogen, with Cu–O slightly longer in the chlorine species. Based on the analysis of charge displacement upon activation, it can be suggested that the alkyne induces electron movement toward the oxygen and the halogen centers with the concomitant electrophilic attack of the copper center on the alkyne, acquiring in the process a decrease in its oxidation state. In the next stage, akin to the first step, another through-space interaction brings the system to lower energy by 10 kcal/mol, this time by v-d-W capture of NR_2_. One should note that conformational change occurs, contributing to the observed shift in energy. Full head-on interaction between the alkynyl carbon and the metal center follows with the liberated hydrogen scavenged by the oxygen, putting the system to 8 kcal/mol higher Fusion of the enamine and the alkyne with isochronous water production sets the system again to about 2 kcal/mol more stable. One can note that charge movement involves solely the alkyne carbons needed to form the C–C bond. The additional role of the metal is evident at this stage, that is, to template the resulting N–C–C catenate. Lastly, a downward release of the product is associated with 10 kcal/mol, with the accompanying reformation of the Cu–Cl bond hence recycling the catalyst. Further interesting points need to be mentioned. First, the copper center switches from Cu(II) to Cu(I) form transiently, as evidenced by the Bader charge analysis.^92−94^ Indeed, upon change of state, one notes an increase in the metallic valence electron index; in the current formalism, this is enough to indicate a change of oxidation state.^95,96^ The change would not have taken place if the center had not been Cu(II). Second, one could ascribe the facile capture of the reactants to the charge dynamics lability provided by the ligands as explained above; indeed, a closer inspection of the SOMOs and HOMOs indicate the permanent involvement of the metallic *d* manifolds and, surprisingly, both the ring systems of the ligand and the chlorine. Given the complexity of the reaction, notably the involvement of noncovalent interactions and change of spin-state, the inclusion of both electronic and vibrational entropy contributions in terms of calculations would be biased. Hence, we report the reaction profile in terms of the total electronic energies only, which in our opinion, fits our purpose and the scope of the manuscript. Lastly, both the isotropic Fermi contact couplings and the eigenvalues from the diagonalization of the spin dipole couplings tensor (data not shown) indicate some unpaired electron–nuclear coupling in the oxygen, further validating the experimental results.

## Conclusions

We have synthesized and characterized a Cu(II) complex that can activate alkynes at room temperature without base or additives with very low loadings in only 16 h. To the best of our knowledge, the present unconventional protocol is the first example of an A^3^ coupling incorporating such a time efficient Cu(II) component. Vital to this success is the use of (i) the asymmetric tridentate ligand, which matches the coordination geometry of the metal center, (ii) weakly binding anions that prevent the formation of other, possible catalytically inactive, species, (iii) a phenoxido moiety that triggers the radical formation, and (iv) unsaturated heteroatoms on the organic framework that accommodate the acetylenic proton during the activation process. Thus, the appropriate ligand design allowed us to achieve an exceptional Cu(II) alkyne activation process and develop an environmental friendly catalytic protocol applicable for synthesizing propargylamines in high yields. EPR, CV, IR, and DFT studies shed light on this peculiar C–H activation process and our future efforts will focus on catalyst development, notably chiral versions, and their applicability to other organic transformations and asymmetric syntheses relevant to the late-stage elaboration of pharmaceutically valuable scaffolds.

## Supporting Information Available

X-ray data for compounds **1** and **6** (CIF)
TG, UV–vis, IR, EPR, and CV data, Cartesian coordinates, copies of ^1^H and ^13^C NMR and HRMS of organic compounds (PDF)

## Supplementary Material

au1c00310_si_001.cif

## Notes

All data supporting this study is provided as supplementary information accompanying this paper. Crystal structures are available from the Cambridge Crystallographic Data Centre at [https://www.ccdc.cam.ac.uk/structures/].
