# Supplementary material for: Room-Temperature Cu(II) Radical-Triggered Alkyne C–H Activation
Source: JACS Au. 2021 Oct 6;1(11):1937–48. doi: 10.1021/jacsau.1c00310 (PMC8611675; doi:10.1021/jacsau.1c00310)
Supplement: Supplementary file 2 — au1c00310_si_002.pdf [file au1c00310_si_002.pdf]

Supporting Information

Room temperature Cu(II) radical-triggered alkyne C-H activation

Jack Devonport,[a] Lauren Sully,[a] Athanassios K. Boudalis,[b,c] Storm Hassell-Hart,[a] Matthew C. Leech,[d] Kevin Lam,[d] Alaa Abdul-Sada,[a] Graham J. Tizzard,[e] Simon J. Coles,[e] John Spencer,[a]* Alfredo Vargas[a]* and George E. Kostakis[a]*

[a]Department of Chemistry, School of Life Sciences, University of Sussex, Brighton BN1 9QJ, UK. E-mail:
[b]Institut de Chimie de Strasbourg (UMR 7177, CNRS-Unistra), Université de Strasbourg, 4 rue Blaise Pascal, CS 90032, F-67081 Strasbourg, France.
[c]Université de Strasbourg, CNRS, Institut de Physique et Chimie des Matériaux de Strasbourg (IPCMS), UMR 7504, F-67000 Strasbourg, France.
[d]School of Science, Department of Pharmaceutical Chemical and Environmental Sciences, University of Greenwich, Central Avenue, Chatham Maritime, ME4 4TB, UK.
[e]UK National Crystallography Service, Chemistry, University of Southampton, SO1 71BJ, UK.
* G.Kostakis@sussex.ac.uk , * Alfredo.Vargas@sussex.ac.uk, * j.spencer@sussex.ac.uk,

Table of Contents

Materials. 2
Instrumentation. 2
EPR. 2
Catalysts synthesis and characterization. 3
Characterization of Ligands 5
Characterization of Catalyst 8
EPR spectra of 1 11
X-Ray crystallography 13
Geometrical calculations 14
Screening of (1) for the synthesis of propargylamines. 17
Theoretical calculations 19
Synthesis and Characterization data for propargylamines 31
Characterisation 38
References 74

Materials.

All reagents were purchased from Sigma Aldrich, Fluorochem, Tokyo Chemical Industry, Apollo Scientific, Fischer Scientific or Alfa Aesar and used without further purification. Experiments were performed under aerobic conditions or argon.

Instrumentation.

NMR spectra were recorded with a Varian VNMRS 600 at 25 °C, at either 600 MHz or 151 MHz in Chloroform-d or DMSO-d_6. Chemical shifts are quoted in parts per million (ppm). Coupling constants (J) are recorded in units of Hz. FT-IR spectra were recorded over the range of 4000–650 cm^{-1} on a PerkinElmer Spectrum One FT-IR spectrometer fitted with a UATR polarisation accessory. HRMS data were obtained with a Bruker Daltonics Fourier Transform (FTMS) Apex II spectrometer with electrospray ionization (ESI) and methanol as solvent. HR-MS data were obtained on a VG Autospec Fissions instrument (EI at 70 eV). Molecular ions are reported as mass/charge (m/z) ratios. Thermogravimetric analysis was carried out with a Thermogravimetric analyzer Q-50 V20.13 using a platinum pan, in a nitrogen atmosphere from 25 – 800 °C, at a scan rate of 5 °C/min. UV−Vis measurements (280-750 nm) were performed at room temperature (15-20˚C) using a Thermo Scientific Evolution 300 UV-Vis spectrophotometer equipped with 5mm path length quartz cells, and the collected data were processed using the Vision Pro software. Purification of compounds with normal-phase silica flash column chromatography was conducted on a Teledyne Isco Combiflash with UV detection at all wavelengths. Elemental analysis was carried out at the London Metropolitan University.

Electrochemical measurements were carried out using an Autolab 302N potentiostat interfaced through Nova 2.0 software to a personal computer. Electrochemical measurements were performed in a glovebox under oxygen levels of less than 5 ppm using solvent that had been purified by passing through an alumina-based purification system. Diamond-polished glassy carbon electrodes of 3 mm diameter were employed for cyclic voltammetry (CV) scans. CV data were evaluated using standard diagnostic criteria for diffusion control and chemical and electrochemical reversibility. The experimental reference electrode was a silver wire coated with anodically deposited silver chloride. The electrochemical potentials in this article are referenced to the ferrocene/ferrocenium couple. Its addition to the analyte solution obtained the ferrocene potential.

EPR.

X-band EPR spectra were collected on an EMXplus spectrometer fitted with an EMX microX bridge, using a high-sensitivity Bruker ER4122SHQE cavity operating in the TE_{011} mode.

For low-temperature experiments (9.31 GHz), the cavity was fitted with an ESR900 dynamic continuous flow cryostat controlled with an Oxford ITC503S Intelligent Temperature Controller. All samples were deoxygenated to remove eventual interactions with paramagnetic O_2 molecules.

Room-temperature spectra (9.85 GHz) were collected from fluid solutions in 100 µL glass capillaries (Hirschmann) sealed from both ends with sealing wax. Solutions (0.5 mM with respect to Cu(salen) monomer) were prepared inside the glovebox, with CH_2Cl_2 distilled from an MBraun solvent purification system, collected inside the glovebox through a dedicated transfer line and stored over molecular sieves. Phenylacetylene was also stored inside the glovebox over molecular sieves.

For the catalytic reaction, complex (**1**) (0.83 mg, 0.00097 mmol) was mixed with POBN (1.13 mg, 0.00058) in CH_2Cl_2 (3.88 mL) and then the mixture was added to phenyl acetylene (4.0 mg, 0.039 mmol). Upon mixing with PhCCH, no appreciable color change took place. For reference, the spectra of Cu-salen+PhCCH, POBN and Cu-salen solutions of approximately similar concentrations were recorded.

Spectra were fitted with *Easyspin* v6.0 *pepper* (for frozen solution) and *garlic* (for fluid solution) functions, using custom-made routines.[1]

Catalysts synthesis and characterization.

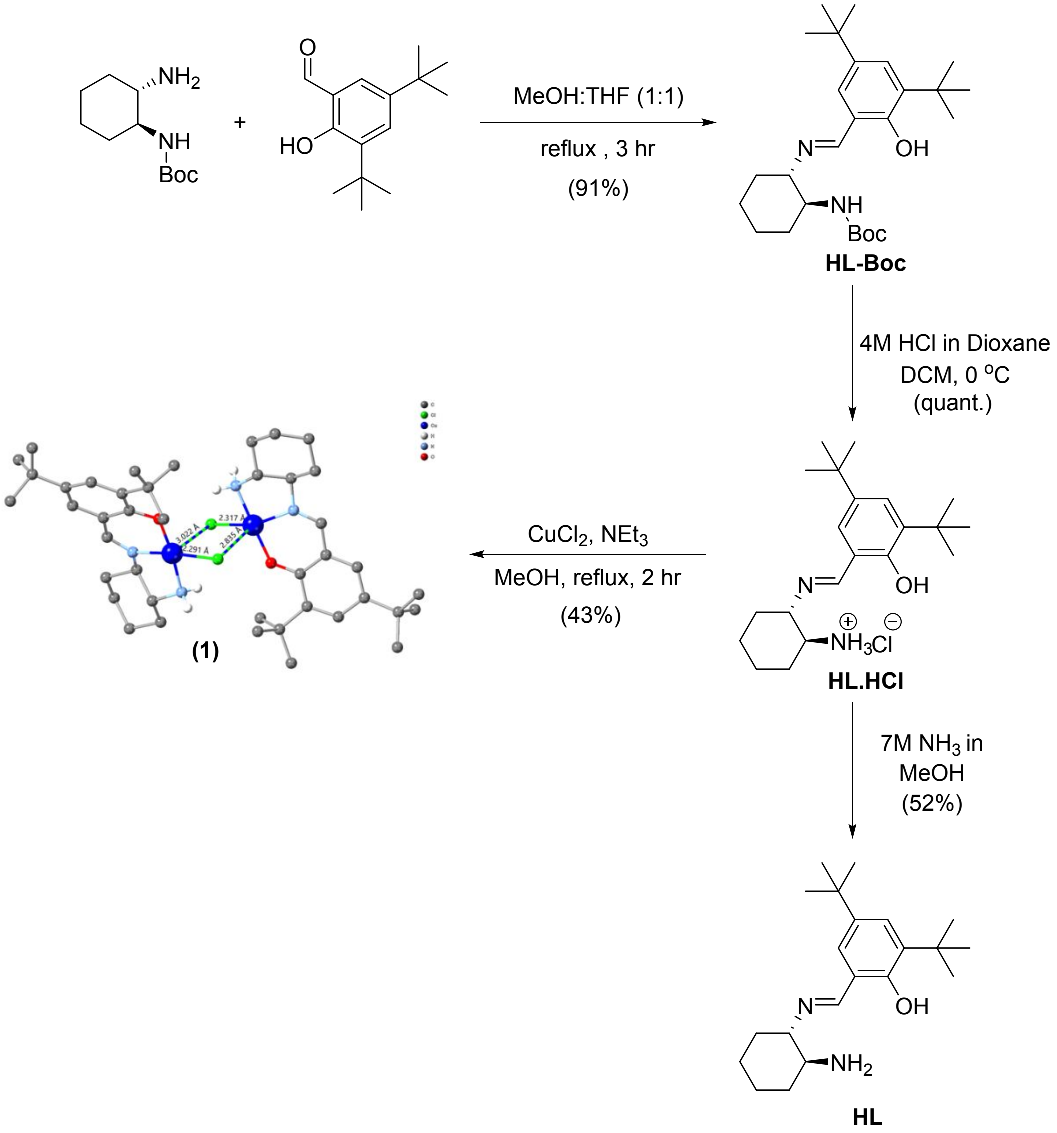

Scheme S1: synthetic route toward catalyst **(1)**

Synthetic protocols:

tert-Butyl 2-((*E*)-(3,5-di-*tert*-butyl-2-hydroxybenzylidene)amino)cyclohexyl)carbamate **(HL-Boc)**

trans-N-Boc-1,2-cyclohexanediamine (3.00 g, 14 mmol) and 3,5-di-*tert*-butylsalicylaldehyde (3.30 g, 14 mmol) were combined in MeOH:THF (1:1, 200 mL). The reaction mixture was heated at reflux for 3 hours, cooled to R.T and the excess solvent was removed *in vacuo.* The crude material was washed with cold EtOH (ca. 100 mL) and dried over suction to afford **HL-Boc**. Yellow solid (5.45 g, 12.7 mmol, 91%); ^{1}H NMR (600 MHz, dmso) δ 13.80 (s, 1H), 8.42 (s, 1H), 7.26 (s, 1H), 7.19 (s, 1H), 6.73 (d, *J* = 9.3 Hz, 1H), 3.45 – 3.38 (m, 1H), 3.06 – 2.99 (m, 1H), 1.87 – 1.78 (m, 2H), 1.75 – 1.68 (m, 2H), 1.60 – 1.50 (m, 1H), 1.35 (s, 9H), 1.26 (s, 12H), 1.14 (s, 9H); ^{13}C NMR (151 MHz, dmso) δ 165.1, 161.1, 155.4, 132.5, 131.9, 119.1, 118.7, 116.8, 77.7, 71.9, 53.9, 33.6, 32.0, 28.4, 25.1, 24.3; (HRMS + pTOF-ES) cald $C_{26}H_{43}N_2O_3$ $[M + H]^+$: 431.3274, observed: 431.3275.

trans-2-Aminocyclohexyl(imino)methyl)-4,6-di-tert-butylphenol hydrochloride **(HL.HCl)**

A solution of compound **(HL-Boc)** (4.00 g, 9.3 mmol) in dichloromethane (60 mL) was cooled to 0 ^{o}C. 4M HCl in dioxane (20 mL) was added slowly and the reaction was stirred at 0 ^{o}C until all the starting material was consumed as determined by TLC (Hex:EtOAc, 1:1). Hexane was added to the solution until a yellow precipitate formed (ca. 200 mL). The yellow product was collected via suction filtration and used in the next step without any further purification. Yellow solid (4.05 g, 11 mmol, assumed to be quantitative).

trans-2-Aminocyclohexyl(imino)methyl)-4,6-di-tert-butylphenol **(HL)**

HL.HCl (1g, 2.73 mmol) was dissolved in 7M ammonia in MeOH (10 ml, excess) and stirred at reflux for 1 hour. The reaction mixture was cooled to room temperature and concentrated. The concentrated material was suspended in dichloromethane (50 mL) and washed with deionised water (2 x 30 mL) and sat. aq. $NaHCO_3$ (30 mL). The organic phase was dried over $MgSO_4$ and concentrated to afford **HL**. Yellow solid (468 mg, 1.42 g, 52%); ^{1}H NMR (600 MHz, dmso) δ 13.9 (s, 1H), 8.5 (s, 1H), 7.2 (s, 1H), 7.1 (s, 1H), 1.9 (d, *J* = 12.6 Hz, 1H), 1.8 (d, *J* = 8.1 Hz, 1H), 1.7 – 1.6 (m, 1H), 1.5 (t, *J* = 10.2 Hz, 1H), 1.3 (s, 13H), 1.2 (s, 12H); ^{13}C NMR (151 MHz, CDCl3) δ 165.8, 158.0, 139.8, 136.3, 126.7, 126.0, 117.8, 72.4, 34.9, 34.0, 33.3, 31.4, 29.4, 24.3; (HRMS + pTOF-ES) cald $C_{21}H_{35}N_2O$ $[M + H]^+$: 331.2749, observed: 331.3756

Complex **1**, was synthesized according to the following procedure. **HL.HCl** (4.04 g, 11 mmol) and triethylamine (4.60 mL, 33 mmol) were combined in MeOH (100 mL) and stirred at RT. After 10 minutes, anhydrous $CuCl_2$ (1.52 g, 11 mmol) was added, turning the colour of the solution to dark green. The reaction mixture was heated at reflux for 2 hours before being cooled to RT and concentrated *in vacuo*. The crude product was then recrystallized from hot MeCN to give **1** as a dark green crystalline solid (2.01 g, 2.35 mmol, 43%); FTIR ν_{max} / cm^{-1}: 3339, 2948, 2862, 1622, 1434, 1167. Elemental analysis (%) calcd (found) for $C_{42}H_{66}Cl_2Cu_2N_4O_2$: C, 58.86 (58.28); H, 7.76(7.54); N, 6.54 (6.34).

Characterization of Ligands

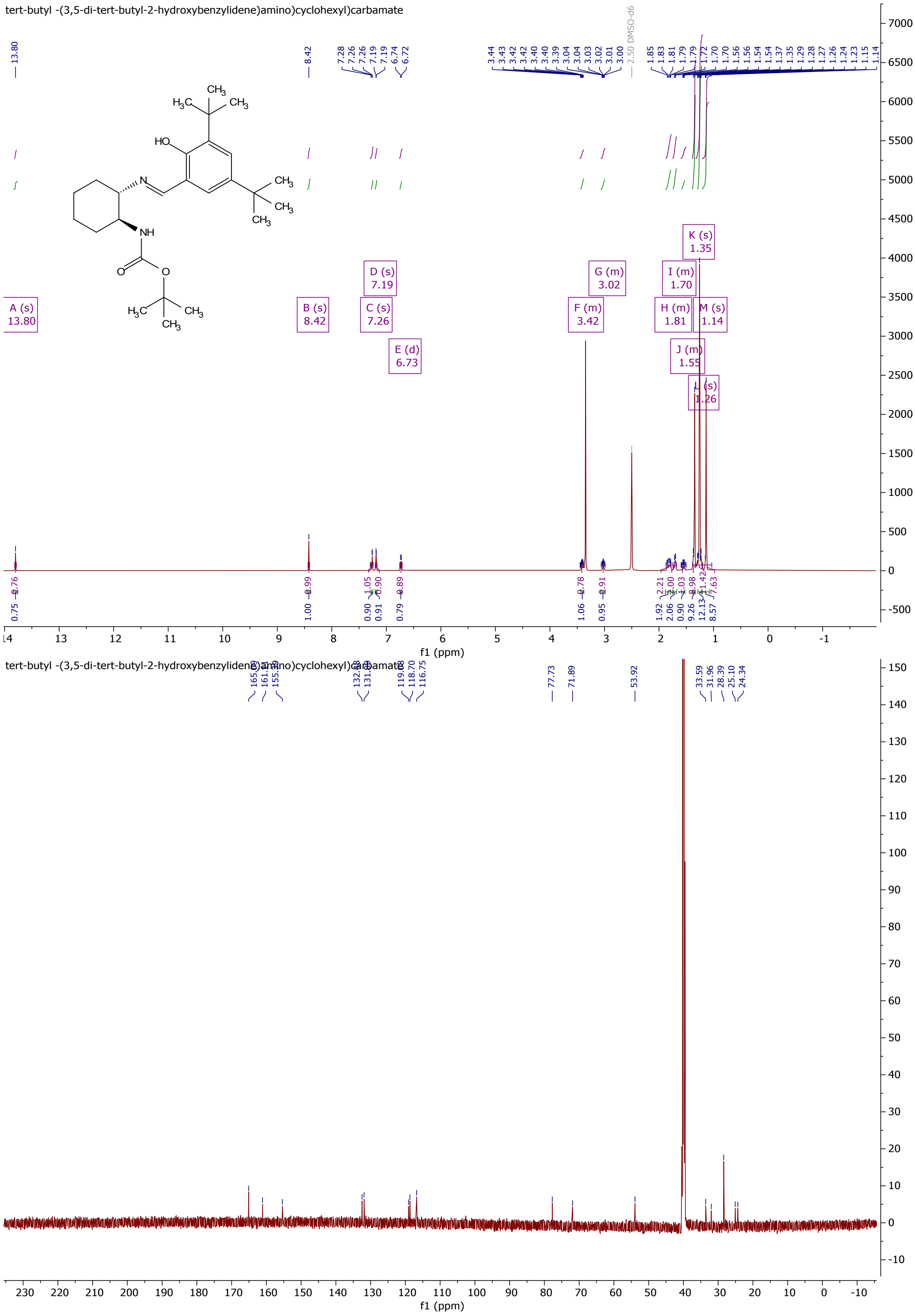

Figure S1. NMR charaterisation of **HL-Boc**:

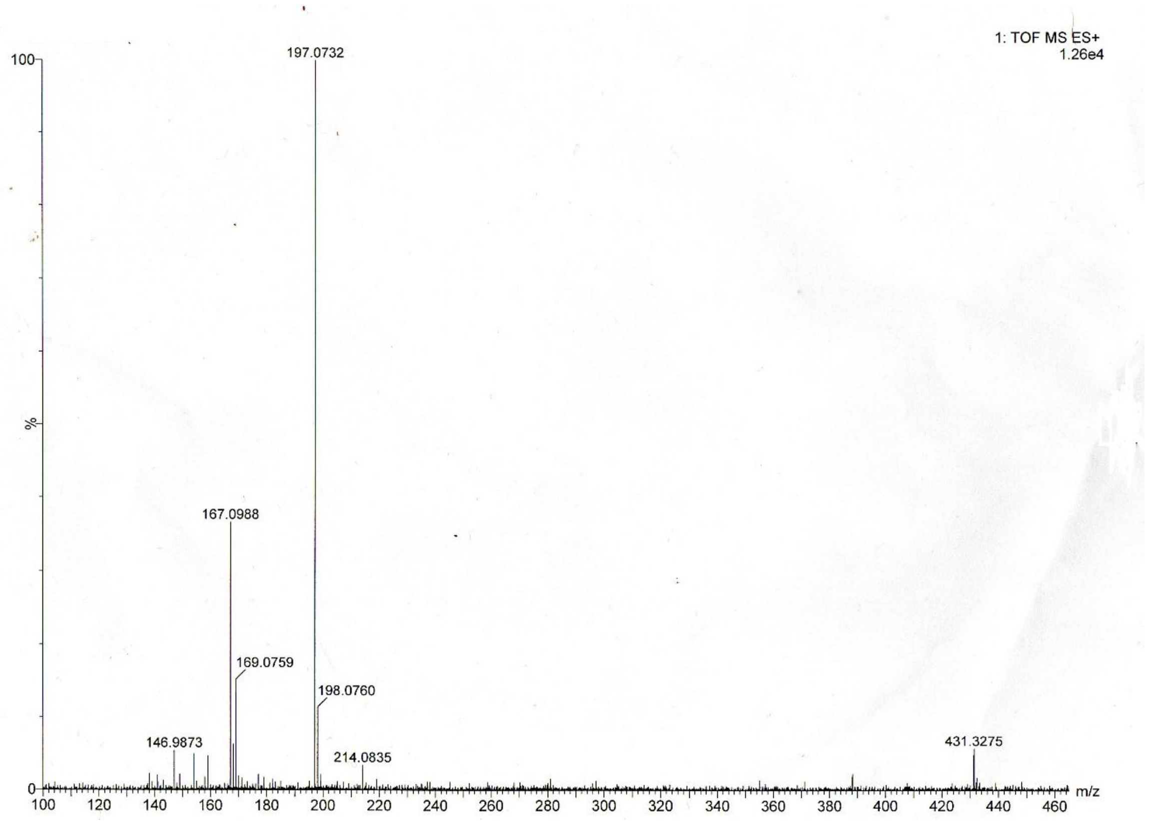

Figure S2. HRMS of **HL-Boc**

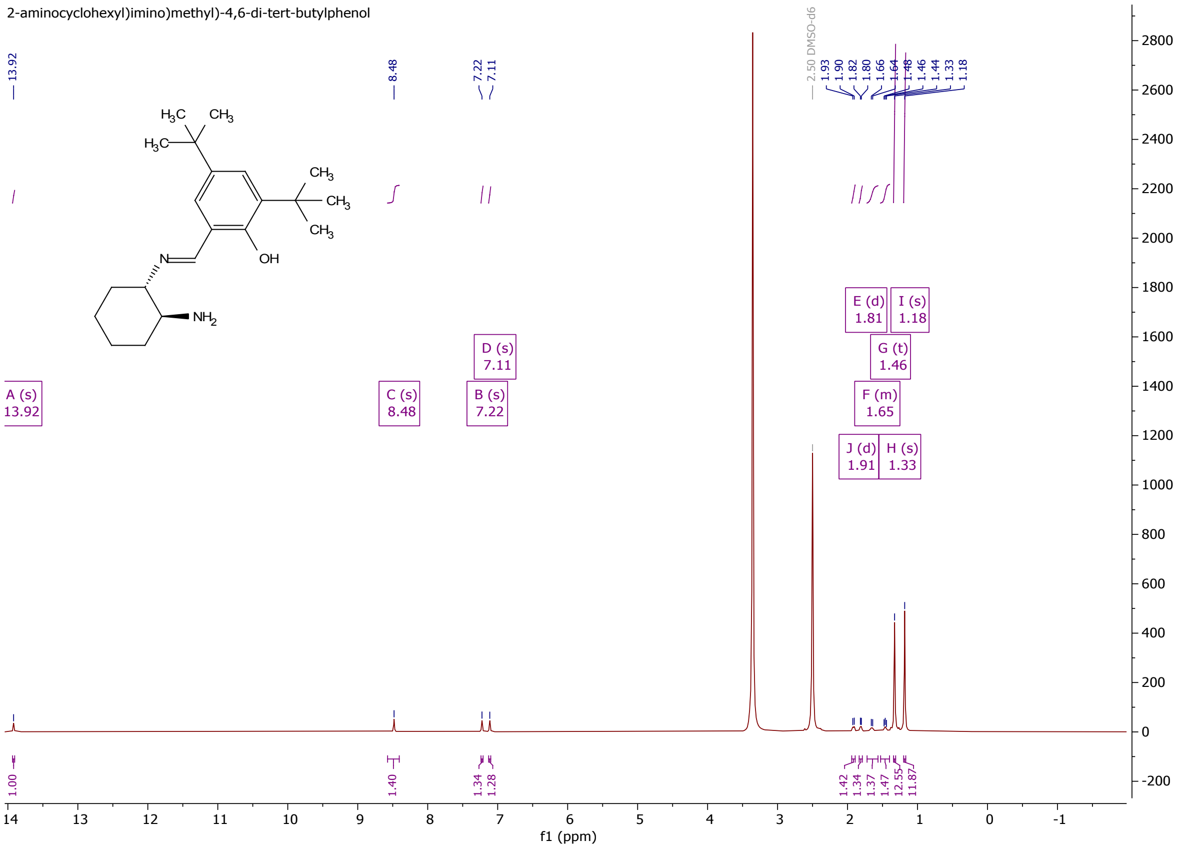

Figure S3. NMR charterisation of ligand (**HL**)

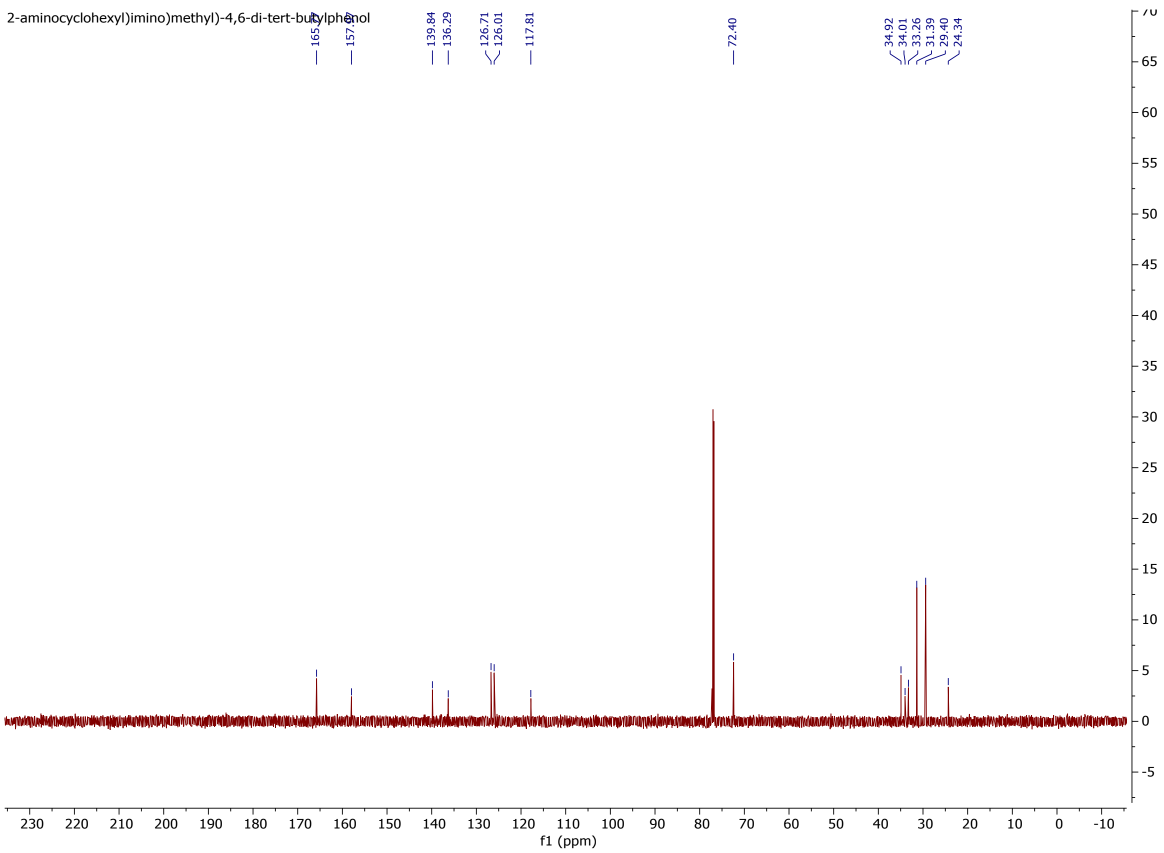

Figure S4. HRMS of ligand (**HL**).

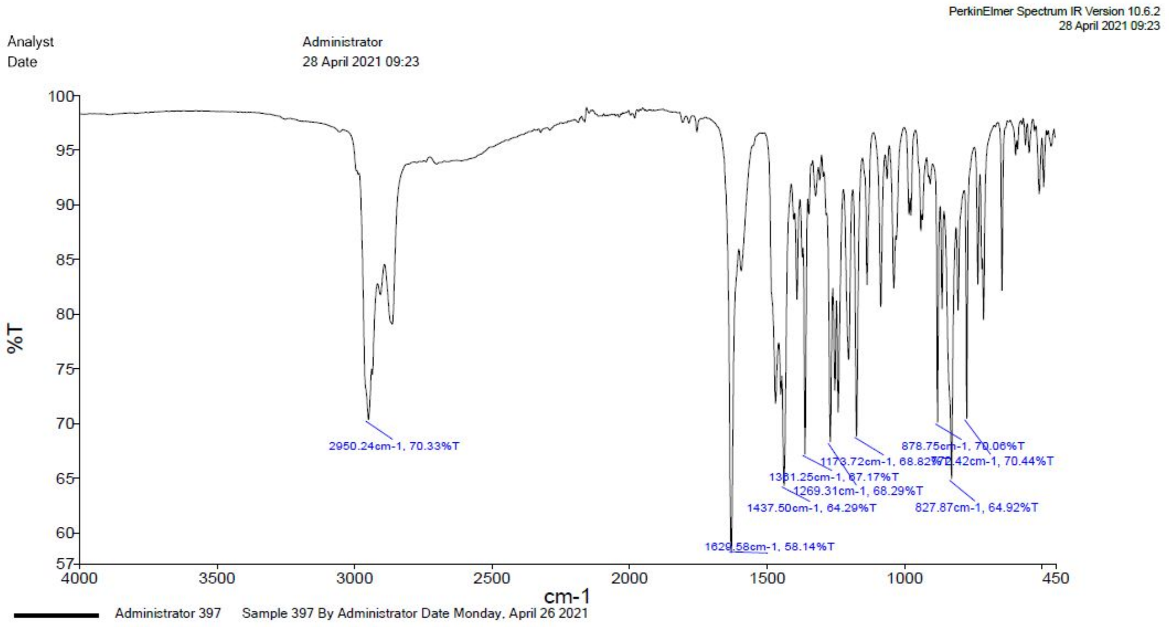

Figure S5. FT-IR of the ligand (**HL**).

Characterization of Catalyst

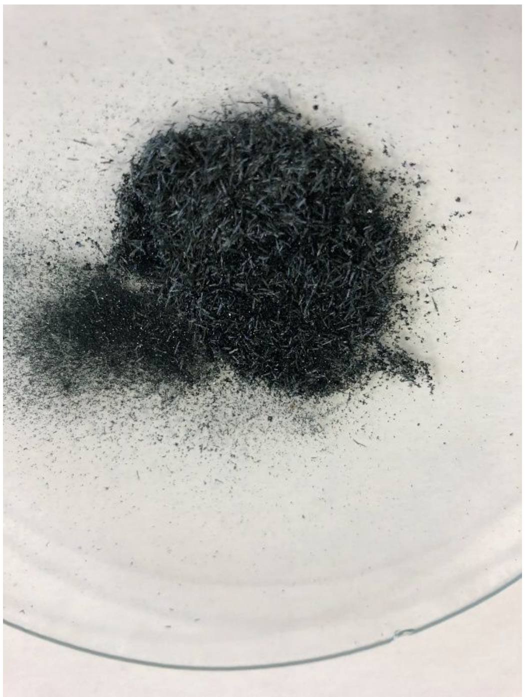

Figure S6: Gram scale synthesis of **1**

FTIR:

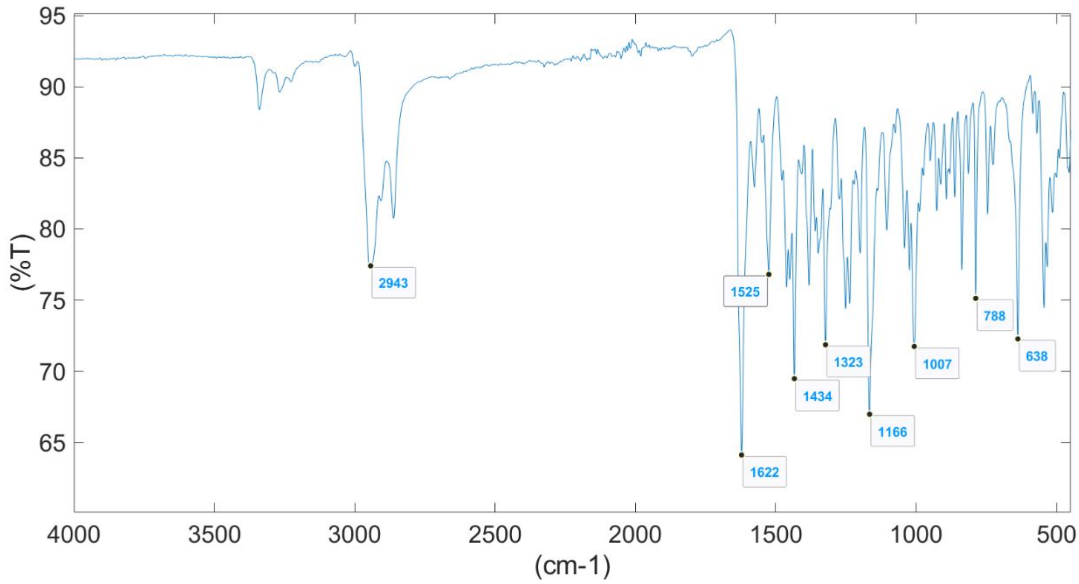

Figure S7. FT-IR of the catalyst (**1**)

HRMS:

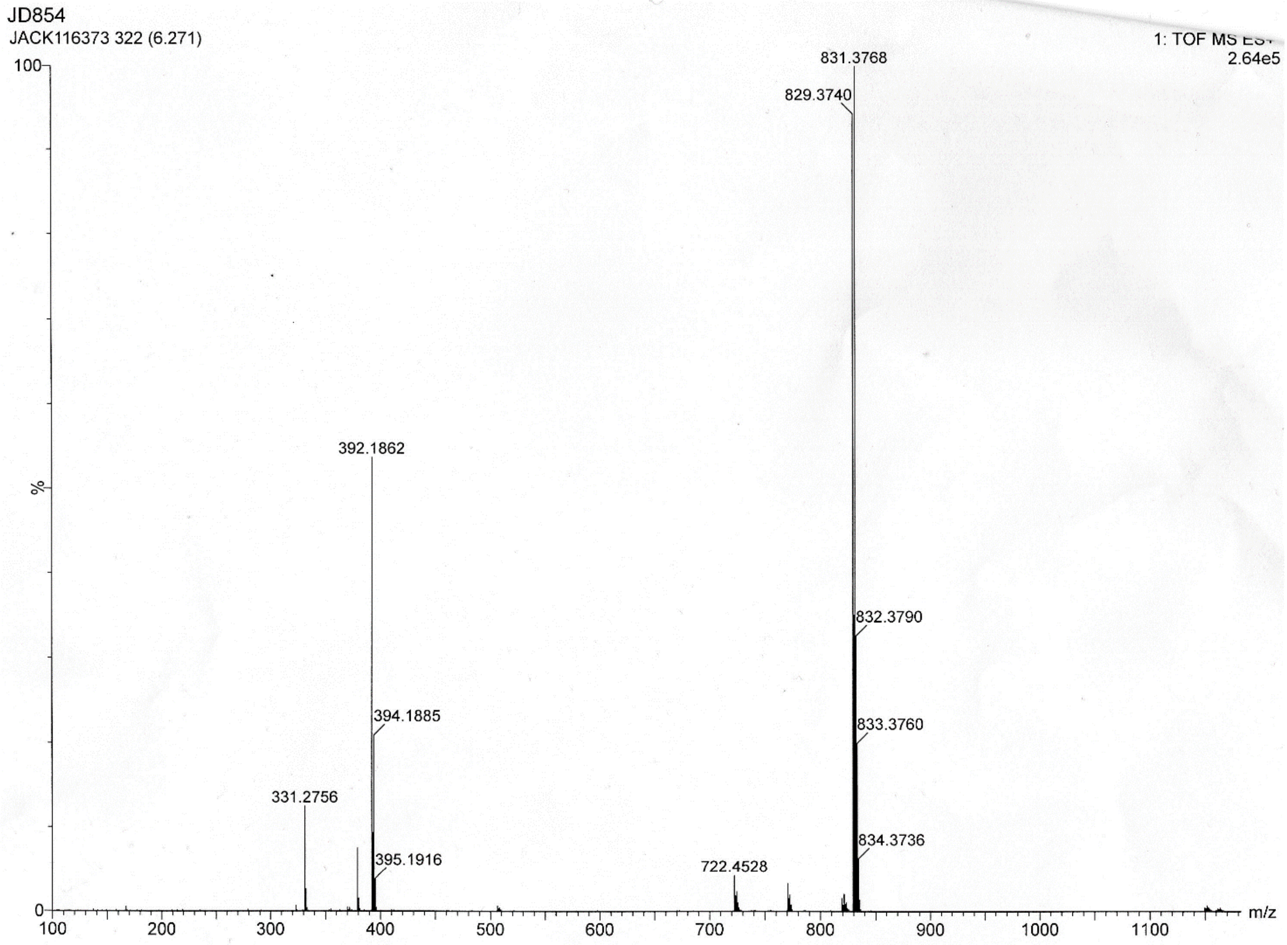

Figure S8. ESI-FTMS of the catalyst (**1**). m/z: ([(CuL)]$^+$), $C_{21}H_{33}CuN_2O$ calcd:392.1889, obs:392.1862. The peak at 831.3768 corresponds to the dimer.

TGA:

Thermal studies for **1**, were conducted up to 800°C. Compound **1** is thermal stable up to 205°C, and then a two-step decomposition starts and completes at 750°C yielding Cu_3O_2 residue calc.: 13.84%, theor.: 12.99%).

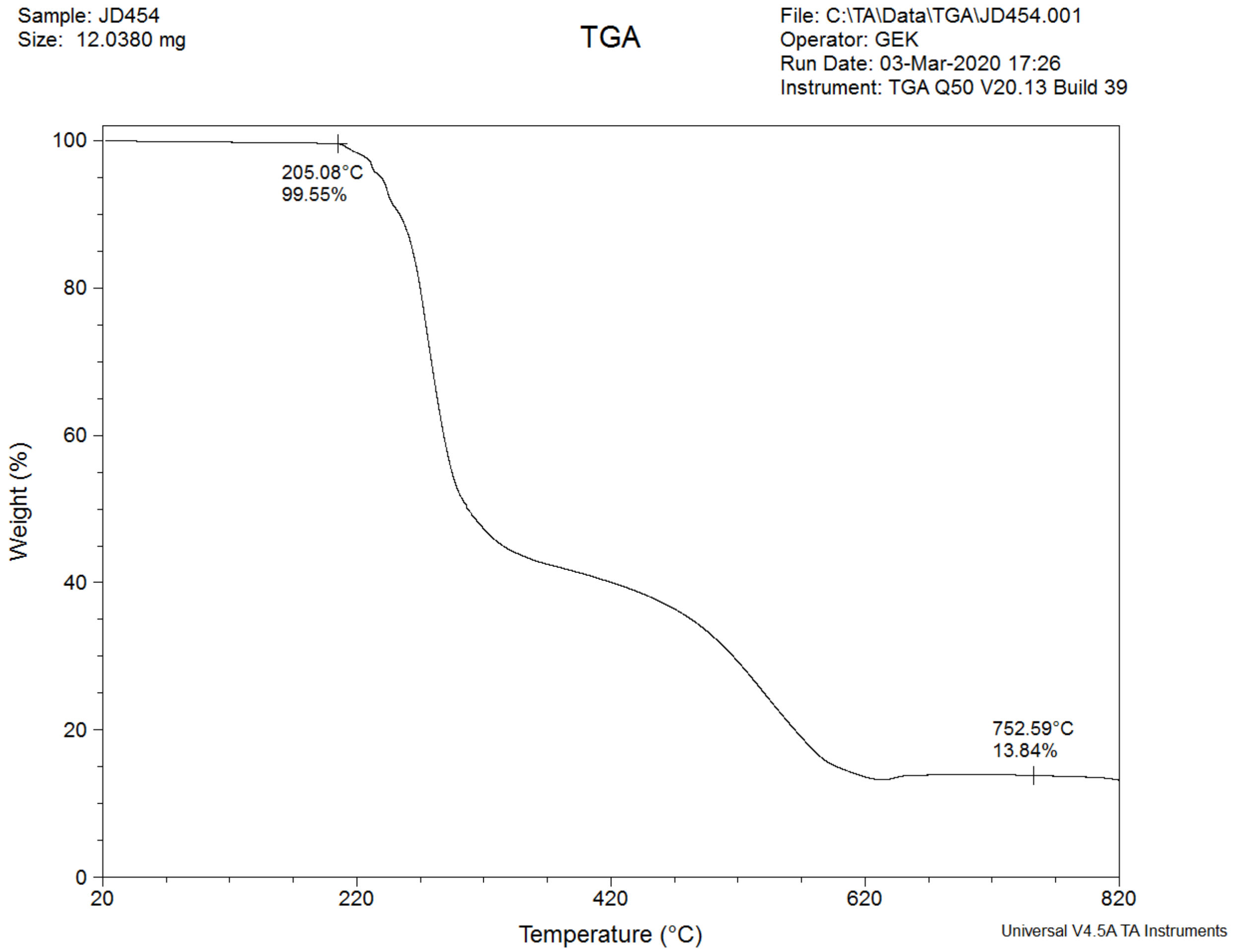

Figure S9. TGA of compound **1** (20-820˚C).

UV- Vis:

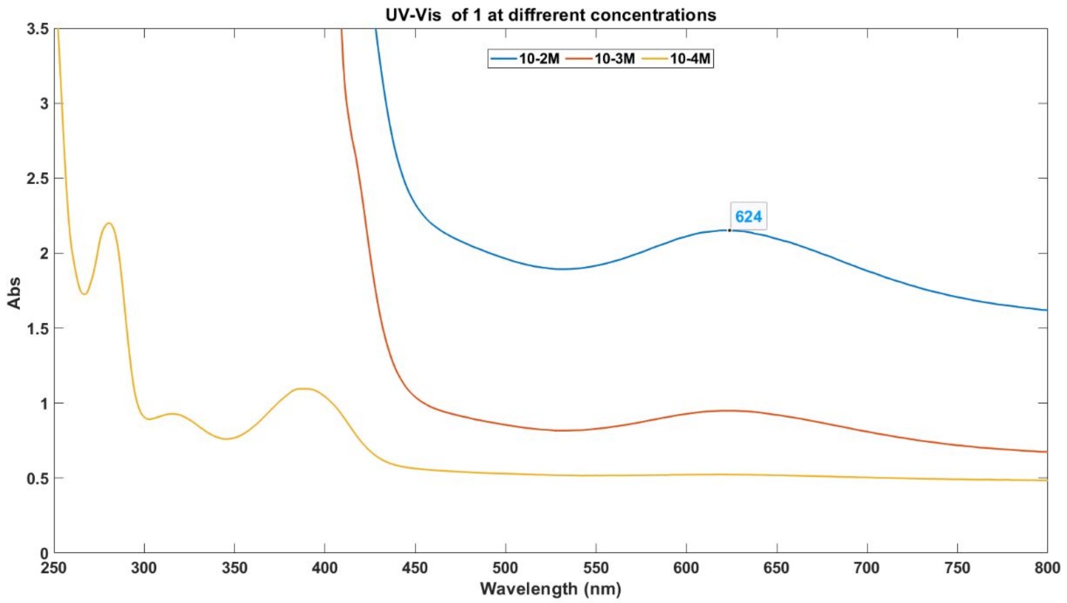

Figure S10. UV-Vis of **1** in DCM (0.01, 0.001 and 0.0001M) maxima at 281, 318 and 386 nm correspond to MLCT, maxima 624 nm are attributed to the Cu(II) centre of **1.**

CV Studies:

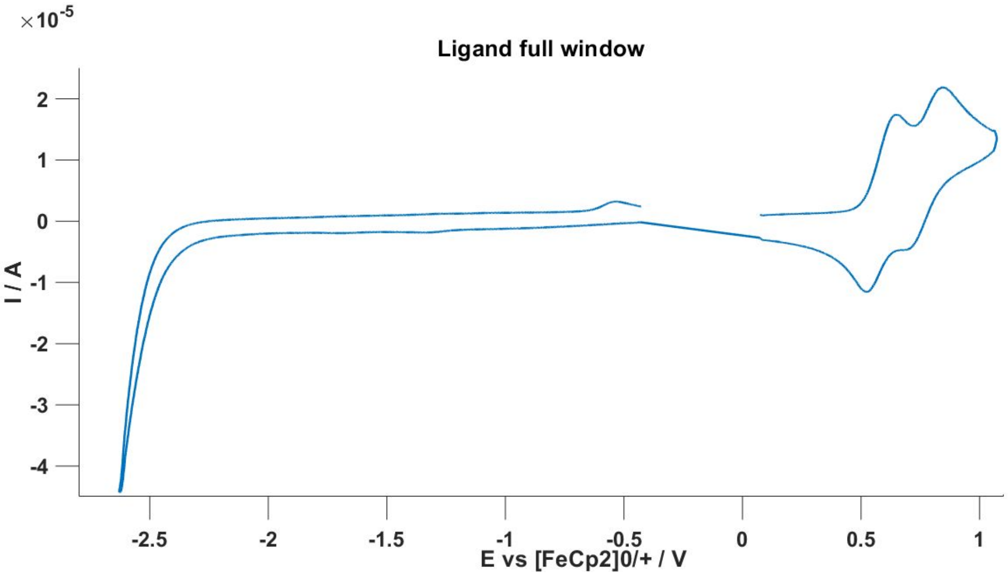

Figure S11. CV of **HL** in the presence of phenylacetylene

EPR spectra of 1

Initial attempts to fit the frozen-solution spectra of **1** based on a mononuclear species left the half-field and g = 1.85 transitions unaccounted for. These were modelled considering a dipole-coupled dinuclear species. Exchange interactions were excluded from the model, as the bridging of the solid state structure indicates the involvement of non-magnetic orbitals from both Cu^{II} ions.

Due to the axial **g**-tensors of the Cu^{II} ions, apart from the intermetallic distance, we also considered a φ_2 angular deviation of the spins' g-tensors from their *z*-axes (the $\mathbf{r}_{12}$ vector defined the *x*-axis of the molecular reference frame). This angular deviation was shown to influence the position of the $g < 2$ resonance, and its value was thus diagnostic to our fits. Due to remaining inconsistencies of the hyperfine pattern of the half-field transitions, the **g**- and **A**-frames of the Cu^{II} ions were considered non collinear, and an angle ϑ between their *z*-axes was also considered.

Best-fit parameters for the spectra according to this model were: g_z^{di} = 2.331, g_{xy}^{di} = 2.040, A_z^{di} = 622 MHz, r_{12} = 3.59 Å, φ_2 = 51.3°, $\vartheta_{g\text{-Aframe}}$ = 13.5°, σ_G = 2.8 mT, σ_L = 3 mT for the dinuclear species and g_z^{mono} = 2.236, g_{xy}^{mono} = 2.056, A_z^{mono} = 534 MHz, σ_G = 2.6 mT, σ_L = 4.6 mT, with weight^{di}:weight^{mono} = 1:10.9, indicating that ca. 85% of the catalyst decomposes to the mononuclear species upon dissolution.

The determined r_{12} distance and φ_2 angle were remarkably close to the crystallographically determined Cu···Cu2 distance (3.637 Å) and Cl1-Cu1-Cu2 angle (50.18°), corroborating the validity of our model and the identity of the dinuclear species.

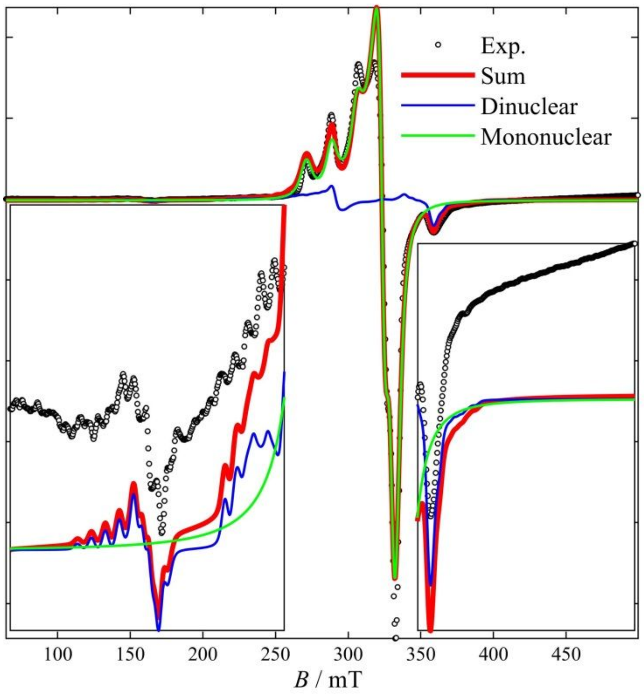

Figure S12. EPR spectrum of a frozen CH_2Cl_2 solution of **1**, and fit to a mixture of a mononononuclear and a dipole-coupled dinuclear complex. The insets show expansions of the weaker features due to half-field transitions (left) and the g = 1.85 absorption (right) from the dipole-coupled species. Experimental parameters: C = 0.75 mM, T = 100 K, f_{MW} = 9.313 GHz, P_{MW} = 9.89 mW, B_{mod} = 5 G_{pp}.

Fluid solution spectra could be nicely interpreted by considering only a mononuclear species with g_z^{mono} = 2.194, g_{xy}^{mono} = 2.051, $A_z^{monomer}$ = 729 MHz, σ_G = 4.3 mT, σ_L = 0.14 mT, t_{corr} = 2.82×10^{-11} s. A minor component at g = 2.015 (see below) was attributed to an impurity extrinsic to the Cu^{II} complex.

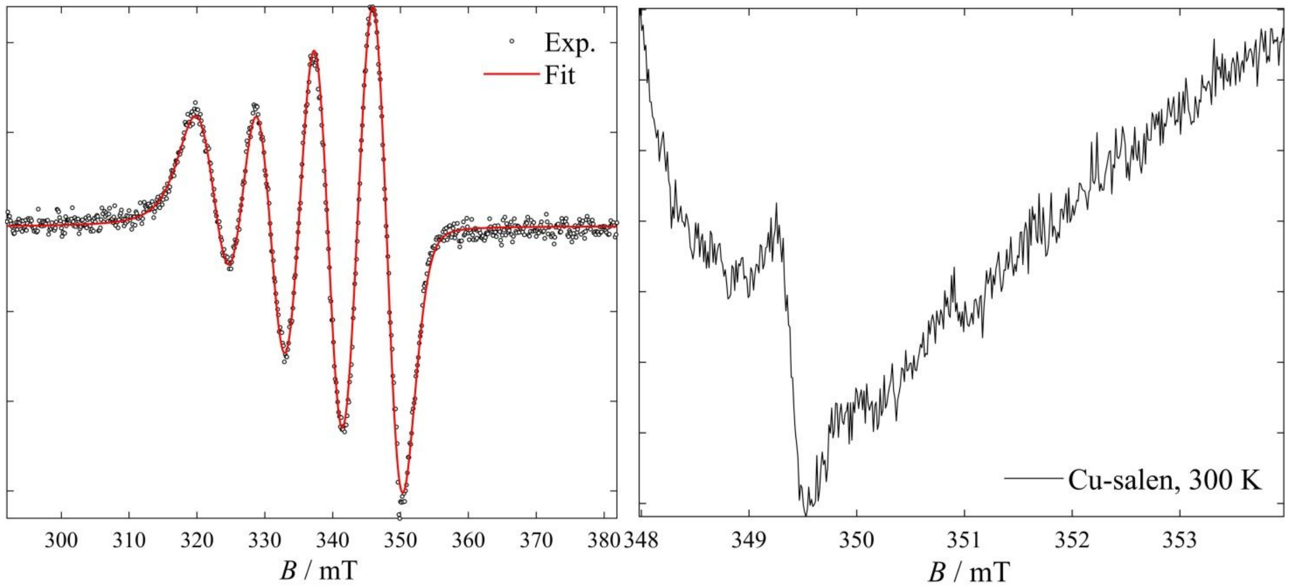

Figure S13. Full spectrum (left) and zoom in the *g* = 2 region (right) of the fluid CH_2Cl_2 solution of **1** (300 K). The fit was carried out according to the model described in the text. Experimental parameters are those of Figure 4.

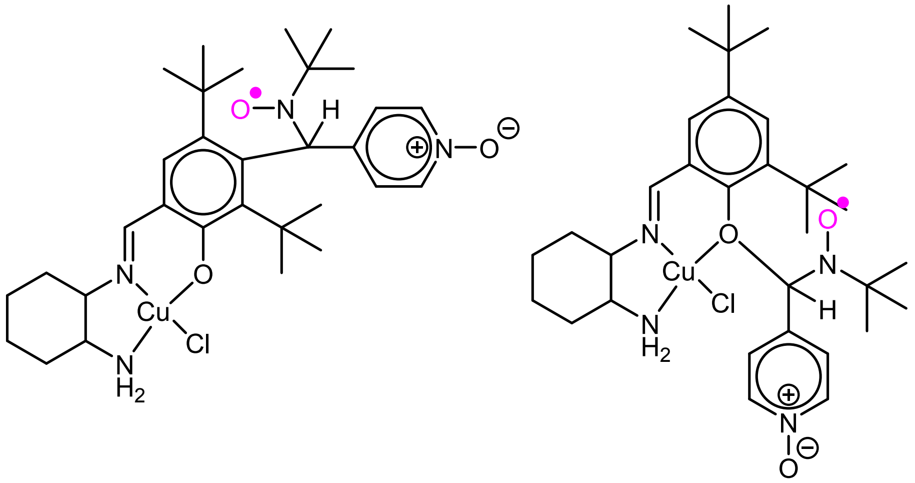

Scheme S2. Tentative examples of the Cu(II)- L* complex. L*is {POBN-salen}*

X-Ray crystallography

Variable temperature data for $\mathbf{1}^{100}$, $\mathbf{1}^{200}$, $\mathbf{1}^{298}$, $\mathbf{1}^{330}$ and data for **6** were collected at the National Crystallography Service, University of Southampton.[2] For **1**, a suitable green block shaped crystal (0.16×0.10×0.05) mm^3 was selected and mounted on a MITIGEN holder in perfluoro ether oil on a Rigaku FRE+ equipped with VHF Varimax confocal mirrors and an AFC12 goniometer and HyPix 6000HE detector and data were collected at *T* = 100(2)K, 200(2)K, 298(2)K and 330(2)K. Between each data collection the temperature was ramped at 360K/hour to the target temperature and then allowed to equilibrate for at least 30mins before a dataset was collected. . For **6**, Single blue blade-shaped crystals were supplied. A suitable crystal (0.07×0.03×0.01) mm^3 was selected and mounted on a MITIGEN holder in perfluoroether oil on a Rigaku 007HF equipped with Varimax confocal mirrors and an AFC11 goniometer and HyPix 6000HE detector. The crystal was kept at a steady *T* = 100(2) K during data collection. The data were processed with CrysAlisPro and solved by intrinsic phasing methods with SHELXT.[3] All crystal structures were then refined on Fo^2 by full-matrix least-squares refinements using SHELXL.[3] Geometric/crystallographic calculations were performed using PLATON,[4]Olex2,[5] and WINGX[6] packages; graphics were prepared with Crystal Maker.[7] Structures $\mathbf{1}^{100}$, $\mathbf{1}^{200}$, $\mathbf{1}^{298}$, $\mathbf{1}^{330}$ and **6** have been given CCDC deposition numbers 2080821 – 2080825.

Geometrical calculations

Table S1. Metal centre geometry calculations

	Structure [ML_5]				
	JTBPY-5	SPY-5	TBPY-5	vOC-5	PP-5
**Cu(1)**					
100K	10.689	**4.578**	6.420	4.982	25.446
200K	10.465	**4.916**	6.150	5.291	26.169
298K	10.395	**5.185**	6.037	5.551	26.746
330K	27.993	21.611	23.962	26.190	34.320
Recovered (120K)	13.153	**4.859**	8.706	6.006	22.694
**Cu(2)**					
100K	10.184	**6.160**	10.056	**6.191**	22.389
200K	10.157	6.050	10.071	**6.032**	22.471
298K	10.102	6.040	10.095	**5.964**	22.550
330K	21.506	19.530	20.852	19.678	28.559
Recovered (120K)	13.153	**4.859**	8.706	6.006	22.694
	Structure [ML_4]				
**Cu(1)**	vTBPY-4	SS-4	T-4	SP-4	
100K	26.359	15.384	26.194	**3.886**	
200K	24.742	14.513	24.756	**4.123**	
298K	23.724	14.028	23.886	**4.378**	
330K	34.082	17.479	34.277	**1.887**	
Recovered (120K)	29.969	15.519	29.542	**0.965**	
**Cu(2)**					
100K	33.955	17.382	34.071	**2.069**	
200K	34.116	17.497	34.272	**1.944**	
298K	34.150	17.511	34.341	**1.896**	
330K	23.598	14.031	23.815	**4.441**	
Recovered (120K)	29.969	15.519	29.542	**0.965**	

Shape Geometry calculations[8]

JTBPY-5	5 D3h	Johnson trigonal bipyramid J12	vTBPY-4	4 C3v	Vacant trigonal bipyramid
SPY-5	4 C4v	Spherical square pyramid	SS-4	3 C2v	Seesaw
TBPY-5	3 D3h	Trigonal bipyramid	T-4	2 Td	Tetrahedron
vOC-5	2 C4v	Vacant octahedron	SP-4	1 D4h	Square
PP-5	1 D5h	Pentagon			

Table S2. Selected Bond Distances for Various Temperatures

Atom	Atom	Length/Å			
		**100K**	**200K**	**298K**	**330K**
**Cu(1)**	**Cu(2)**	**3.6639(6)**	**3.7042(6)**	**3.7526(6)**	**3.7636(8)**
Cu(1)	Cl(1)	2.3166(7)	2.3104(8)	2.3057(10)	2.2874(12)
Cu(1)	O(1)	1.8771(19)	1.871(2)	1.870(2)	1.879(3)
Cu(1)	N(2)	1.958(2)	1.951(3)	1.949(3)	1.948(3)
Cu(1)	N(1)	1.988(3)	1.988(3)	1.988(4)	2.002(4)
Cu(1)	Cl(2)	**2.8345(10)**	**2.8984(12)**	**2.9562(14)**	**2.9692(17)**
Cu(2)	Cl(2)	2.2906(8)	2.2890(9)	2.2887(10)	2.3057(11)
Cu(2)	O(2)	1.886(2)	1.885(2)	1.881(2)	1.875(3)
Cu(2)	N(4)	1.951(3)	1.948(3)	1.951(3)	1.948(3)
Cu(2)	N(3)	2.008(3	2.004(3)	2.003(3)	1.993(4)

Table S3. Bond Valence Sum calculations for all compoundsx

**1** (100K)	$CuON_2Cl_2$	**2.0842**
**1**(200K)	$CuON_2Cl_2$	**2.0881**
**1**(298K)	$CuON_2Cl_2$	**2.0857**
**1**(330K)	$CuON_2Cl_2$	**2.0736**
**1** (100K)	$CuON_2Cl$	**1.9784**
**1**(200K)	$CuON_2Cl$	**1.9910**
**1**(298K)	$CuON_2Cl$	**1.9945**
**1**(330K)	$CuON_2Cl$	**2.0014**
**Recovered**	CuO_3N_2	**2.0686**

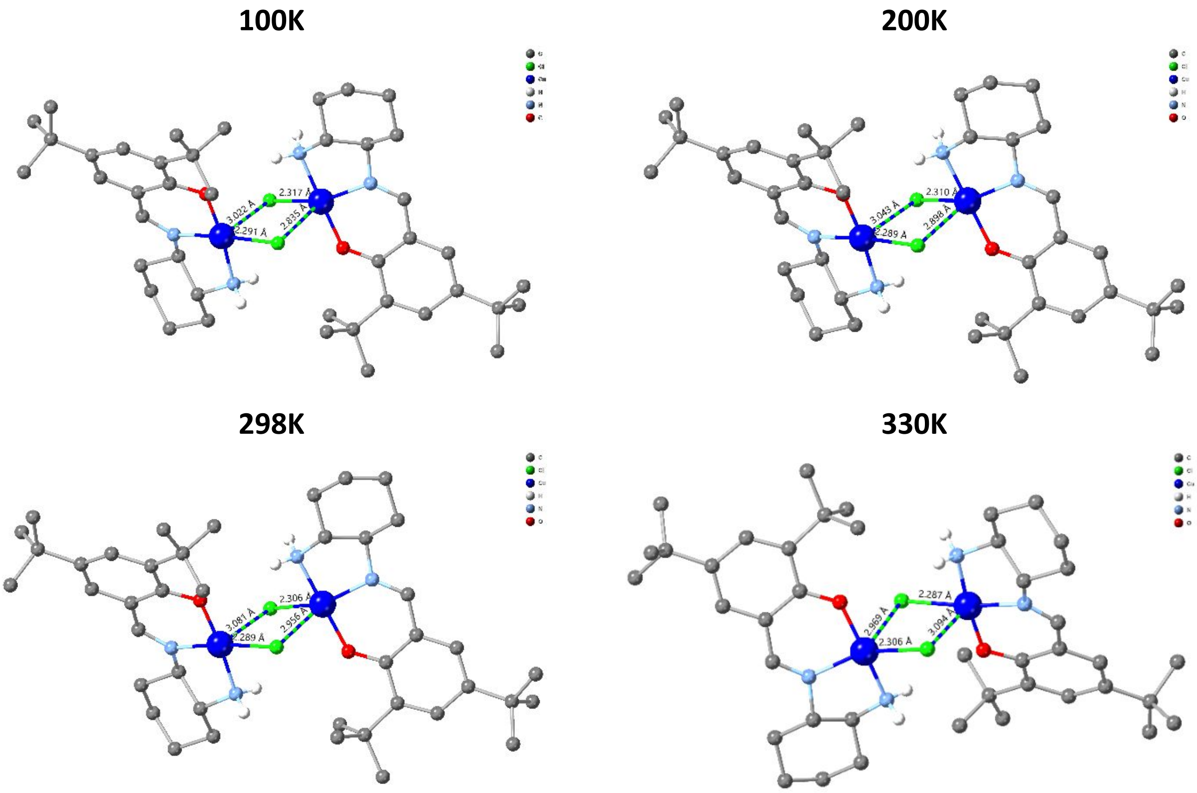

Figure S14: Solid-state structure at various temperatures.

Screening of (1) for the synthesis of propargylamines.

Table S4: Solvent screen with **(1).**

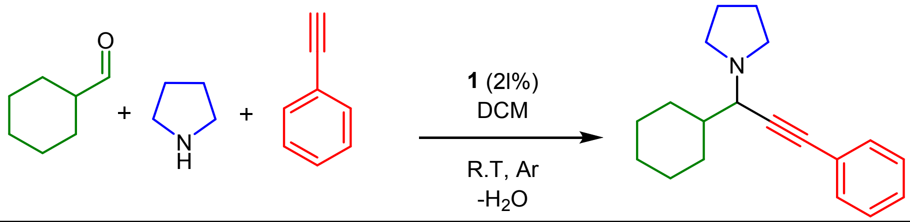

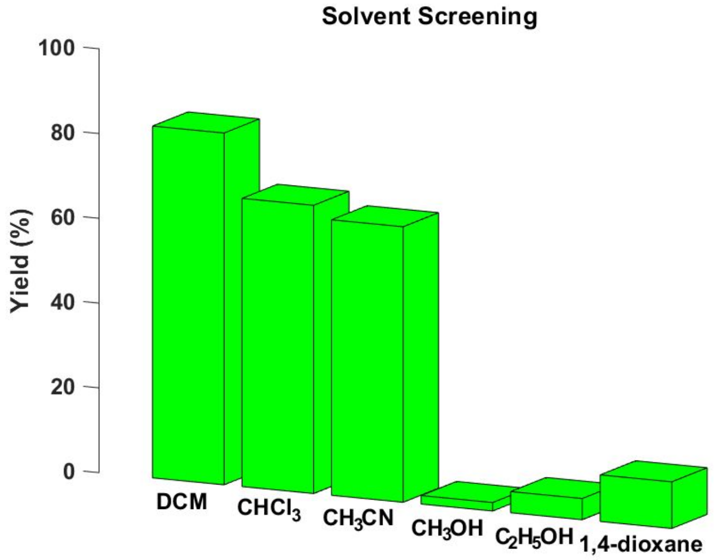

[a] ^{1}H-NMR yield and durene as an internal standard; [b]Reaction conditions, catalyst (2 mol%), 1.0 mmol aldehyde, 1.1 mmol amine, 1.2 mmol alkyne, 2 mL solvent, 12 hours, room temperature. In the presence of molecular sieves 4Å, Ar atmosphere.

Table S5. Condition screens with **1.**

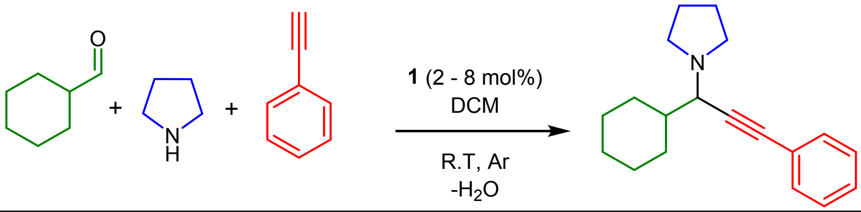

Condition Screening

	1<br>Loading (%)	2<br>Concentration (M)	3<br>Time (h)	4<br>Yield (%)
1	8	0.5	12	26
2	4	0.5	12	45
3	2	0.5	12	83
4	1	0.5	12	65
5	0.5	0.5	12	38
6	2	0.5	16	94
7	2	0.5	8	75
8	2	0.5	6	69
9	2	0.5	4	62
10	2	0.5	2	45
11	2	0.2	12	65
12	2	0.4	12	81

[a]NMR yield calculated using ^{1}H-NMR and durene as an internal standard; [b]Reaction conditions, catalyst (2-8 mol%), 1.0 mmol aldehyde, 1.1 mmol amine, 1.2 mmol alkyne, 2-16 hours, room temperature, Concentration 0.2 - 0.5 M, In the presence of molecular sieves 4Å, Ar atmosphere.

Standard procedure for screening reactions:

Aldehyde (1 mmol, 1 eq.), amine (1.1 mmol, 1.1 eq.), alkyne (1.2 mmol, 1.2 eq.) and activated, crushed, 4Å molecular sieves (100 mg) were combined in dry dichloromethane (2 mL) under argon. **(1)** (17 mg, 2 mol%) was added and the reaction was stirred at R.T under Ar for 16 hours. The reaction mixture was filtered, then passed over a silica plug, the silica plug was washed with DCM (3x 5 mL) and the filtrate was concentrated. The sample was dissolved in $CDCl_3$ and durene (ca. 10 mg) was added. NMR yields were then based comparing the integrals arising from the signals corresponding to the product and durene.

Theoretical calculations

Geometry optimization was carried out using the Gaussian09[9] program at the OLYP/def2-SVP D3 with PCM implicit solvation [2–9] level of theory.[10–17] To obtain the singlet state, spin-restricted calculations were performed constraining the projection of the total electronic spin along a reference axis to 0, and for the doublet in the unrestricted formalism and setting the projection to 1/2. Frequency calculations were conducted to determine if each stationary point corresponds to a minimum. The Bader charge analysis was used[18] to determine oxidation states. The Jmol program was used for vizualisation purposes.[19]

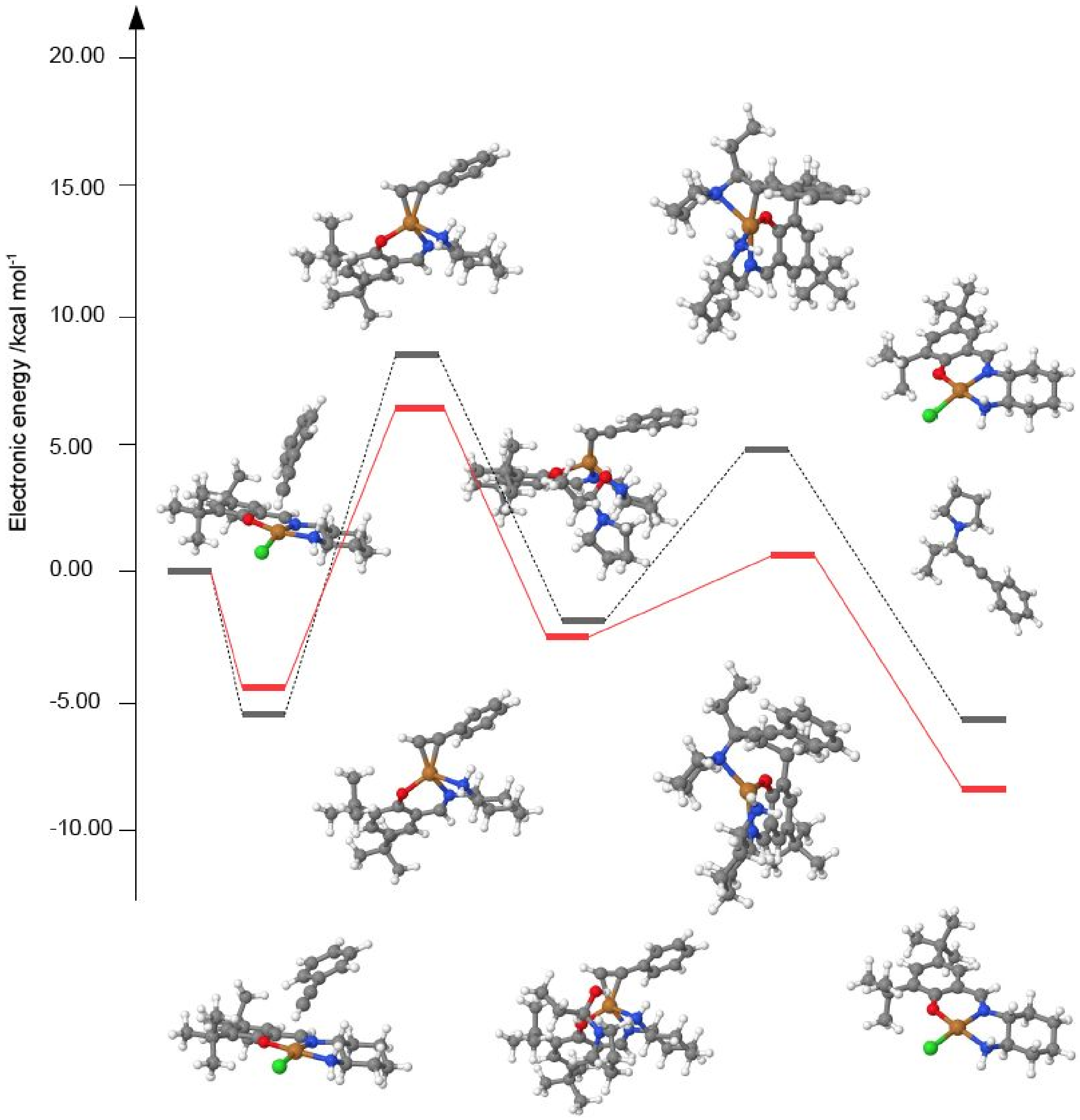

Figure S15 Comparison of results using the OLYP (upper figures, black lines) and the wB97XD (lower figures, red lines) xc functionals.

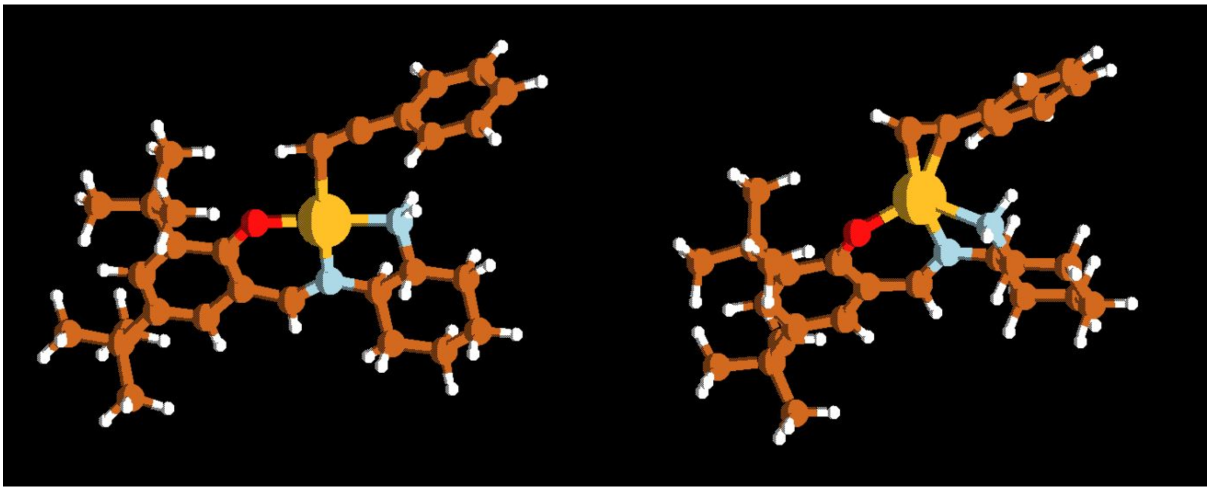

Figure S16 Comparison of structures with (left) and without (right) counterpoise correction at the OLYP/def2SVP D3 (PCM) level.

Cartesian coordinates

catalyst.xyz

Cu	1.773537	1.300362	-0.135894
Cl	2.175442	3.565616	0.052940
O	-0.172398	1.483024	-0.192235
N	1.646964	-0.696778	-0.245943
N	3.825666	0.920430	-0.095098
H	4.225368	1.078327	-1.026275
H	4.277803	1.593915	0.528118
C	3.996147	-0.474873	0.343492
H	3.714559	-0.495914	1.412431
C	5.426391	-1.009514	0.189845
H	5.733479	-0.906845	-0.868796
H	6.114098	-0.380331	0.782545
C	5.520544	-2.479170	0.616028
H	6.547291	-2.852193	0.453602
H	5.326785	-2.559146	1.702731
C	4.510207	-3.339580	-0.149743
H	4.552908	-4.386958	0.198213
H	4.783250	-3.357328	-1.222119
C	3.073734	-2.815300	-0.001517
H	2.740706	-2.917007	1.048227
H	2.399011	-3.436168	-0.614093
C	2.962782	-1.338982	-0.414477
H	3.210398	-1.263020	-1.494432
C	0.526890	-1.364263	-0.237779
H	0.574989	-2.462728	-0.310319
C	-0.811559	-0.839590	-0.142257
C	-1.870989	-1.786173	-0.091284
H	-1.598631	-2.846473	-0.129500
C	-3.207184	-1.409782	0.003439
C	-3.461090	-0.011228	0.057633

H	-4.497853	0.308583	0.139304
C	-2.483375	0.988079	0.015981
C	-1.095928	0.581012	-0.106691
C	-4.328483	-2.478614	0.042937
C	-5.736662	-1.856504	0.159417
H	-5.852921	-1.264926	1.083145
H	-6.497258	-2.655327	0.184420
H	-5.974913	-1.205345	-0.698478
C	-4.284565	-3.319336	-1.259137
H	-4.435119	-2.680139	-2.146487
H	-5.079101	-4.086896	-1.254848
H	-3.321342	-3.841642	-1.381482
C	-4.116998	-3.409829	1.264438
H	-3.149626	-3.936963	1.221290
H	-4.909322	-4.178302	1.310403
H	-4.145124	-2.836805	2.207553
C	-2.848170	2.493481	0.096545
C	-2.391530	3.225640	-1.193909
H	-1.301099	3.175717	-1.321344
H	-2.683137	4.290550	-1.150473
H	-2.870712	2.783082	-2.085239
C	-4.370392	2.719267	0.241145
H	-4.936444	2.328992	-0.621896
H	-4.577368	3.801132	0.302373
H	-4.776973	2.258209	1.157448
C	-2.165091	3.135631	1.334712
H	-2.485375	2.630797	2.263397
H	-2.451182	4.199631	1.416546
H	-1.069518	3.083565	1.267255

catalyst_alkyne.xyz

C	1.002205	-0.309160	2.866848
C	2.083445	-1.187349	3.134851
C	2.745005	-1.100154	4.384667
C	2.332957	-0.158993	5.335781
C	1.261388	0.706212	5.061657
C	0.599348	0.627203	3.825535
C	2.479814	-2.139944	2.142310
C	2.775044	-2.937201	1.259774
Cu	1.384309	-0.949487	-1.215576
O	-0.475480	-1.316715	-0.736083
C	-1.466290	-0.500122	-0.575807
C	-1.282778	0.934168	-0.454382
C	-2.397792	1.782539	-0.215879
C	-3.696133	1.295763	-0.097048
C	-3.854127	-0.110652	-0.238226
C	-2.817813	-1.019024	-0.475874
C	0.008605	1.567733	-0.547946
N	1.151023	1.009486	-0.837863

C	2.430077	1.740148	-0.775881
C	2.397364	3.230408	-1.158065
C	3.800248	3.854903	-1.108653
C	4.808916	3.089306	-1.971792
C	4.845794	1.604039	-1.591495
C	3.446174	0.979809	-1.655962
N	3.403078	-0.442390	-1.278977
C	-3.085537	-2.539469	-0.625271
C	-2.319404	-3.332013	0.467812
C	-4.880864	2.259549	0.162927
C	-6.227140	1.517960	0.309494
C	-4.628881	3.050610	1.472481
C	-5.006927	3.252130	-1.021842
C	-4.585039	-2.886316	-0.478631
C	-2.633281	-3.014063	-2.032260
H	3.843806	-0.611110	-0.368336
H	3.876185	-1.046423	-1.955650
H	3.076948	1.029475	-2.697280
H	5.239312	1.489430	-0.563130
H	5.525536	1.042628	-2.257019
H	5.816600	3.529648	-1.869385
H	4.532155	3.187424	-3.038866
H	3.740418	4.909776	-1.430724
H	4.153890	3.870645	-0.059898
H	1.972444	3.323824	-2.174758
H	1.731425	3.789111	-0.479939
H	2.785070	1.659021	0.272943
H	-0.003511	2.649397	-0.335111
H	-2.203549	2.856841	-0.130232
H	-4.861630	-0.512144	-0.156798
H	-6.498600	0.964515	-0.605183
H	-7.034647	2.244241	0.503241
H	-6.217385	0.805930	1.152049
H	-4.533113	2.367424	2.334311
H	-5.466938	3.740465	1.677363
H	-3.709739	3.657305	1.421133
H	-4.094368	3.857219	-1.151871
H	-5.847338	3.950387	-0.858413
H	-5.191299	2.714946	-1.968393
H	-1.232209	-3.208947	0.367977
H	-2.551455	-4.409204	0.388585
H	-2.618945	-2.997808	1.477072
H	-4.983302	-2.615827	0.514309
H	-4.722954	-3.974281	-0.597962
H	-5.207000	-2.394556	-1.245961
H	-3.179186	-2.469316	-2.822817
H	-2.848917	-4.090462	-2.156969
H	-1.555298	-2.865258	-2.183906
H	2.979874	-3.596924	0.431530

H	3.579754	-1.775036	4.601050
H	2.851840	-0.101260	6.299643
H	0.942534	1.439994	5.810935
H	-0.239355	1.297975	3.605538
H	0.490957	-0.373803	1.902736
Cl	1.802112	-2.987306	-2.240489

alkyne_capture.xyz

C	1.982256	3.142055	-0.390819
C	2.031804	1.611302	-0.519645
C	2.474951	1.190220	-1.958577
C	3.787887	1.885695	-2.364274
C	3.739686	3.407682	-2.192777
C	3.323321	3.786769	-0.767446
N	0.819995	0.859458	-0.198099
Cu	1.042810	-1.145608	-0.337163
C	1.723479	-2.912305	0.207100
C	2.590248	-2.126877	0.683989
C	3.755140	-1.543216	1.288961
N	2.530947	-0.274640	-2.061919
C	-0.341340	1.413302	-0.007624
C	-1.625610	0.740686	0.086803
C	-1.842174	-0.632728	-0.349081
C	-3.212486	-1.123883	-0.318025
C	-4.221874	-0.277565	0.154816
C	-4.025035	1.058620	0.594198
C	-2.716164	1.534108	0.529042
O	-0.887441	-1.393666	-0.775568
C	-5.171544	1.962639	1.112837
C	-4.879095	2.381389	2.576886
C	-3.530468	-2.568188	-0.789349
C	-5.033301	-2.913759	-0.682997
C	-5.277658	3.231535	0.228328
C	-6.543490	1.254162	1.087680
C	-3.126355	-2.737668	-2.278188
C	-2.761618	-3.591587	0.088663
H	3.442783	-0.617453	-1.751896
H	2.440029	-0.567638	-3.035545
H	1.667735	1.526388	-2.636159
H	4.605780	1.473746	-1.740914
H	4.025070	1.614672	-3.409434
H	4.722253	3.848609	-2.440788
H	3.015663	3.836704	-2.912214
H	3.250434	4.884582	-0.664622
H	4.104664	3.458835	-0.054818
H	1.196379	3.547952	-1.054357
H	1.701124	3.417941	0.641321
H	2.811702	1.237251	0.171018
H	-0.407402	2.512259	0.077839

H	-2.493647	2.563973	0.831871
H	-5.235201	-0.673089	0.186947
H	-6.835624	0.954341	0.067040
H	-7.325041	1.935931	1.464720
H	-6.558065	0.354636	1.726089
H	-4.801147	1.496115	3.231984
H	-5.686249	3.025298	2.970799
H	-3.935075	2.944536	2.663245
H	-4.347066	3.822558	0.240017
H	-6.091354	3.890087	0.582443
H	-5.490943	2.963238	-0.821112
H	-1.677054	-3.423720	0.036371
H	-2.973415	-4.623731	-0.245564
H	-3.074686	-3.510907	1.144847
H	-5.403929	-2.859519	0.354786
H	-5.198149	-3.947236	-1.033473
H	-5.660698	-2.254627	-1.307096
H	-3.696264	-2.040421	-2.917865
H	-3.345473	-3.764562	-2.623732
H	-2.054649	-2.543329	-2.423377
H	1.227147	-3.870968	0.076324
C	5.052487	-1.937587	0.870266
C	6.188283	-1.364487	1.453914
C	6.058318	-0.396929	2.463563
C	4.778210	-0.001047	2.886418
C	3.635925	-0.559153	2.303176
H	5.156629	-2.694793	0.086205
H	7.183942	-1.678939	1.119674
H	6.950774	0.047931	2.918266
H	4.669472	0.753262	3.674380
H	2.638292	-0.240791	2.622156

catalyst_alkyne_Cu.xyz

C	3.322959	1.725634	-2.153879
N	3.445597	0.338484	-1.739860
C	3.274961	-0.490352	-2.925162
C	2.287740	0.312174	-3.814660
C	2.111647	1.669613	-3.091739
C	4.411785	-0.062721	-0.732370
C	5.846797	0.454656	-0.944020
C	6.880507	-0.167884	-0.003870
O	4.000701	0.349666	0.562552
N	1.685310	2.483194	1.709078
C	1.133686	3.650187	1.012242
C	-0.406491	3.574921	1.067985
C	-1.021527	4.745013	0.272658
C	-0.498919	6.103671	0.758083
C	1.032903	6.171491	0.726110
C	1.656175	5.007397	1.506660

N	-0.829220	2.254857	0.565948
C	-2.093032	1.953894	0.673409
C	-2.801466	0.783213	0.151845
C	-2.254462	-0.170679	-0.761334
C	-3.022312	-1.289372	-1.188783
C	-4.332221	-1.399300	-0.677649
C	-4.926043	-0.475198	0.206599
C	-4.133479	0.611190	0.594576
O	-0.996173	0.085594	-1.194929
Cu	0.566629	0.770844	0.974574
C	1.162318	-0.998169	1.230379
C	1.646207	-2.129637	1.453206
C	2.236789	-3.406301	1.703454
C	3.650739	-3.551390	1.777876
C	4.235544	-4.799809	2.018581
C	3.435245	-5.941014	2.196490
C	2.037417	-5.817064	2.129786
C	1.443724	-4.572850	1.886659
C	-6.372808	-0.620412	0.738711
C	-7.084377	-1.872876	0.183798
C	-2.466118	-2.338493	-2.194167
C	-1.235170	-3.068514	-1.590059
C	-3.502976	-3.436974	-2.526505
C	-2.099126	-1.651839	-3.539055
C	-6.341186	-0.732094	2.284679
C	-7.201199	0.623294	0.325702
H	1.607325	2.592176	2.724217
H	2.678910	2.350883	1.503887
H	1.412103	3.531249	-0.050285
H	1.420858	5.108705	2.584086
H	2.758121	5.023794	1.423146
H	1.386066	7.135956	1.133388
H	1.378637	6.131157	-0.324737
H	-0.933026	6.909766	0.139316
H	-0.851490	6.281302	1.792336
H	-0.775489	4.602557	-0.796446
H	-2.121615	4.714375	0.348376
H	-0.710389	3.678998	2.132826
H	-2.762256	2.645189	1.223936
H	-4.533191	1.364373	1.281000
H	-4.925917	-2.250290	-1.000596
H	-7.170044	-1.847662	-0.915677
H	-8.108184	-1.930903	0.590366
H	-6.566433	-2.804275	0.468320
H	-5.752689	-1.609246	2.604958
H	-7.364115	-0.844290	2.685669
H	-5.897962	0.160305	2.756329
H	-6.782800	1.556894	0.736369
H	-8.238601	0.537225	0.694563

H	-7.238905	0.725921	-0.772747
H	-0.379164	-2.417941	-1.342700
H	-0.853413	-3.824335	-2.297880
H	-1.509061	-3.582845	-0.653829
H	-3.800797	-4.013662	-1.635277
H	-3.062541	-4.150120	-3.243065
H	-4.411949	-3.024512	-2.994449
H	-2.989831	-1.176308	-3.983753
H	-1.720675	-2.399912	-4.257079
H	-1.329167	-0.871520	-3.442038
H	-0.571813	-0.722670	-1.513774
H	4.278821	-2.663859	1.646632
H	5.328122	-4.883429	2.070421
H	3.896420	-6.917258	2.386277
H	1.402420	-6.700808	2.268072
H	0.352691	-4.486985	1.835111
H	3.175793	2.382447	-1.281270
H	4.217101	2.104502	-2.706523
H	2.070052	2.525763	-3.786728
H	1.184174	1.672312	-2.494850
H	2.706768	0.449081	-4.826124
H	1.325072	-0.212027	-3.937030
H	4.230026	-0.657941	-3.477833
H	2.891708	-1.489755	-2.644351
H	4.441308	-1.173099	-0.784422
H	3.134624	-0.075090	0.734105
H	6.127573	0.258462	-1.994919
H	5.842476	1.552739	-0.819698
H	7.894984	0.219717	-0.206611
H	6.637211	0.049547	1.049767
H	6.916510	-1.267374	-0.115979

catalyst_alkyne_enamine.xyz

C	3.622636	-0.957249	-3.955583
C	3.711764	-0.100069	-2.817678
C	4.982022	0.040985	-2.180189
C	6.096307	-0.649716	-2.661174
C	5.991355	-1.492511	-3.781543
C	4.748289	-1.638660	-4.422085
C	2.579385	0.598077	-2.346246
C	1.545434	1.055552	-1.787187
Cu	0.583297	-0.274422	-0.541381
O	-1.146767	0.666776	-0.812037
C	-2.356309	0.248381	-0.613061
C	-2.663419	-1.012578	0.046212
C	-4.013735	-1.374960	0.301936
C	-5.096349	-0.585629	-0.070513
C	-4.778204	0.615076	-0.758932
C	-3.484680	1.054266	-1.054852

C	-1.671201	-1.978027	0.466554
N	-0.379435	-1.900667	0.325471
C	0.542732	-2.944863	0.765908
C	0.016142	-4.002030	1.751769
C	1.104944	-4.997825	2.176309
C	2.331060	-4.287587	2.760339
C	2.879013	-3.244004	1.781360
C	1.802097	-2.237731	1.344209
N	2.286534	-1.234949	0.385684
C	-3.250942	2.365259	-1.852409
C	-2.481450	2.046319	-3.161937
C	-6.547055	-1.026616	0.246274
C	-7.597414	-0.008750	-0.248748
C	-6.841562	-2.384364	-0.441629
C	-6.720048	-1.178083	1.779508
C	-4.571959	3.057478	-2.259655
C	-2.451897	3.382417	-0.996126
H	2.837237	-1.674457	-0.357289
H	2.904464	-0.548182	0.830197
H	1.458158	-1.677122	2.232971
H	3.272905	-3.754248	0.879904
H	3.730103	-2.695218	2.224539
H	3.118167	-5.019403	3.017121
H	2.050691	-3.788882	3.708297
H	0.689189	-5.714740	2.907417
H	1.414440	-5.598853	1.299274
H	-0.386201	-3.488068	2.645355
H	-0.827110	-4.553203	1.301068
H	0.879155	-3.476789	-0.151368
H	-2.111398	-2.873157	0.943797
H	-4.183871	-2.329379	0.813949
H	-5.606290	1.239555	-1.086943
H	-7.468248	0.980460	0.221827
H	-8.612066	-0.361812	0.003502
H	-7.561200	0.124617	-1.343099
H	-6.729910	-2.303003	-1.536680
H	-7.873023	-2.718770	-0.227846
H	-6.159596	-3.178985	-0.096621
H	-6.034110	-1.931851	2.200063
H	-7.749535	-1.492136	2.030661
H	-6.519974	-0.222541	2.294441
H	-1.514479	1.568569	-2.955434
H	-2.296458	2.972137	-3.736723
H	-3.070646	1.366182	-3.801779
H	-5.200138	2.418733	-2.903661
H	-4.345287	3.972388	-2.833500
H	-5.172258	3.363998	-1.386043
H	-3.009706	3.640909	-0.078919
H	-2.285572	4.316826	-1.562628

H	-1.475939	2.976912	-0.699956
H	0.880376	1.922785	-1.799549
H	5.069686	0.691949	-1.304554
H	7.062557	-0.528588	-2.156740
H	6.871299	-2.028997	-4.154183
H	4.657747	-2.291791	-5.298319
H	2.657399	-1.071517	-4.460051
N	1.926306	2.726763	1.959253
C	1.181385	1.795757	2.791926
C	-0.261121	2.244747	2.546677
C	-0.140173	3.783243	2.483947
C	1.345393	4.054691	2.124191
H	1.367558	0.756884	2.476092
H	1.437717	1.868623	3.876730
H	-0.958808	1.897945	3.327651
H	-0.602866	1.839602	1.580695
H	-0.383671	4.239006	3.459083
H	-0.831517	4.217190	1.743856
H	1.834198	4.641764	2.936902
H	1.464216	4.642896	1.193823
C	3.350083	2.589812	1.762954
H	3.677503	3.532706	1.271238
C	4.198878	2.401367	3.031937
O	3.627547	1.484042	0.900442
H	3.044605	1.589310	0.127824
C	5.708321	2.454585	2.790916
H	3.901552	3.186682	3.750756
H	3.925997	1.435282	3.493311
H	6.270294	2.332938	3.733942
H	6.026692	1.657493	2.098540
H	6.011943	3.420433	2.346729

catalyst_product.xyz

C	2.292075	2.846671	0.444428
C	0.813763	3.148349	0.775369
C	0.458166	4.566186	0.283807
C	1.391497	5.623129	0.891826
C	2.870347	5.305268	0.632941
C	3.229115	3.881892	1.082912
N	-0.021105	2.078665	0.219340
C	-1.279715	2.039343	0.558558
C	-2.259329	1.022283	0.236229
C	-1.926820	-0.251756	-0.394994
C	-3.016529	-1.215996	-0.523013
C	-4.301930	-0.845430	-0.112336
C	-4.644375	0.404147	0.469597
C	-3.591818	1.300215	0.642365
O	-0.738998	-0.529437	-0.813667
Cu	1.060939	0.316255	-0.257523

N	2.585838	1.444242	0.779158
C	-2.751700	-2.624754	-1.117863
C	-2.273036	-2.495454	-2.587413
C	-6.082635	0.774594	0.910779
C	-6.557355	2.028728	0.132215
C	2.133782	-1.572612	-0.318120
C	2.469208	-1.993702	0.810971
C	2.747545	-2.284446	2.168587
C	1.803286	-2.980876	2.979488
C	2.084767	-3.264002	4.318786
C	3.305388	-2.868305	4.894294
C	4.248289	-2.182190	4.108583
C	3.981488	-1.893680	2.767310
N	2.133183	-0.331010	-2.378955
C	-1.681798	-3.359734	-0.267548
C	-4.012166	-3.520364	-1.130085
C	-7.096674	-0.359297	0.644299
C	-6.100884	1.079872	2.431160
H	2.525116	1.292269	1.790576
H	3.543545	1.198471	0.522542
H	2.392329	2.916776	-0.653492
H	3.156798	3.801656	2.184877
H	4.275855	3.644221	0.817670
H	3.517791	6.040440	1.144067
H	3.081208	5.405440	-0.449221
H	1.138975	6.621354	0.490358
H	1.216205	5.674312	1.983738
H	0.526874	4.582486	-0.820302
H	-0.590502	4.797758	0.536657
H	0.718817	3.143100	1.883895
H	-1.680721	2.850444	1.201409
H	-3.777117	2.275397	1.107993
H	-5.100305	-1.573005	-0.242572
H	-7.163909	-0.610670	-0.427738
H	-8.103570	-0.047796	0.971747
H	-6.843561	-1.280192	1.196457
H	-5.769130	0.202334	3.013200
H	-7.119998	1.344714	2.766863
H	-5.438926	1.922400	2.691297
H	-5.905247	2.898601	0.316400
H	-7.582486	2.313754	0.431270
H	-6.561754	1.838701	-0.955324
H	-0.746616	-2.787213	-0.219027
H	-1.461106	-4.354238	-0.696713
H	-2.046625	-3.514881	0.763606
H	-4.416519	-3.689705	-0.117441
H	-3.754995	-4.509273	-1.547311
H	-4.819154	-3.104984	-1.757410
H	-3.052541	-2.020997	-3.209638

H	-2.060156	-3.491326	-3.017717
H	-1.362941	-1.886078	-2.655829
H	4.721790	-1.359705	2.161651
H	5.203362	-1.869874	4.548018
H	3.519934	-3.093006	5.945283
H	1.342562	-3.798549	4.923838
H	0.848758	-3.288613	2.539524
C	3.473898	0.216547	-2.610277
C	3.223409	1.515347	-3.411375
C	1.746794	1.419939	-3.871727
C	1.360984	-0.034661	-3.585920
H	3.994325	0.377250	-1.651702
H	4.105817	-0.471816	-3.208830
H	3.915770	1.576302	-4.267598
H	3.396764	2.415659	-2.800221
H	1.606588	1.691520	-4.931115
H	1.110545	2.088117	-3.267382
H	1.639448	-0.692853	-4.441089
H	0.286557	-0.166352	-3.385318
C	2.010034	-1.689632	-1.801784
H	0.961505	-1.977084	-1.983971
C	2.911753	-2.786587	-2.421571
C	2.498859	-4.210472	-2.045393
H	2.866573	-2.671747	-3.519994
H	3.961596	-2.611668	-2.128464
H	3.155028	-4.952463	-2.533738
H	2.559752	-4.374342	-0.956177
H	1.460944	-4.425576	-2.357407

Synthesis and Characterization data for propargylamines

Standard procedure for synthesis of propargylamines:
Aldehyde (1 mmol, 1 eq.), amine (1.1 mmol, 1.1 eq.), alkyne (1.2 mmol, 1.2 eq.) and crushed 4Å molecular sieves (100 mg) were combined in dry DCM (2 mL) under an argon. **(1)**, (17 mg, 2 mol%) was added and the reaction was stirred at R.T under Ar for 16 hours. The reaction mixture was filtered over celite and purified by flash column chromatography (Hex:EtOAc - 0% EtOAc to 50%) to afford pure propargylamines.

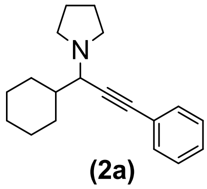

(2a)

1-(1-cyclohexyl-3-phenylprop-2-yn-1-yl)pyrrolidine, **(2a)**, was synthesised using the standard procedure. Yellow oil (238 mg, 0. 89 mmol 89%); ^{1}H NMR (600 MHz, $CDCl_3$) δ 7.47 – 7.40 (m, 2H), 7.33 – 7.24 (m, 3H), 3.35 (d, *J* = 8.4 Hz, 1H), 2.78 – 2.70 (m, 2H), 2.69 – 2.60 (m, 2H), 2.13 – 2.07 (m, 1H), 1.99 – 1.92 (m, 1H), 1.79 (td, *J* = 10.7, 8.5, 5.2 Hz, 6H), 1.71 – 1.65 (m, 1H), 1.58 (tdt, *J* = 11.6, 8.4, 3.4 Hz, 1H), 1.33 – 1.05 (m, 5H).
;^{13}C NMR (151 MHz, $cdcl_3$) δ 131.7, 128.2, 127.7, 123.6, 87.9, 85.7, 61.3, 41.3, 30.7, 30.3, 26.7, 26.2 (d, *J* = 3.2 Hz), 23.5; (HRMS + pTOF-ES) cald $C_{19}H_{26}N$ = $[M + H]^{+}$: 268.2065, observed: 268.2057.

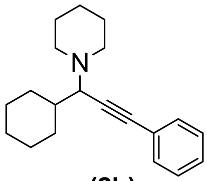

(2b)

1-(1-cyclohexyl-3-phenylprop-2-yn-1-yl)piperidine, **(2b)**, was synthesised using the standard procedure. Yellow oil (217 mg, 0.77 mmol, 77%); ^{1}H NMR (600 MHz, $CDCl_3$) δ 7.47 – 7.42 (m, 2H), 7.33 – 7.24 (m, 3H), 3.11 (d, *J* = 9.9 Hz, 1H), 2.67 – 2.60 (m, 2H), 2.44 – 2.37 (m, 2H), 2.11 (dtd, *J* = 11.1, 3.7, 1.9 Hz, 1H), 2.04 (ddt, *J* = 12.1, 3.9, 2.0 Hz, 1H), 1.82 – 1.72 (m, 2H), 1.72 – 1.52 (m, 6H), 1.48 – 1.40 (m, 2H), 1.34 – 1.12 (m, 3H), 1.10 – 0.85 (m, 2H); ^{13}C NMR (151 MHz, $CDCl_3$) δ 131.7, 128.2, 127.6, 123.8, 87.8, 86.1, 64.4, 39.5, 31.3, 30.4, 26.8, 26.3 (d, *J* = 2.7 Hz), 26.1, 24.7; (HRMS + pTOF-ES) cald $C_{20}H_{28}N$ = $[M + H]^{+}$: 282.2222, observed: 282.2235.

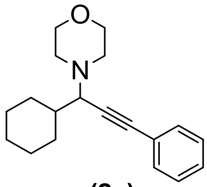

(2c)

4-(1-cyclohexyl-3-phenylprop-2-yn-1-yl)morpholine, **(2c)**, was synthesised using the standard procedure. Yellow oil (254 mg, 0.90 mmol, 90%); ^{1}H NMR (600 MHz, $CDCl_3$) δ 7.47 – 7.41 (m, 2H), 7.34 – 7.28 (m, 3H), 3.80 – 3.69 (m, 4H), 3.13 (d, *J* = 9.8 Hz, 1H), 2.70 (ddd, *J* = 10.6, 5.9, 3.1 Hz, 2H), 2.51 (ddd, *J* = 11.4, 6.2, 3.2 Hz, 2H), 2.14 – 2.01 (m, 2H), 1.89 – 1.54 (m, 4H), 1.36 – 1.12 (m,

3H), 1.10 – 0.91 (m, 2H); ^{13}C NMR (151 MHz, $CDCl_3$) δ 131.7, 128.2, 127.8, 123.4, 86.7, 86.6, 67.2, 63.9, 39.0, 31.0, 30.3, 26.7, 26.2; (HRMS + pTOF-ES) cald $C_{19}H_{26}NO$ = $[M + H]^+$: 284.2014, observed: 284.1014

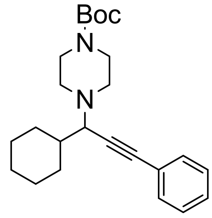

(2d)

tert-butyl 4-(1-cyclohexyl-3-phenylprop-2-yn-1-yl)piperazine-1-carboxylate, **(2d)**, was synthesised using the standard procedure. Colourless oil (319 mg, 0.83 mmol, 83%); ^{1}H NMR (600 MHz, $CDCl_3$) δ 7.42 (dd, *J* = 6.7, 3.0 Hz, 2H), 7.29 (dd, *J* = 5.1, 1.9 Hz, 3H), 3.42 (s, 4H), 3.16 (d, *J* = 10.0 Hz, 1H), 2.63 (s, 2H), 2.43 (s, 2H), 2.13 – 2.07 (m, 1H), 2.04 (d, *J* = 13.3 Hz, 1H), 1.81 – 1.71 (m, 2H), 1.70 – 1.66 (m, 1H), 1.65 – 1.55 (m, 2H), 1.46 (s, 9H), 1.27 – 1.17 (m, 2H), 1.08 – 0.90 (m, 2H); ^{13}C NMR (151 MHz, $CDCl_3$) δ 154.9, 131.7, 128.2, 127.8, 123.3, 86.7, 86.5, 79.5, 63.7, 39.4, 31.1, 30.3, 28.4, 26.7, 26.1, 26.0; (HRMS + pTOF-ES) cald $C_{24}H_{35}N_2O_2$ = $[M + H]^+$: 383.2699, observed: 383.2707 .

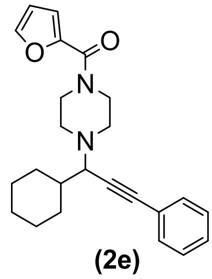

(2e)

(4-(1-cyclohexyl-3-phenylprop-2-yn-1-yl)piperazin-1-yl)(furan-2-yl)methanone, **(2e)**, was synthesised using the standard procedure. Colourless oil (307 mg, 0.82 mmol, 82%); ^{1}H NMR (600 MHz, $CDCl_3$) δ 7.47 – 7.44 (m, 1H), 7.40 (dd, *J* = 6.6, 3.1 Hz, 2H), 7.27 (qd, *J* = 3.6, 1.2 Hz, 3H), 6.97 (d, *J* = 3.4 Hz, 1H), 6.45 (dd, *J* = 3.4, 1.8 Hz, 1H), 3.84 (s, 4H), 3.19 (d, *J* = 10.0 Hz, 1H), 2.77 – 2.71 (m, 2H), 2.57 – 2.50 (m, 2H), 2.14 – 2.03 (m, 2H), 1.81 – 1.73 (m, 2H), 1.71 – 1.67 (m, 1H), 1.65 – 1.55 (m, 1H), 1.33 – 1.14 (m, 3H), 1.08 – 0.91 (m, 2H); ^{13}C NMR (151 MHz, $CDCl_3$) δ 159.0, 147.9, 143.6, 131.7, 128.2, 127.9, 123.1, 116.2, 111.2, 86.9, 86.1, 63.6, 39.4, 31.1, 30.4, 26.7, 26.1, 26.0; (HRMS + pTOF-ES) cald $C_{24}H_{29}N_2O_2$ = $[M + H]^+$: 377.2229, observed: 377.2239

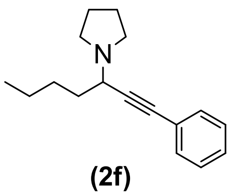

(2f)

1-(1-phenylhept-1-yn-3-yl)pyrrolidine, **(2f)**, was synthesised using the standard procedure. Yellow oil (228 mg, 0.94 mmol, 94%); ^{1}H NMR (600 MHz, $CDCl_3$) δ 7.46 – 7.40 (m, 2H), 7.33 – 7.25 (m, 3H), 3.67 (dd, *J* = 9.1, 5.8 Hz, 1H), 2.76 (qd, *J* = 6.6, 2.9 Hz, 2H), 2.68 (tt, *J* = 6.1, 3.9 Hz, 2H), 1.80 (td, *J* = 6.1, 3.3 Hz, 4H), 1.76 – 1.66 (m, 2H), 1.62 – 1.53 (m, 1H), 1.50 – 1.33 (m, 3H), 0.93 (t, *J* = 7.3 Hz,

3H); ^{13}C NMR (151 MHz, $CDCl_3$) δ 131.9, 128.3, 127.9, 123.6, 88.5, 85.3, 55.2, 49.9, 35.0, 29.1, 23.6, 22.7, 14.2; (HRMS + pTOF-ES) cald $C_{17}H_{24}N$ = $[M + H]^+$: 242.1909, observed: 242.1913

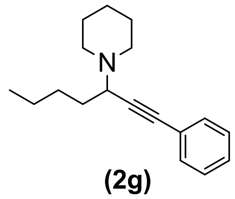

(2g)

1-(1-phenylhept-1-yn-3-yl)piperidine, **(2g)**, was synthesised using the standard procedure. Yellow oil (208 mg, 0.81 mmol, 81%); ^1H NMR (600 MHz, $CDCl_3$) δ 7.47 – 7.41 (m, 2H), 7.33 – 7.27 (m, 3H), 3.48 (dd, *J* = 9.6, 5.4 Hz, 1H), 2.72 – 2.65 (m, 2H), 2.52 – 2.45 (m, 2H), 1.80 – 1.50 (m, 7H), 1.50 – 1.32 (m, 5H), 0.93 (t, *J* = 7.2 Hz, 3H); ^{13}C NMR (151 MHz, $CDCl_3$) δ 131.7, 128.2, 127.7, 123.6, 88.2, 85.6, 58.6, 33.3, 29.2, 26.2, 24.6, 22.5, 14.1; (HRMS + pTOF-ES) cald $C_{18}H_{26}N$ = $[M + H]^+$: 256.2065, observed: 256.2075

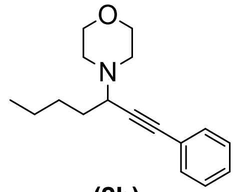

(2h)

4-(1-phenylhept-1-yn-3-yl)morpholine, **(2h)**, was synthesised using the standard procedure. Yellow oil (200 mg, 0.78 mmol 78%); ^1H NMR (600 MHz, $CDCl_3$) δ 7.43 (dd, *J* = 6.6, 3.0 Hz, 2H), 7.30 (dt, *J* = 4.7, 2.9 Hz, 3H), 3.81 – 3.71 (m, 4H), 3.49 (dd, *J* = 8.7, 6.4 Hz, 1H), 2.75 (ddd, *J* = 11.3, 6.2, 3.2 Hz, 2H), 2.57 (ddd, *J* = 11.4, 6.1, 3.2 Hz, 2H), 1.77 – 1.67 (m, 2H), 1.60 – 1.28 (m, 4H), 0.93 (t, *J* = 7.3 Hz, 3H); ^{13}C NMR (151 MHz, $CDCl_3$) δ 131.7, 128.2, 127.9, 123.2, 87.2, 86.1, 67.2, 58.1, 32.7, 28.8, 22.5, 14.1; (HRMS + pTOF-ES) cald $C_{17}H_{22}NO$ = $[M + H]^+$: 256.1701, observed: 256.2075

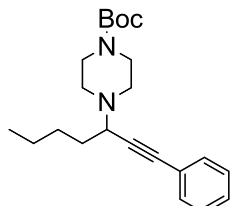

(2i)

tert-butyl 4-(1-phenylhept-1-yn-3-yl)piperazine-1-carboxylate, **(2i)**, was synthesised using the standard procedure. Colourless oil (241 mg, 0.68 mmol, 68%); ^1H NMR (600 MHz, $CDCl_3$) δ 7.41 (dt, *J* = 5.9, 3.5 Hz, 2H), 7.33 – 7.27 (m, 3H), 3.53 (dd, *J* = 8.8, 6.2 Hz, 1H), 3.51 – 3.36 (m, 4H), 2.68 (s, 2H), 2.49 (s, 2H), 1.76 – 1.67 (m, 2H), 1.60 – 1.42 (m, 2H), 1.46 (s, 9H), 1.39 – 1.35 (m, 2H), 0.93 (t, *J* = 7.3 Hz, 3H); ^{13}C NMR (151 MHz, $CDCl_3$) δ 154.9, 131.9, 128.4, 128.1, 123.3, 87.2, 86.2, 79.7, 58.1, 49.3, 33.1, 29.0, 28.6, 22.6, 14.2; (HRMS + pTOF-ES) cald $C_{22}H_{33}N_2O_2$ = $[M + H]^+$: 357.2542, observed: 357.2553

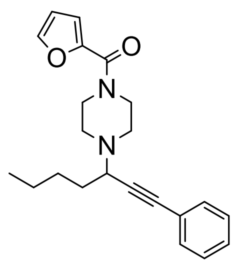

(2j)

furan-2-yl(4-(1-phenylhept-1-yn-3-yl)piperazin-1-yl)methanone, **(2j)**, was synthesised using the standard procedure. Colourless oil (229 mg, 0.65 mmol 65%); ^{1}H NMR (600 MHz, $CDCl_3$) δ 7.47 (d, *J* = 1.8 Hz, 1H), 7.44 – 7.38 (m, 2H), 7.33 – 7.26 (m, 3H), 6.99 (d, *J* = 3.4 Hz, 1H), 6.47 (dd, *J* = 3.5, 1.8 Hz, 1H), 3.87 (s, 4H), 3.56 (dd, *J* = 8.5, 6.5 Hz, 1H), 2.79 (dt, *J* = 10.9, 4.6 Hz, 2H), 2.61 (dt, *J* = 11.7, 4.5 Hz, 2H), 1.79 – 1.69 (m, 2H), 1.65 – 1.62 (m, 1H), 1.59 – 1.45 (m, 1H), 1.48 – 1.34 (m, 1H), 0.94 (t, *J* = 7.3 Hz, 3H); ^{13}C NMR (151 MHz, $CDCl_3$) δ 159.0, 147.9, 143.6, 131.7, 128.2, 128.0, 123.0, 116.3, 111.2, 86.7, 86.2, 57.8, 33.0, 28.8, 22.5, 14.1; (HRMS + pTOF-ES) cald $C_{22}H_{27}N_2O_2$ = $[M + H]^+$: 351.2073, observed: 351.2075

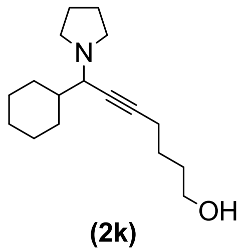

(2k)

7-cyclohexyl-7-(pyrrolidin-1-yl)hept-5-yn-1-ol, **(2k)**, was synthesised using the standard procedure. Yellow oil (240 mg, 0.91 mmol, 91%): ^{1}H NMR (600 MHz, $CDCl_3$) δ 3.67 (t, *J* = 6.4 Hz, 2H), 3.05 (dt, *J* = 8.1, 2.1 Hz, 1H), 2.62 (tq, *J* = 6.0, 3.6, 3.1 Hz, 2H), 2.52 (qd, *J* = 6.2, 2.8 Hz, 2H), 2.25 (td, *J* = 7.0, 2.1 Hz, 2H), 1.99 – 1.93 (m, 1H), 1.87 – 1.79 (m, 1H), 1.79 – 1.69 (m, 6H), 1.70 – 1.53 (m, 5H), 1.49 – 1.38 (m, 1H), 1.27 – 1.09 (m, 3H), 1.04 (dddt, *J* = 18.6, 12.4, 10.0, 3.5 Hz, 2H); ^{13}C NMR (151 MHz, $CDCl_3$) δ 85.1, 78.1, 62.4, 61.0, 50.1, 41.2, 31.9, 30.7, 29.8, 26.7, 26.2 (d, *J* = 8.2 Hz), 23.4, 18.5; (HRMS + pTOF-ES) cald $C_{17}H_{30}NO$ = $[M + H]^+$: 264.2327, observed: 264.2334

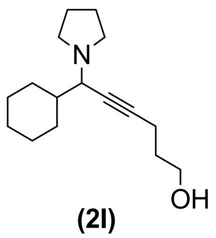

(2l)

6-cyclohexyl-6-(pyrrolidin-1-yl)hex-4-yn-1-ol, **(2l)**, was synthesised using the standard procedure. Yellow oil (207 mg, 0.83 mmol, 83%); ^{1}H NMR (600 MHz, $CDCl_3$) δ 3.77 (t, *J* = 6.2 Hz, 2H), 3.06 (d, *J* = 8.0, 2.2 Hz, 1H), 2.66 – 2.58 (m, 2H), 2.56 – 2.48 (m, 2H), 2.34 (td, *J* = 6.9, 2.1 Hz, 2H), 2.00 – 1.93 (m, 1H), 1.78 – 1.72 (m, 9H), 1.68 – 1.62 (m, 1H), 1.44 (dtd, *J* = 14.8, 7.4, 6.8, 3.7 Hz, 1H), 1.28 – 1.09 (m, 3H), 1.09 – 0.98 (m, 2H); ^{13}C NMR (151 MHz, $CDCl_3$) δ 84.7, 78.6, 62.2, 61.1, 50.3, 41.4, 31.9, 30.8, 30.0, 26.8, 26.3 (d, *J* = 7.5 Hz), 23.5, 15.5; (HRMS + pTOF-ES) cald $C_{16}H_{28}NO$ = $[M + H]^+$: 250.2171, observed: 250.2160

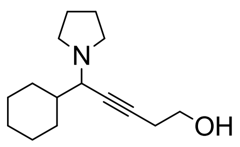

(2m)

5-cyclohexyl-5-(pyrrolidin-1-yl)pent-3-yn-1-ol, **(2m)**, was synthesised using the standard procedure. Yellow oil (211 mg, 0.90 mmol, 90%); ^{1}H NMR (600 MHz, $CDCl_3$) δ 3.68 (t, *J* = 6.4 Hz, 2H), 3.03 (d, *J* = 7.8, 2.1 Hz, 1H), 2.65 – 2.58 (m, 2H), 2.49 (dtd, *J* = 25.1, 6.4, 5.6, 2.3 Hz, 5H), 1.93 (ddt, *J* = 12.6, 3.6, 1.8 Hz, 1H), 1.80 (ddt, *J* = 12.6, 3.7, 2.0 Hz, 1H), 1.75 – 1.72 (m, 5H), 1.67 – 1.61 (m, 1H), 1.50 – 1.40 (m, 1H), 1.26 – 0.99 (m, 5H); ^{13}C NMR (151 MHz, $CDCl_3$) δ 81.9, 80.0, 61.4, 61.3, 50.5, 41.2, 30.8, 29.7, 26.3 (d, *J* = 11.5 Hz), 23.4, 23.3; (HRMS + pTOF-ES) cald $C_{15}H_{26}NO$ = [M + H]$^+$: 236.2014, observed: 236.2004

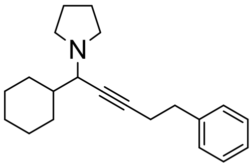

(2n)

1-(1-cyclohexyl-5-phenylpent-2-yn-1-yl)pyrrolidine, **(2n)**, was synthesised using the standard procedure. Yellow oil (262 mg, 0.89 mmol, 89%); ^{1}H NMR (600 MHz, $CDCl_3$) δ 7.28 (t, *J* = 7.5 Hz, 2H), 7.24 – 7.18 (m, 3H), 3.04 (d, *J* = 8.2, 2.2 Hz, 1H), 2.83 (t, *J* = 7.4 Hz, 2H), 2.61 – 2.51 (m, 4H), 2.51 – 2.43 (m, 2H), 1.96 – 1.88 (m, 1H), 1.85 – 1.79 (m, 1H), 1.73 – 1.70 (m, 6H), 1.67 – 1.62 (m, 1H), 1.45 – 1.36 (m, 1H), 1.27 – 1.08 (m, 3H), 1.04 – 0.94 (m, 2H); ^{13}C NMR (151 MHz, $CDCl_3$) δ 140.8, 128.5, 128.2, 126.1, 84.6, 78.5, 60.9, 49.9, 41.2, 35.5, 30.6, 29.9, 26.7, 26.2 (d, *J* = 5.8 Hz), 23.4, 20.8; (HRMS + pTOF-ES) cald $C_{21}H_{30}N$ = [M + H]$^+$: 296.2378, observed: 296.2395

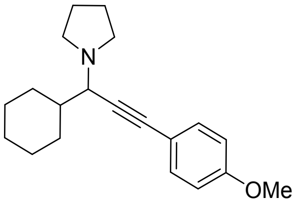

(2o)

1-(1-cyclohexyl-3-(4-methoxyphenyl)prop-2-yn-1-yl)pyrrolidine, **(2o)**, was synthesised using the standard procedure. Yellow oil (251 mg, 0.84 mmol, 84%); ^{1}H NMR (600 MHz, $CDCl_3$) δ 7.37 (d, 2H), 6.82 (d, 2H), 3.32 (d, *J* = 8.3 Hz, 1H), 2.81 – 2.57 (m, 4H), 2.11 – 2.04 (m, 1H), 1.97 – 1.90 (m, 1H), 1.78 – 1.72 (m, 6H), 1.71 – 1.64 (m, 1H), 1.61 – 1.52 (m, 1H), 1.31 – 1.04 (m, 5H); ^{13}C NMR (151 MHz, $CDCl_3$) δ 159.2, 133.2, 115.9, 113.9, 86.4, 85.5, 61.4, 55.4, 50.2, 41.5, 30.8, 30.3, 26.8, 26.4 (d, *J* = 3.6 Hz), 23.6;(HRMS + pTOF-ES) cald $C_{20}H_{28}NO$ = [M + H]$^+$: 298.2171, observed: 298.2166

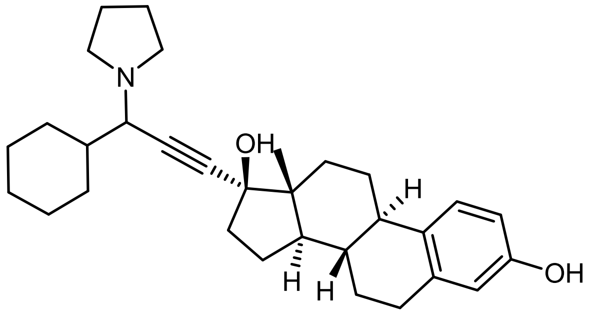

(**2p**)

(8S,9R,13R,14R,17R)-17-(3-cyclohexyl-3-(pyrrolidin-1-yl)prop-1-yn-1-yl)-13-methyl-7,8,9,11,12,13,14,15,16,17-decahydro-6H-cyclopenta[a]phenanthrene-3,17-diol, **(2p)**, was synthesised using the standard procedure at quarter scale. White solid (81 mg, 0.18 mmol, 70%); ^{1}H NMR (600 MHz, $CDCl_3$) δ 7.18 (dd, *J* = 8.5, 1.3 Hz, 1H), 6.66 – 6.62 (m, 1H), 6.56 (d, *J* = 2.7 Hz, 1H), 3.49 (s, 1H), 3.19 (dd, *J* = 8.0, 3.1 Hz, 1H), 2.87 – 2.78 (m, 2H), 2.68 (dq, *J* = 7.9, 5.4, 4.6 Hz, 2H), 2.60 (tt, *J* = 8.8, 4.6 Hz, 2H), 2.39 – 2.25 (m, 2H), 2.15 (td, *J* = 11.4, 4.2 Hz, 1H), 2.08 – 1.97 (m, 2H), 1.97 – 1.86 (m, 2H), 1.86 (s, 2H), 1.81 – 1.71 (m, 9H), 1.68 – 1.60 (m, 1H), 1.54 – 1.45 (m, 1H), 1.43 (s, 1H), 1.42 – 1.37 (m, 1H), 1.36 – 1.28 (m, 1H), 1.26 – 1.00 (m, 5H), 0.88 (s, 3H); ^{13}C NMR (151 MHz, $CDCl_3$) zδ 153.4, 138.3, 132.5, 126.6, 115.2, 112.7, 88.9, 84.2, 80.1, 61.0, 50.9, 50.3, 49.6, 47.2, 43.8, 41.2, 39.4, 32.9, 30.7, 30.1, 29.6, 27.3, 26.7, 26.4, 26.1, 23.5, 22.7, 12.8; (HRMS + pTOF-ES) cald $C_{31}H_{44}NO_2$ = $[M + H]^+$: 462.3372, observed: 462.3346

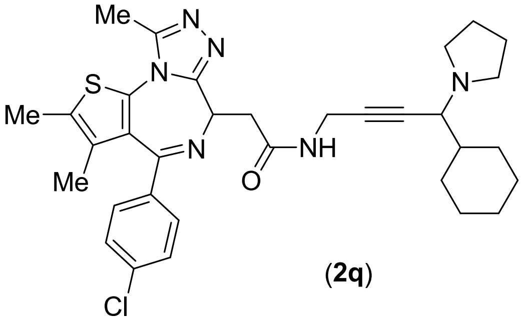

(**2q**)

(Rac)-2-(4-(4-Chlorophenyl)-2,3,9-trimethyl-6*H*-thieno[3,2-*f*][1,2,4]triazolo[4,3-*a*][1,4]diazepin-6-yl)-*N*-(4-cyclohexyl-4-(pyrrolidin-1-yl)but-2-yn-1-yl)acetamide (**2q**) was synthesised using the standard procedure at quarter scale and stirring for 40h.The resulting mixture was filtered through celite®, washing with dichloromethane (3 x 5 mL). The filtrate was concentrated under reduced pressure to give a green solid (48 mg). The resulting residue was purified by automated flash column chromatography (EtOAc/MeOH, 100:0 – 60:40, 12 g SiO_2) followed by automated reverse phase chromatography (H_2O (1% formic acid)/Acetonitrile (1% formic acid), 95:5 – 5:95, 12 g C28). The appropriate fractions were combined and concentrated to give 2-(4-(4-chlorophenyl)-2,3,9-trimethyl-6*H*-thieno[3,2-*f*][1,2,4]triazolo[4,3-*a*][1,4]diazepin-6-yl)-*N*-(4-cyclohexyl-4-(pyrrolidin-1-yl)but-2-yn-1-yl)acetamide as a pale yellow solid (16 mg, 53%). LCMS (UV, ESI) R_t = 10.11 min, $[M-H]^+$ *m/z* = 603.6, 98% purity. ^{1}H NMR (600 MHz, d_6–DMSO): δ = 8.61 (t, *J* = 5.3 Hz, 1H), 7.45 (d, *J* = 8.2 Hz, 2H), 7.40 (d, *J* = 8.2 Hz, 2H), 4.49 (t, *J* = 7.0 Hz, 1H), 4.01 – 3.89 (m, 2H), 3.29 – 3.17 (m, 2H), 3.15 (d, *J* = 9.0 Hz, 1H), 2.61 – 2.43 (m, 6H), 2.39 (s, 3H), 1.91 (d, *J* = 12.7 Hz, 1H), 1.83 (d, *J* = 13.0 Hz, 1H), 1.68 – 1.54 (m, 10H), 1.38 (m, 1H), 1.24 – 1.03 (m, 4H), 0.98 – 0.87 (m, 2H). ^{13}C NMR (151 MHz, d_6–DMSO): δ = 169.3, 163.1, 136.7, 135.2, 132.3, 130.8, 130.2, 129.8, 129.6, 128.5, 82.5, 79.1, 59.5, 55.0, 53.8, 49.1, 40.5, 37.4, 30.0, 29.9, 28.2, 26.2, 25.6, 23.0, 14.1, 12.7, 11.3. HRMS (ESI-[+H]) *m/z*: Calcd for $C_{33}H_{40}ClN_6OS$ 603.2673; Found 603.2694.

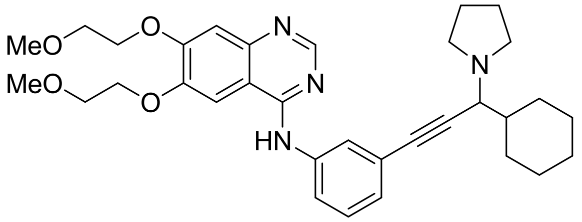

(**2r**)

N-(3-(3-Cyclohexyl-3-(pyrrolidin-1-yl)prop-1-yn-1-yl)phenyl)-6,7-bis(2-methoxyethoxy)quinazolin-4-amine (**2r**) was synthesised using the standard procedure at quarter scale and stirring for 40h. The resulting mixture was filtered through celite®, washing with dichloromethane (3 x 5 mL). The filtrate was concentrated under reduced pressure to give a green gum (177 mg). The resulting residue was purified by automated flash column chromatography (EtOAc/MeOH, 100:0 – 90:10, 12 g SiO_2) followed by automated reverse phase chromatography (H_2O (1% formic acid)/Acetonitrile (1% formic acid), 95:5 – 5:95, 12 g C28). The appropriate fractions were combined and concentrated to give *N*-(3-(3-cyclohexyl-3-(pyrrolidin-1-yl)prop-1-yn-1-yl)phenyl)-6,7-bis(2-methoxyethoxy)quinazolin-4-amine as a pale yellow solid (45 mg, 40%). LCMS (UV, ESI) R_t = 7.15 min, $[M-H]^+$ *m/z* = 559.6, 91% purity. ^1H NMR (600 MHz, d_6–DMSO): δ = 9.45 (s, 1H), 8.48 (s, 1H), 7.95 (ddd, *J* = 8.2, 2.1, 1.0 Hz, 1H), 7.86 (s, 1H), 7.78 (t, *J* = 1.9 Hz, 1H), 7.40 – 7.34 (m, 1H), 7.23 (s, 1H), 7.14 (dt, *J* = 7.6, 1.3 Hz, 1H), 4.32 – 4.26 (m, 4H), 3.81 – 3.76 (m, 2H), 3.76 – 3.72 (m, 2H), 3.37 (s, 3H), 3.35 (s, 3H), 2.67 – 2.61 (m, 2H), 2.61 – 2.55 (m, 2H), 2.03 (d, *J* = 12.9 Hz, 1H), 1.95 (d, *J* = 13.2 Hz, 1H), 1.76 – 1.67 (m, 6H), 1.63 (d, *J* = 11.8 Hz, 1H), 1.57 – 1.48 (m, 1H), 1.31 – 1.12 (m, 4H), 1.11 – 1.02 (m, 1H), 1.03 – 0.93 (m, 1H). ^{13}C NMR (151 MHz, d_6–DMSO): δ = 156.2, 153.7, 152.8, 148.1, 147.0, 139.8, 128.8, 126.2, 124.4, 122.8, 122.0, 108.9, 108.2, 103.1, 87.6, 85.6, 70.1, 68.4, 68.1, 59.8, 58.4, 49.1, 40.7, 40.1, 30.2, 26.3, 25.6, 23.1. HRMS (ESI-[+H]) *m/z*: Calcd for $C_{33}H_{43}N_4O_4$ 559.3284; Found 559.3301.

Characterisation

1-(1-cyclohexyl-3-phenylprop-2-yn-1-yl)pyrrolidine **(2a):**

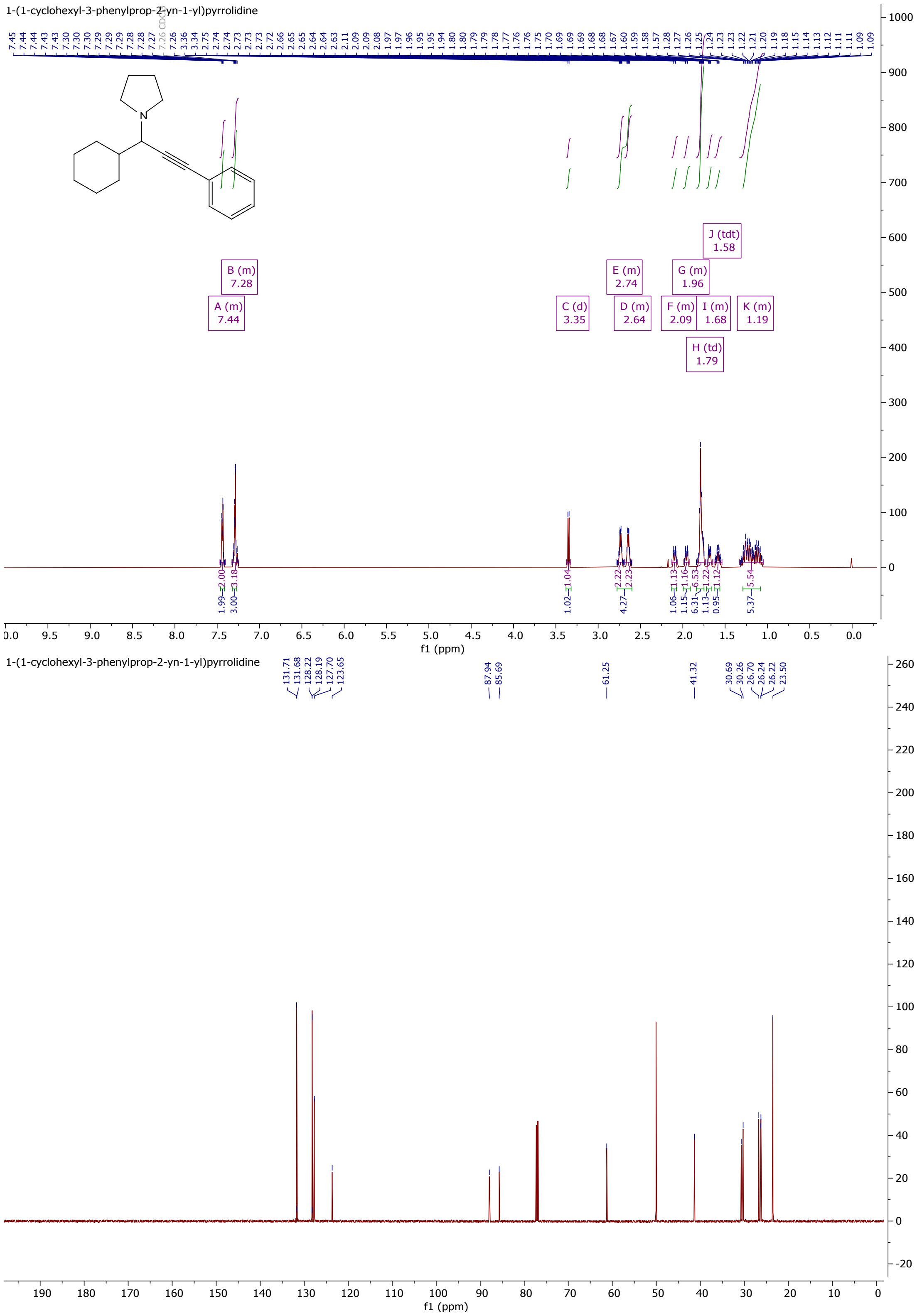

Figure S17: NMR spectra of (**2a**)

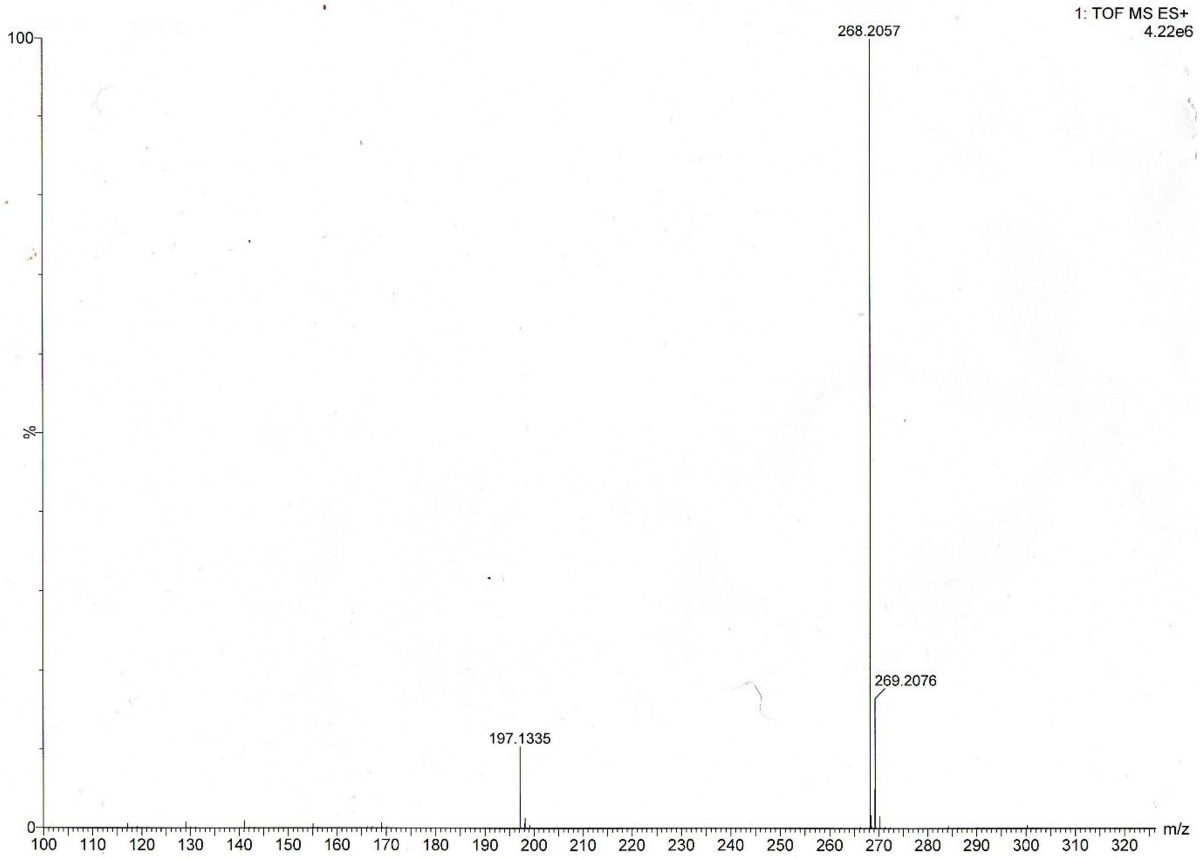

Figure S18: HRMS spectra of (**2a**)

1-(1-cyclohexyl-3-phenylprop-2-yn-1-yl)piperidine (**2b**):

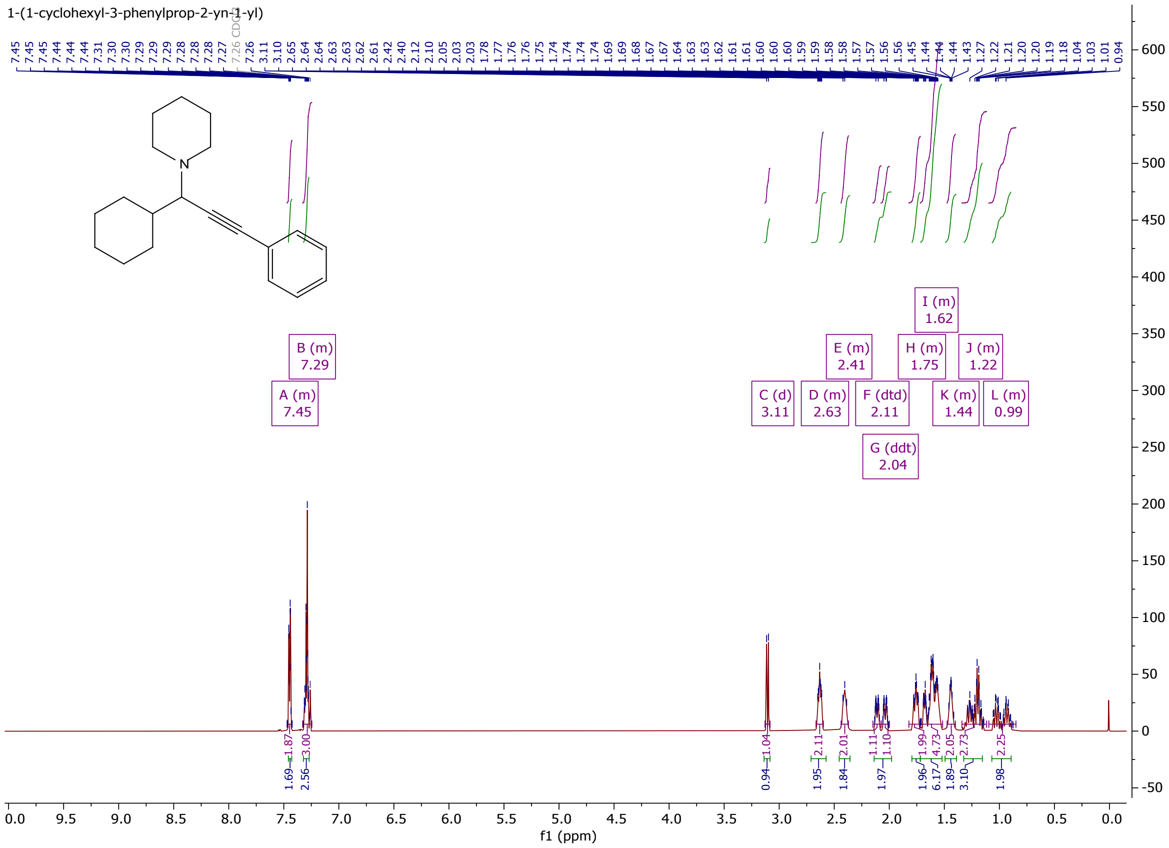

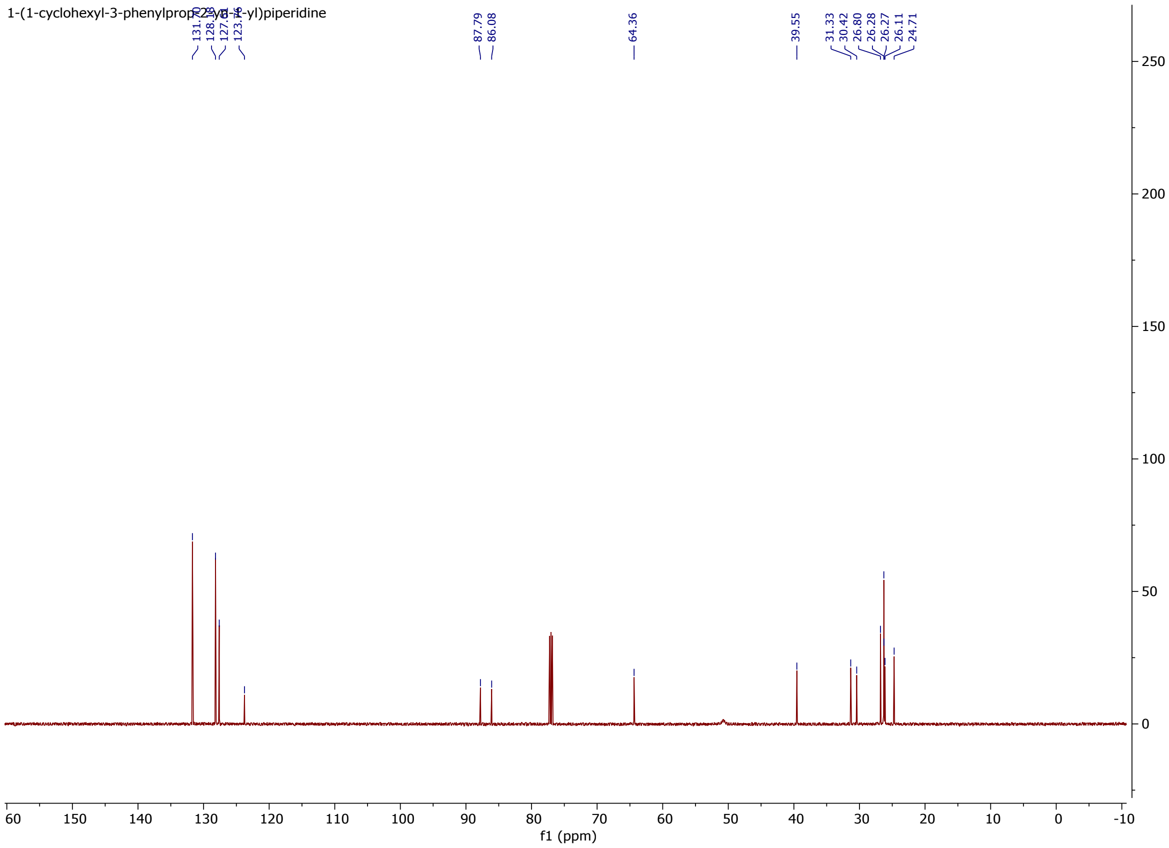

Figure S19: NMR spectra of (**2b**)

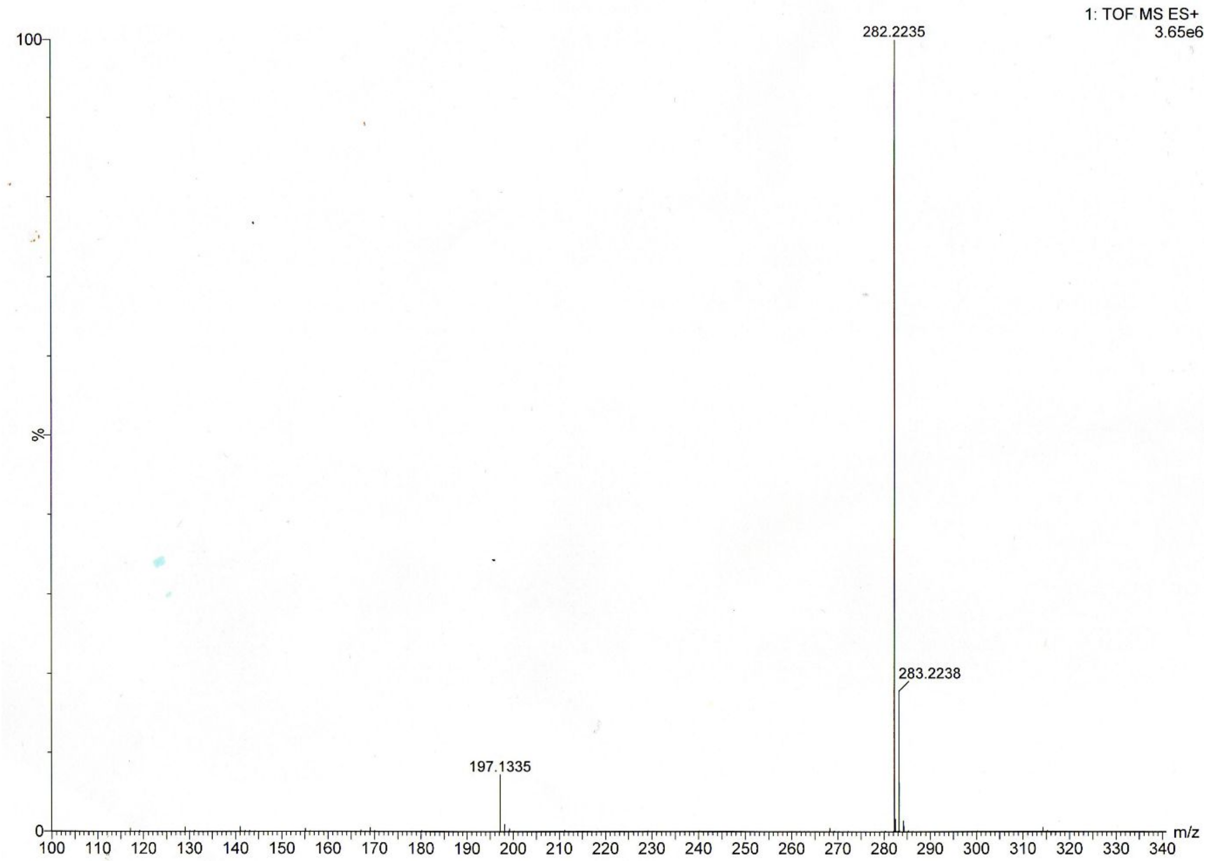

Figure S20: HRMS spectra of (**2b**)

4-(1-cyclohexyl-3-phenylprop-2-yn-1-yl)morpholine (**2c**):

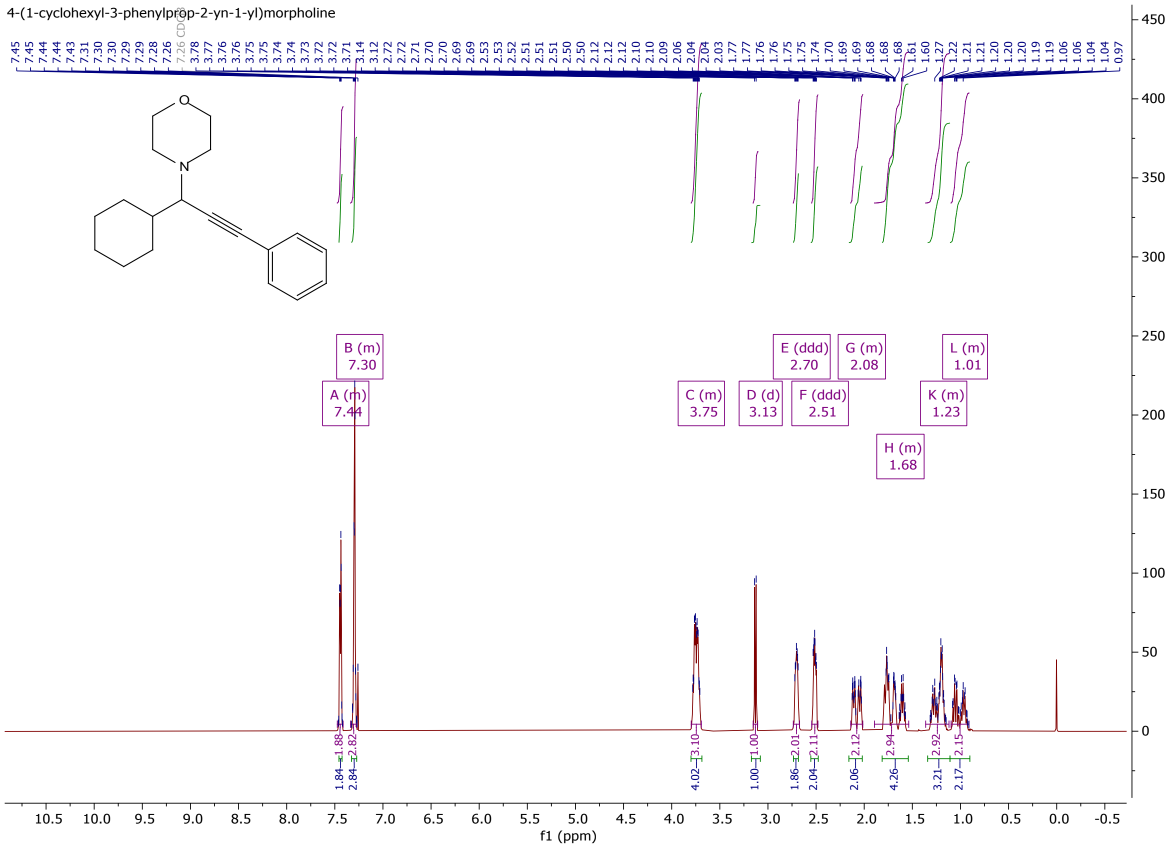

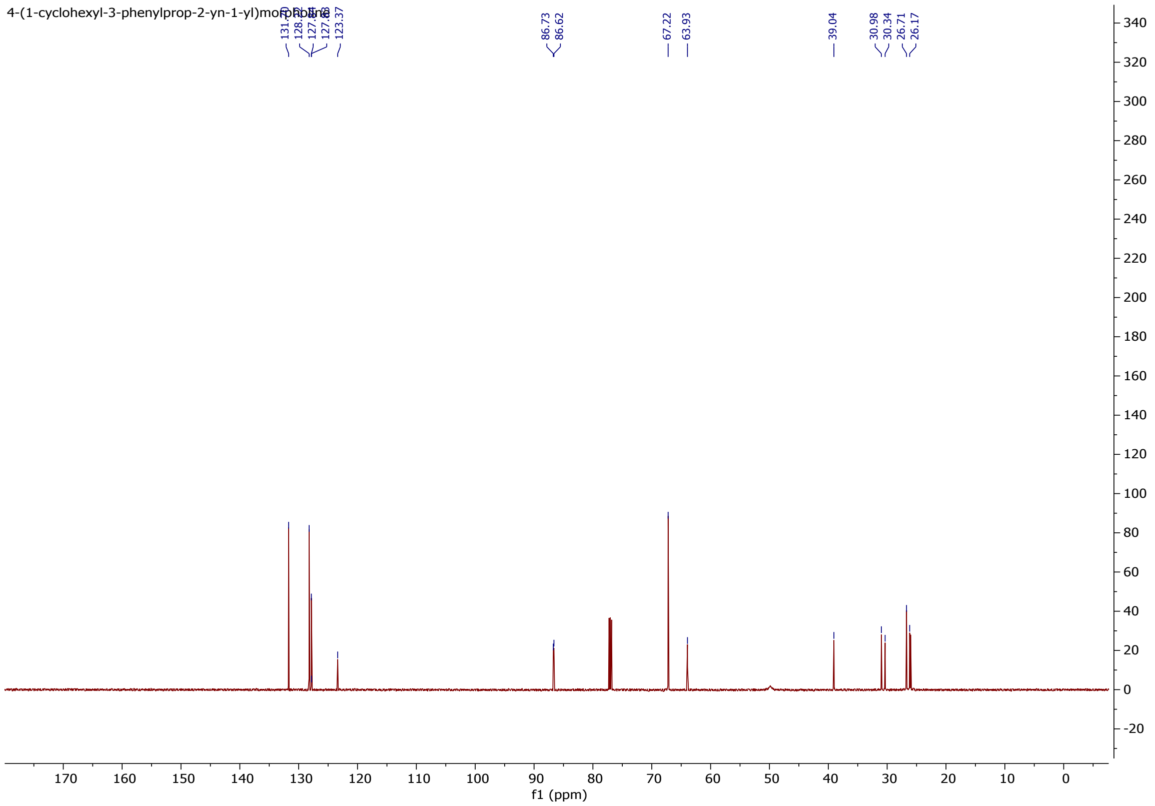

Figure S21: NMR spectra of (**2c**)

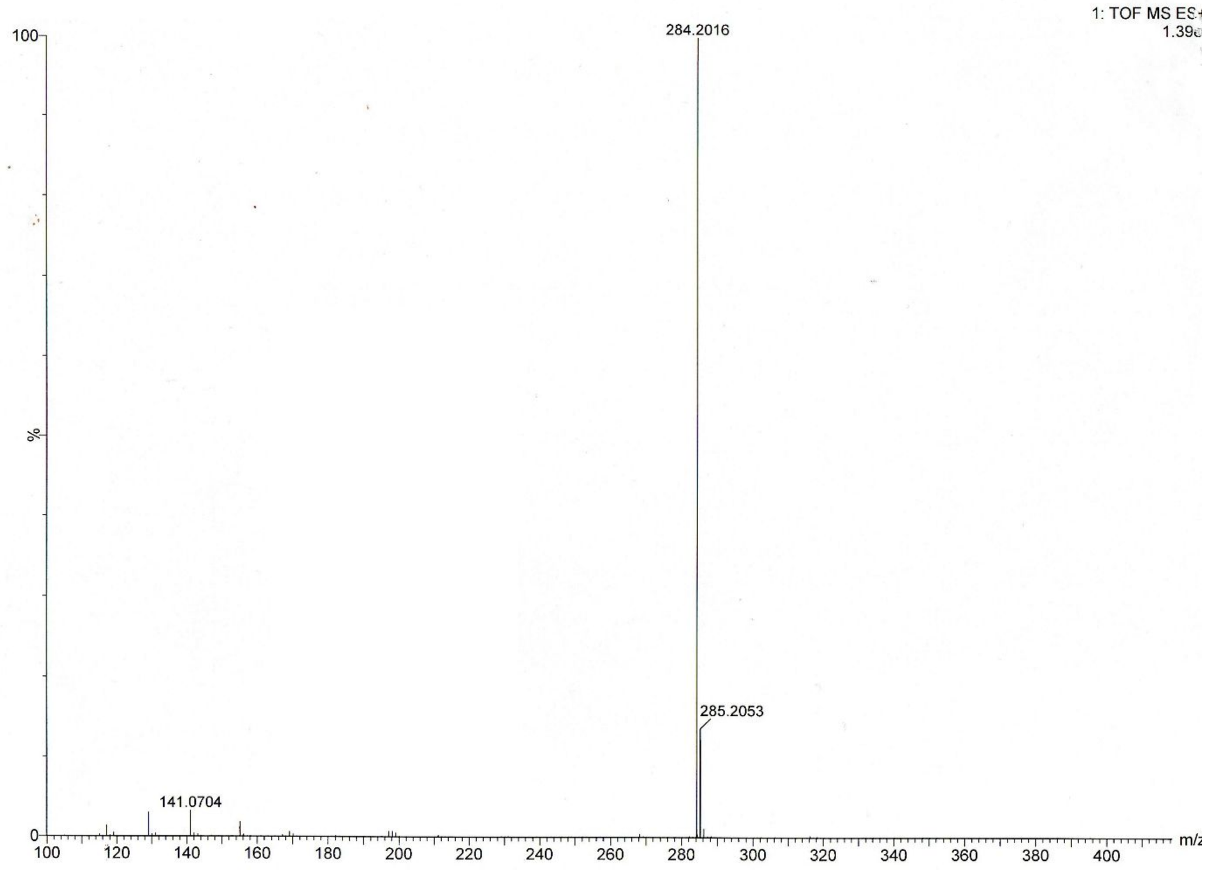

Figure S22: HRMS spectra of (**2c**)

tert-butyl 4-(1-cyclohexyl-3-phenylprop-2-yn-1-yl)piperazine-1-carboxylate (**2d**):

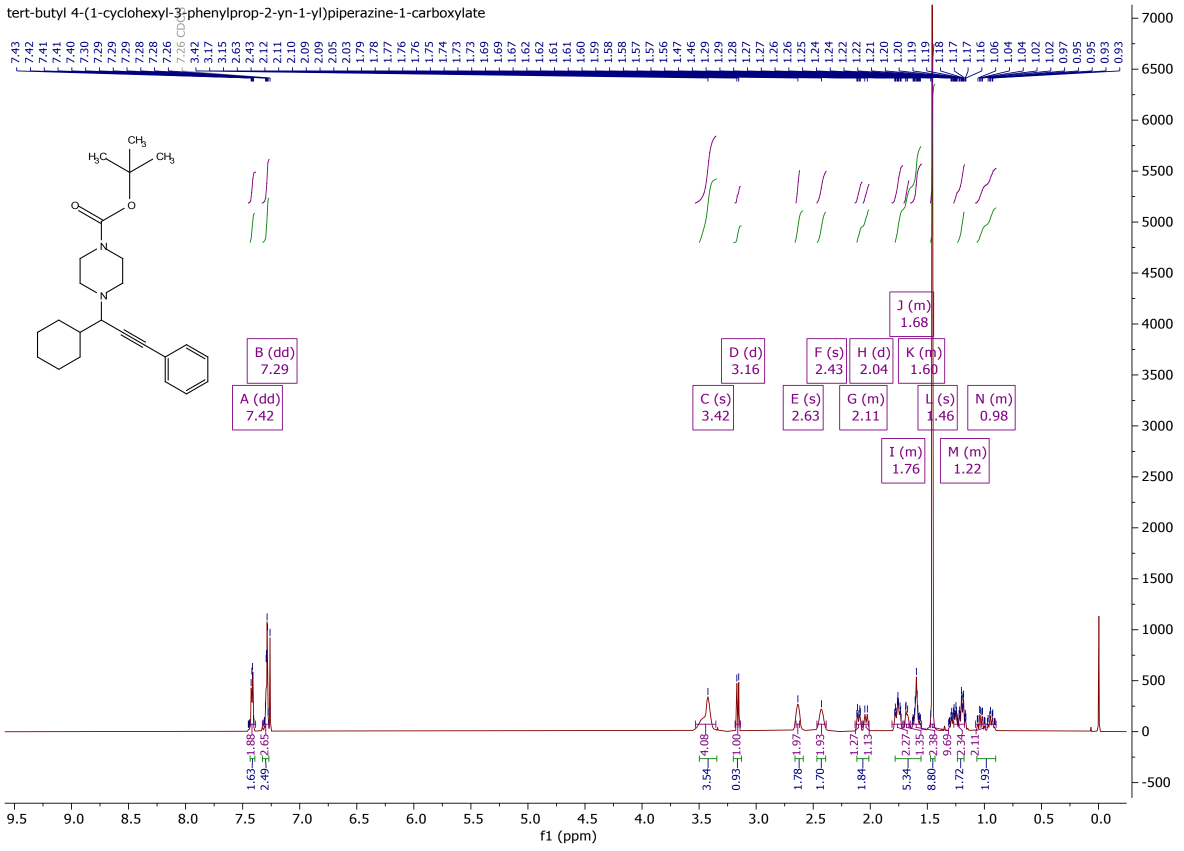

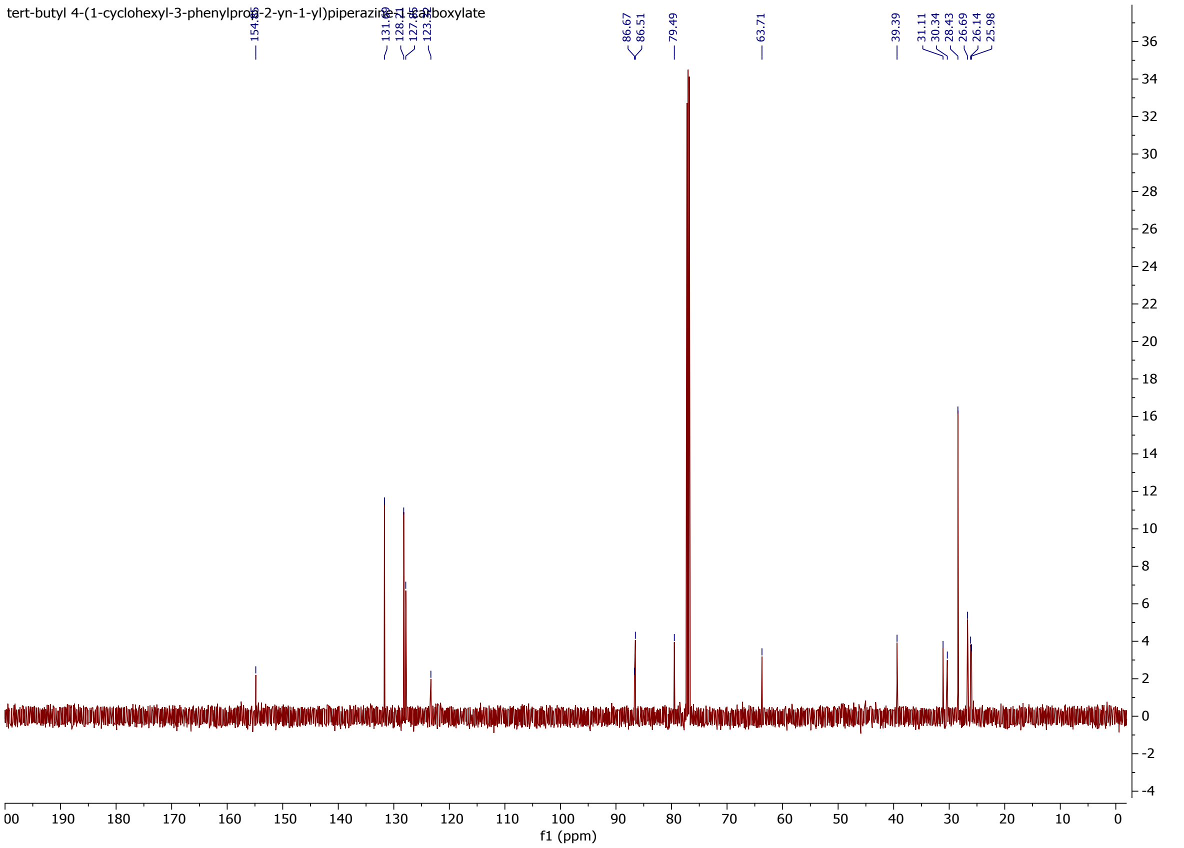

Figure S23: NMR spectra of (**2d**)

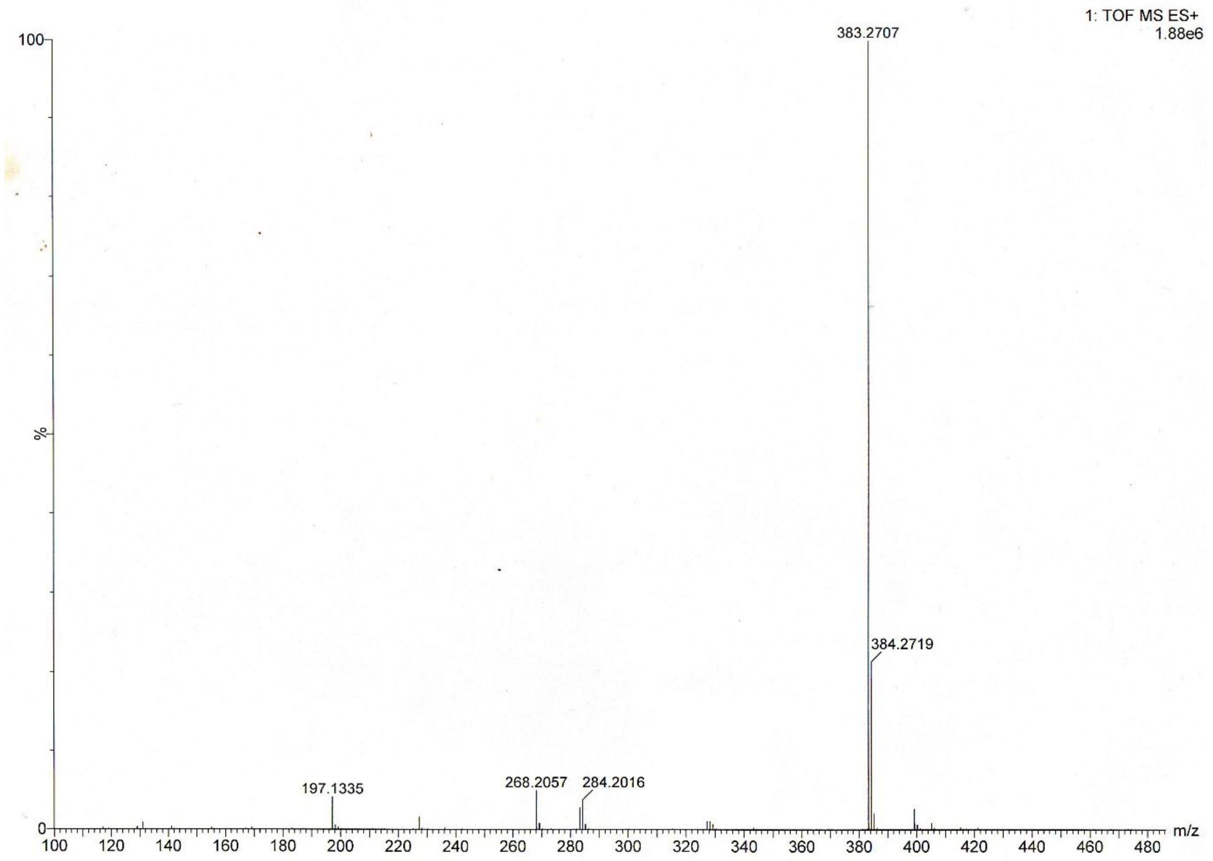

Figure S24: HRMS spectra of (**2d**)

(4-(1-cyclohexyl-3-phenylprop-2-yn-1-yl)piperazin-1-yl)(furan-2-yl)methanone (**2e**):

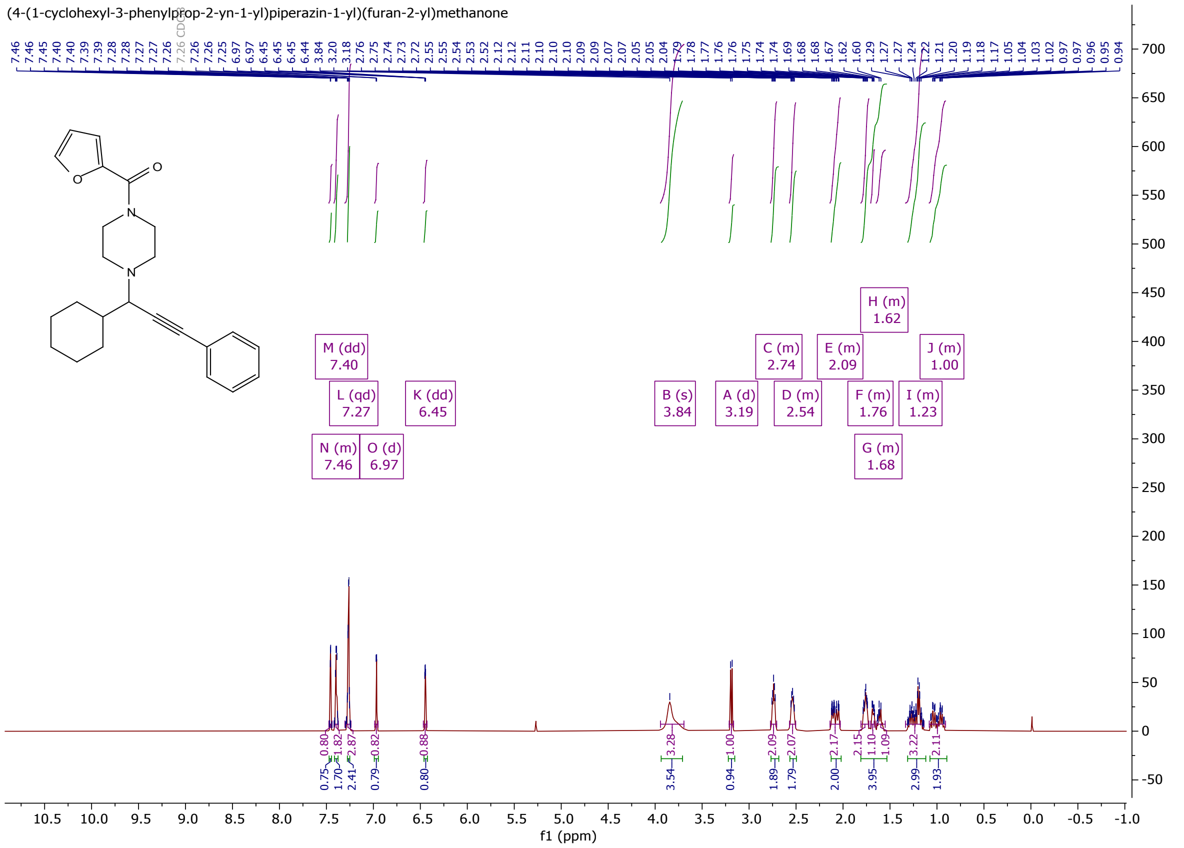

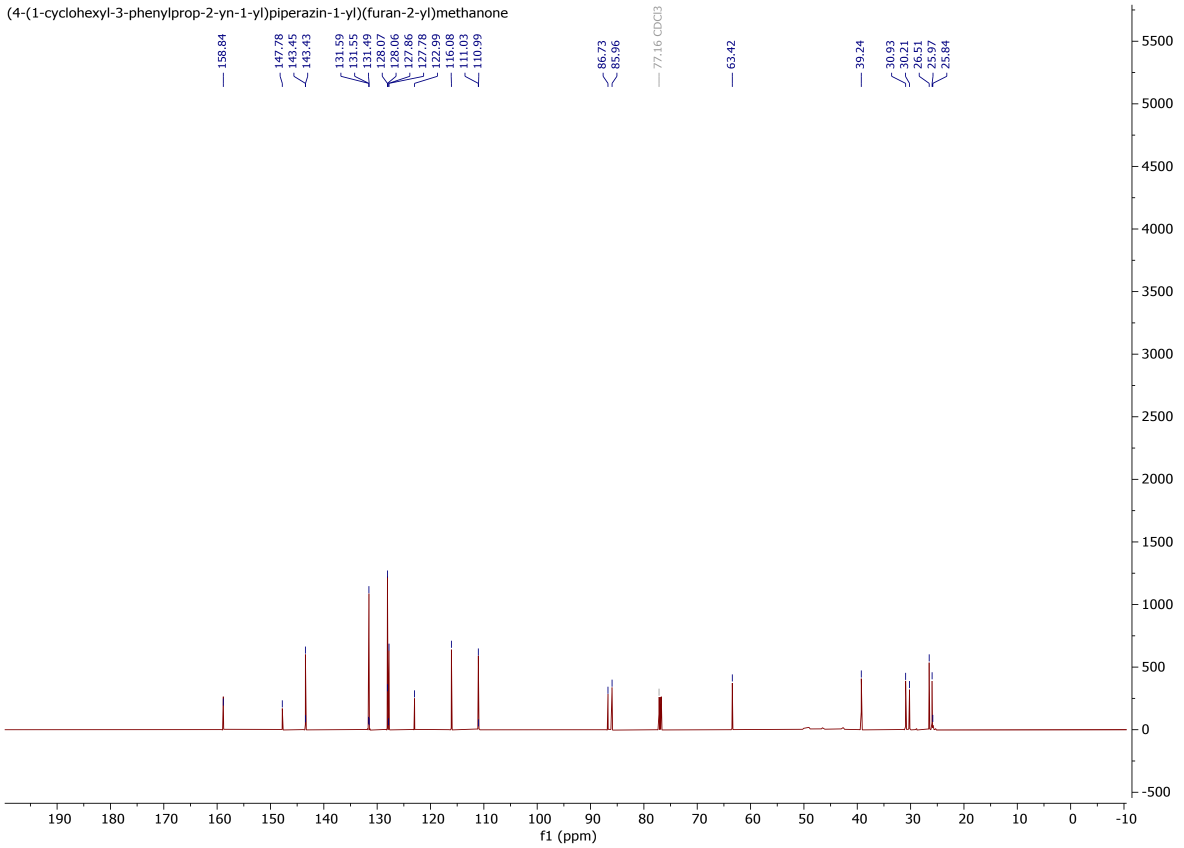

Figure S25: NMR spectra of (**2e**)

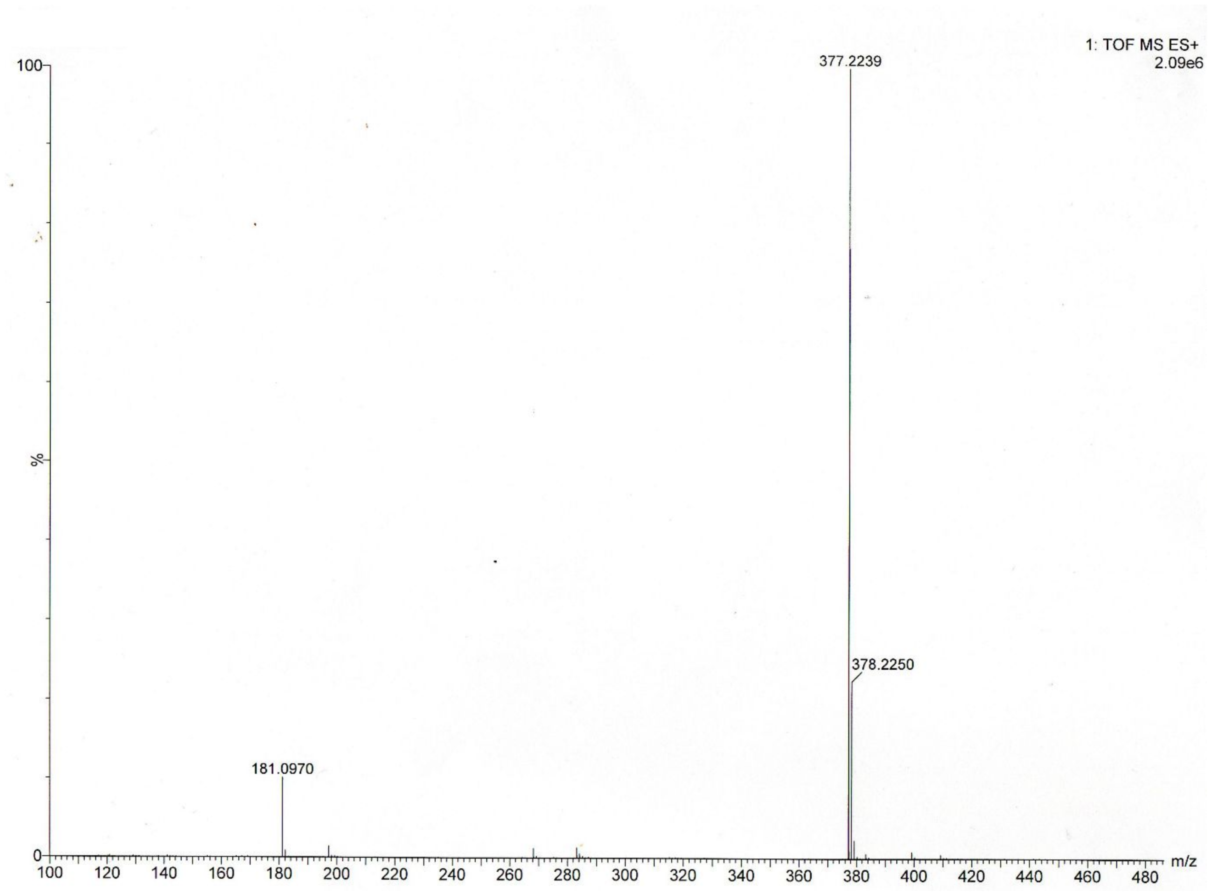

Figure S26: HRMS spectra of (**2e**)

1-(1-phenylhept-1-yn-3-yl)pyrrolidine (**2f**):

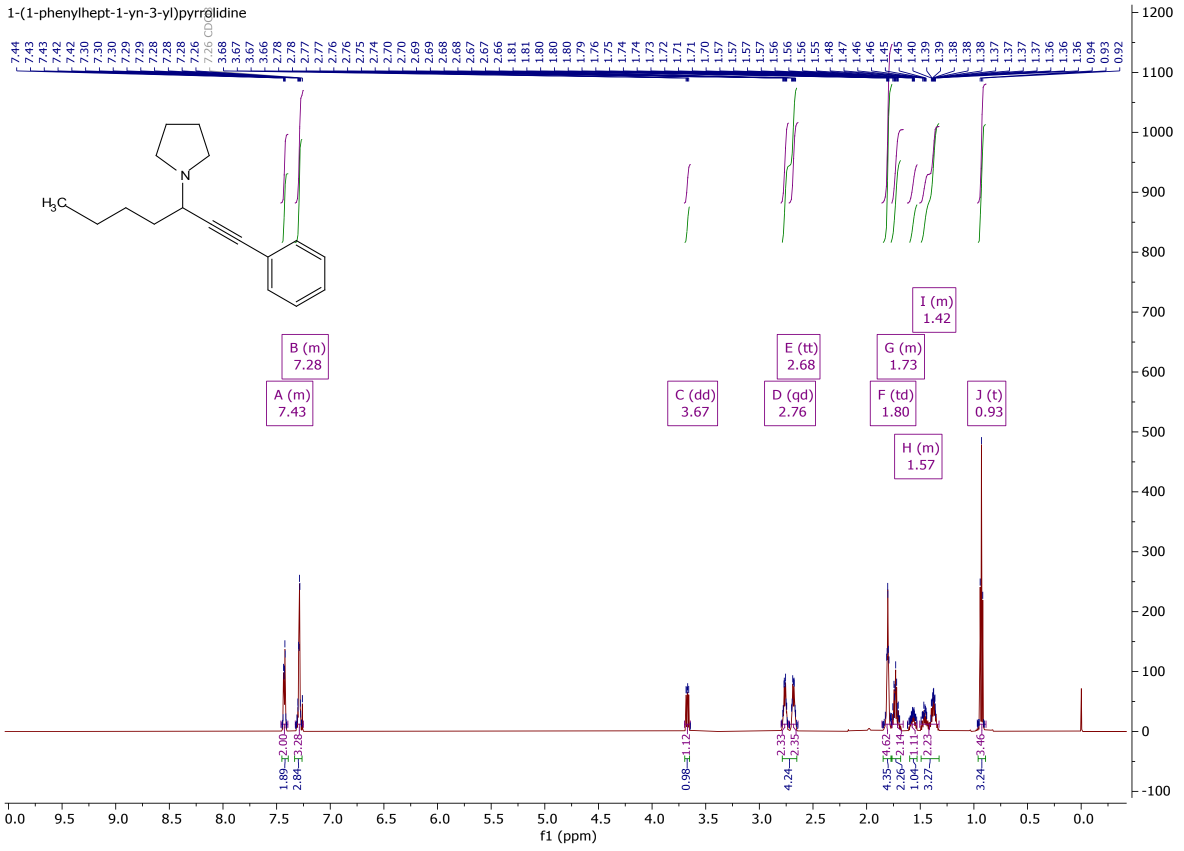

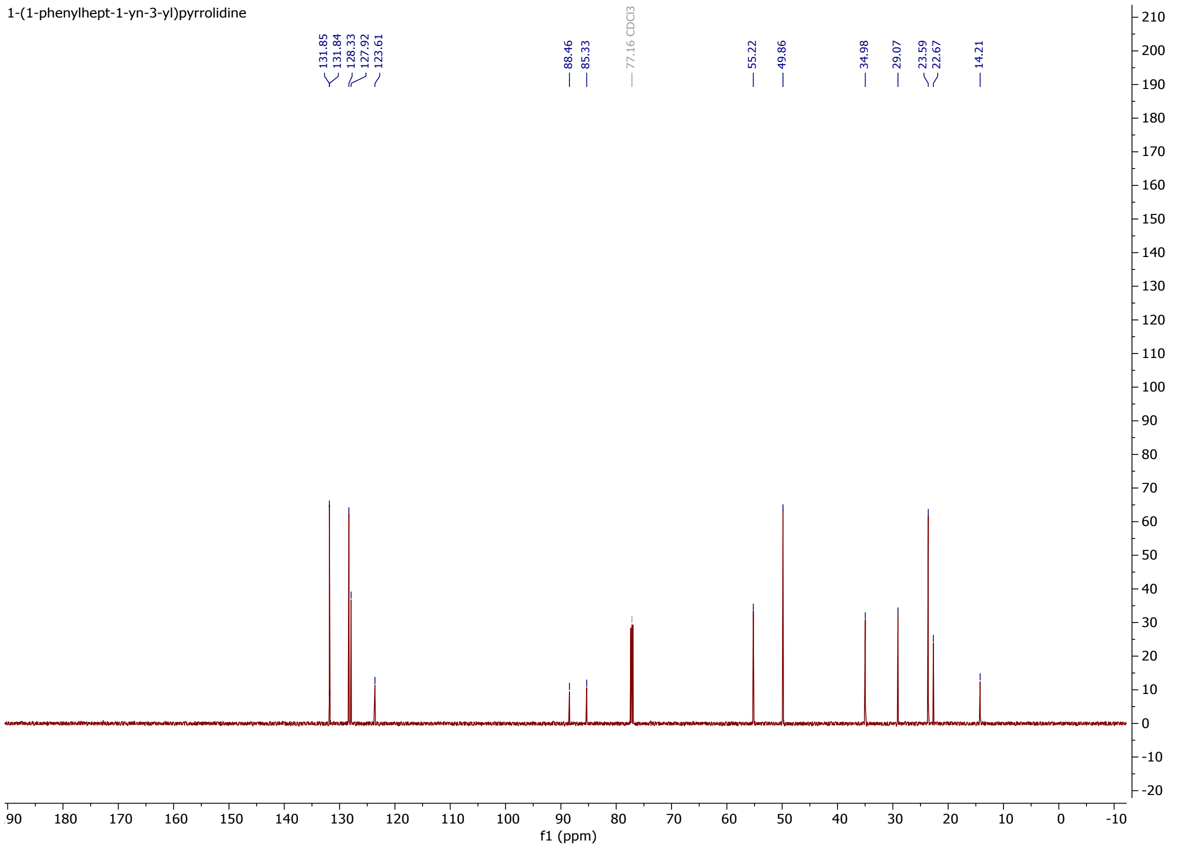

Figure S27: NMR spectra of (**2f**)

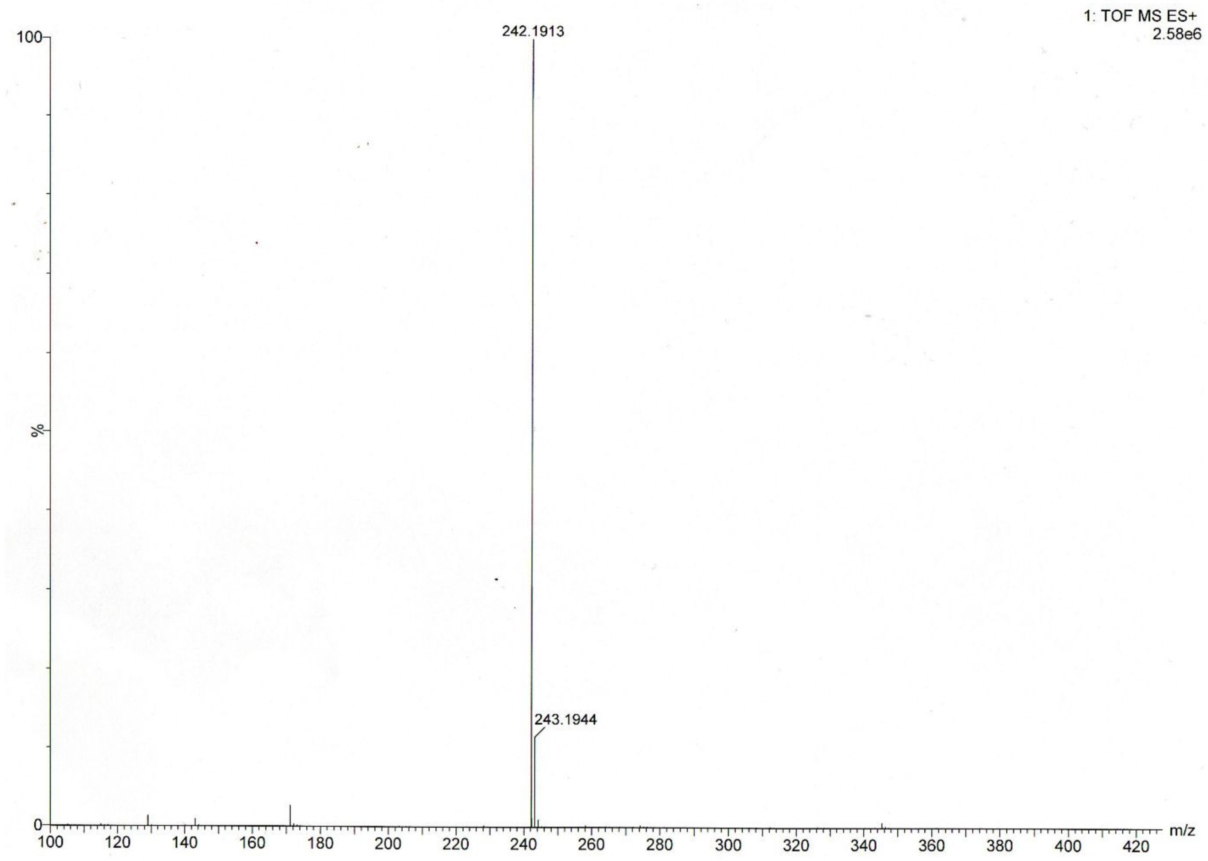

Figure S28: HRMS spectra of (**2f**)

1-(1-phenylhept-1-yn-3-yl)piperidine (**2g**):

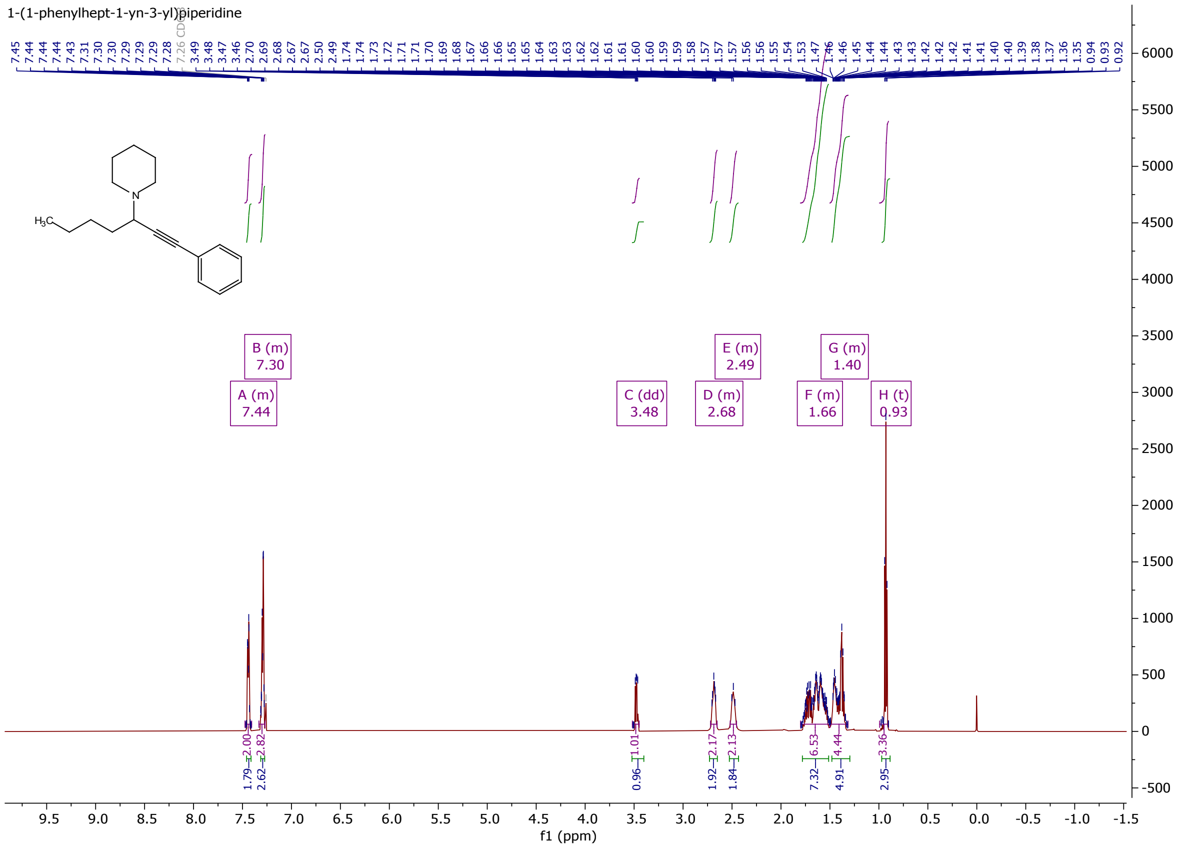

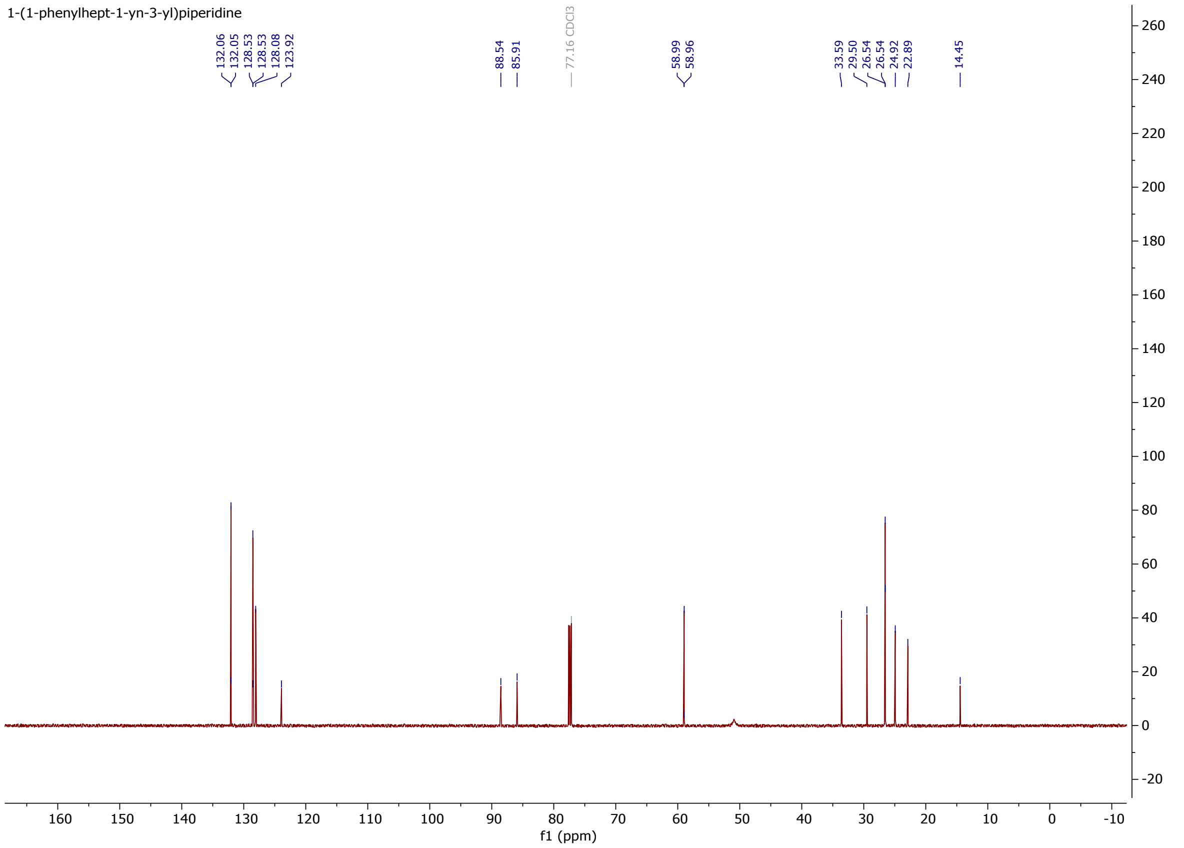

Figure S29: NMR spectra of (**2g**)

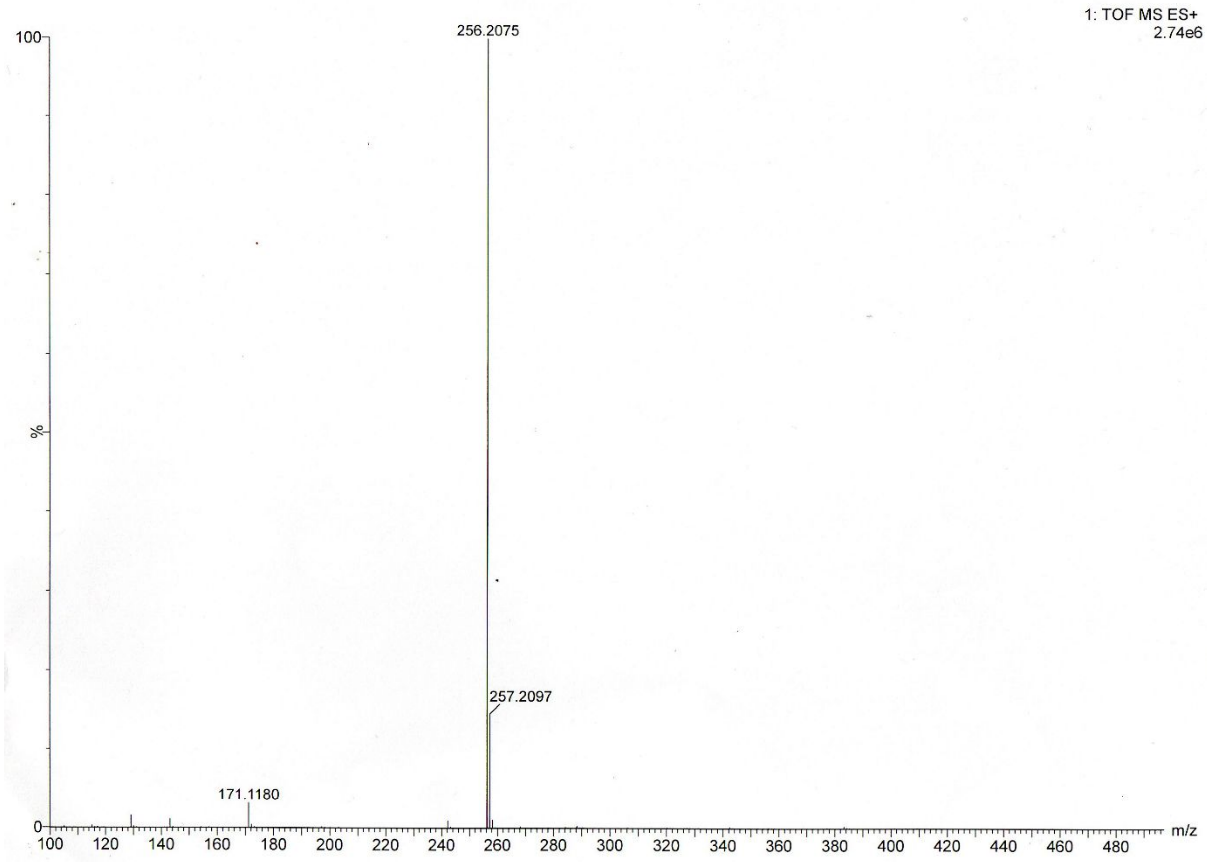

Figure S30: HRMS spectra of (**2g**)

4-(1-phenylhept-1-yn-3-yl)morpholine (**2h**):

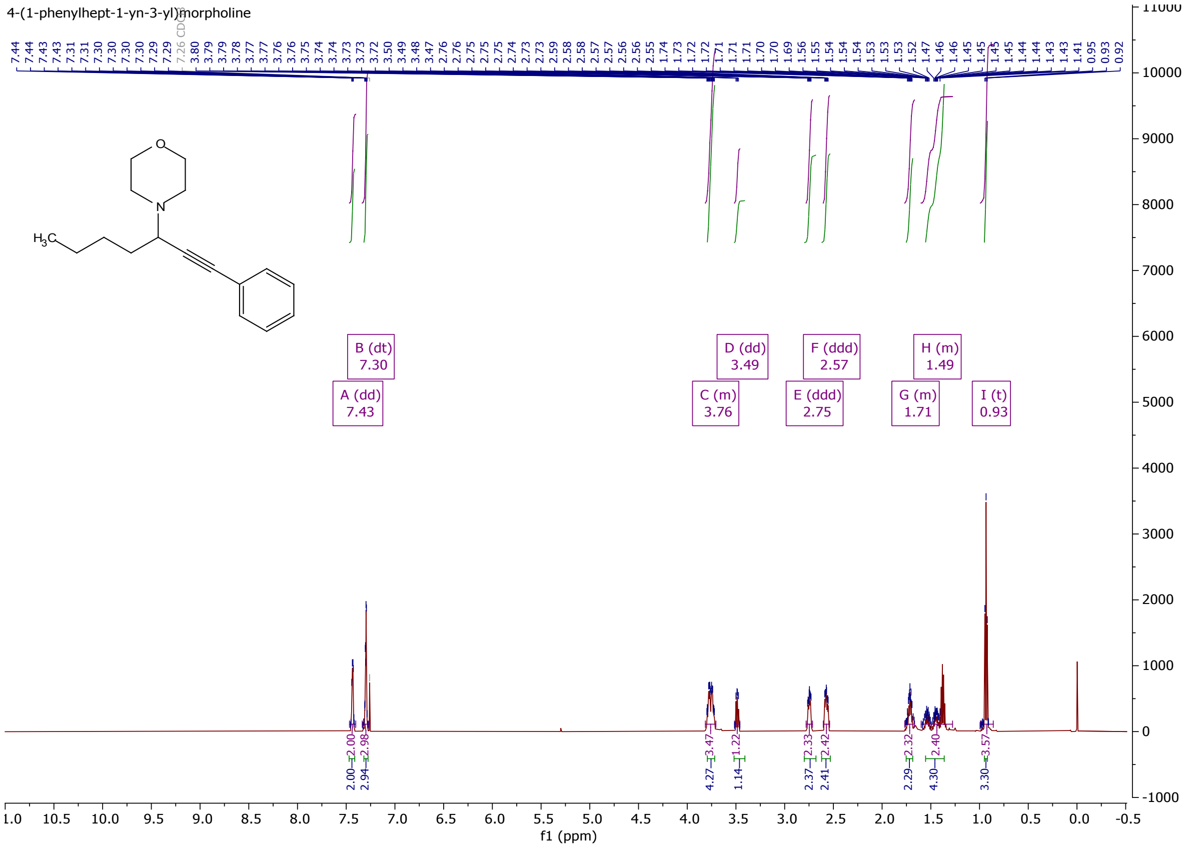

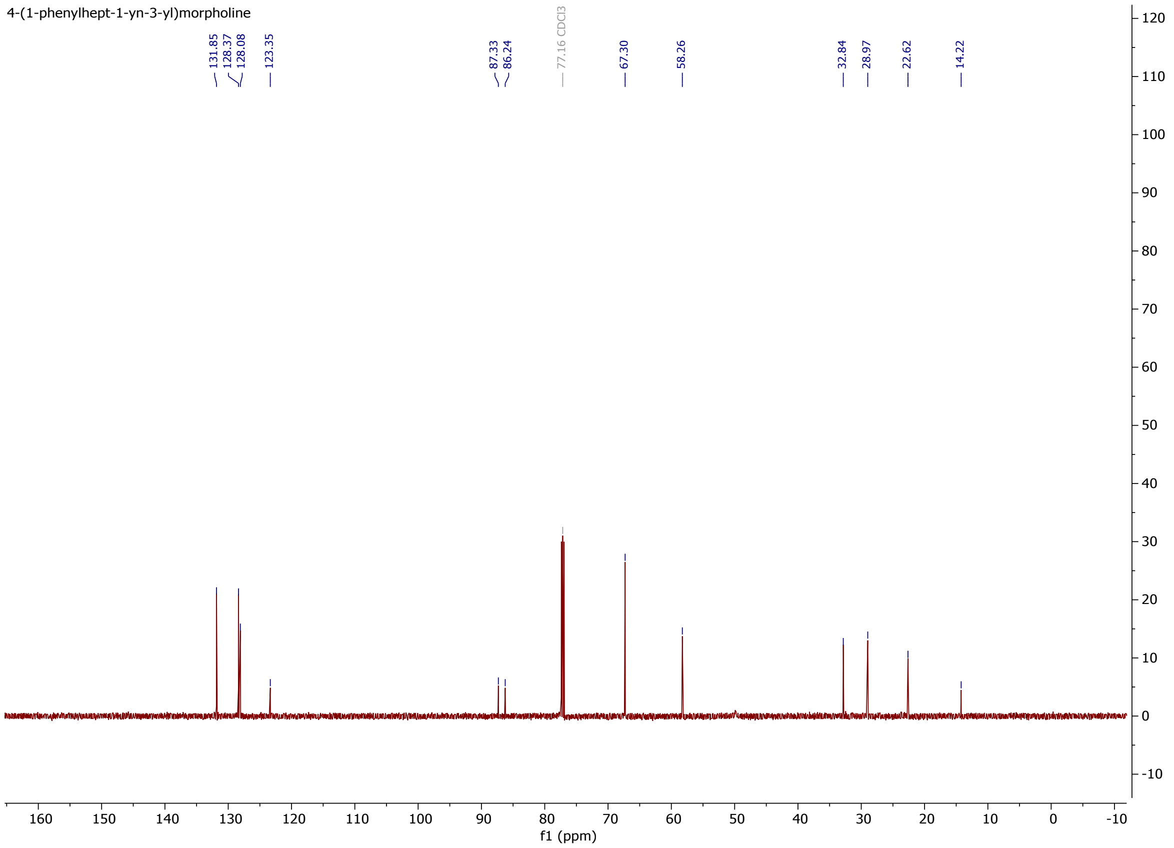

Figure S31: NMR spectra of (**2h**)

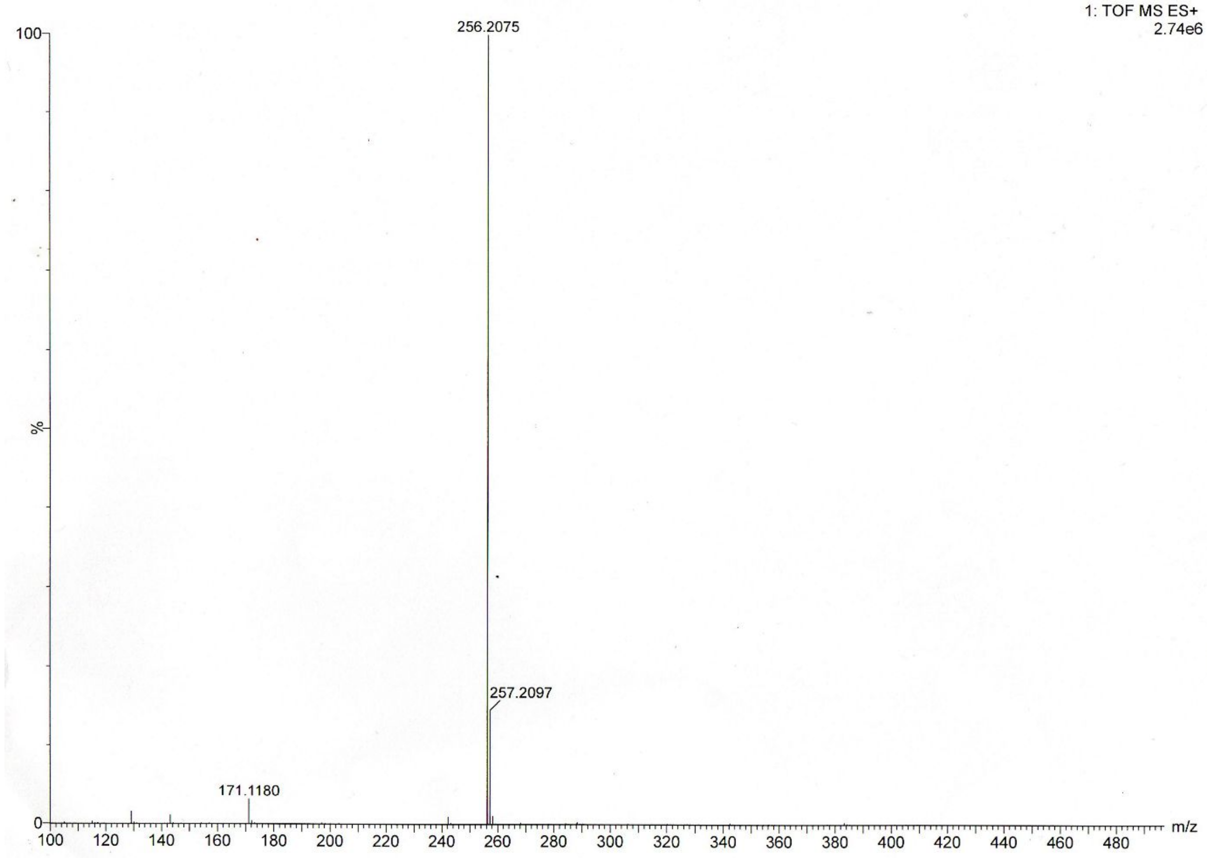

Figure S32: HRMS spectra of (**2h**)

tert-butyl 4-(1-phenylhept-1-yn-3-yl)piperazine-1-carboxylate (**2i**):

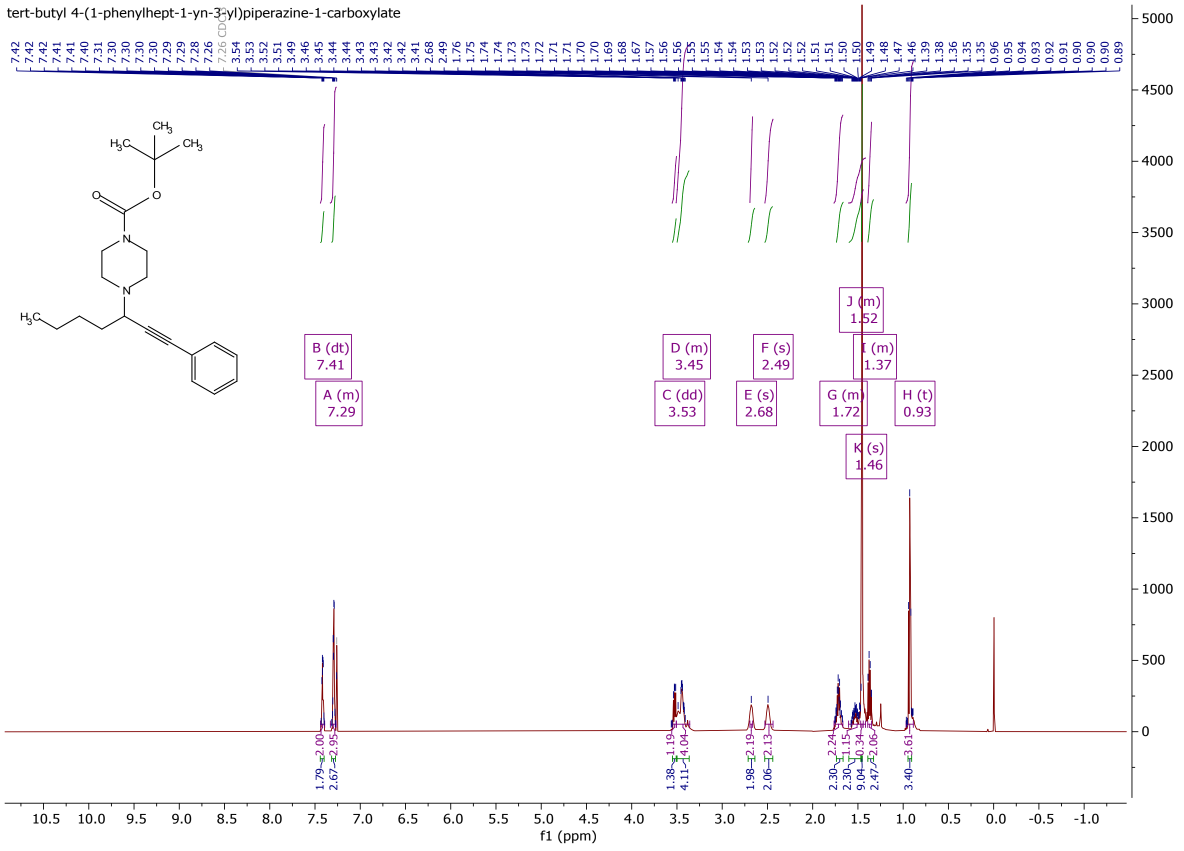

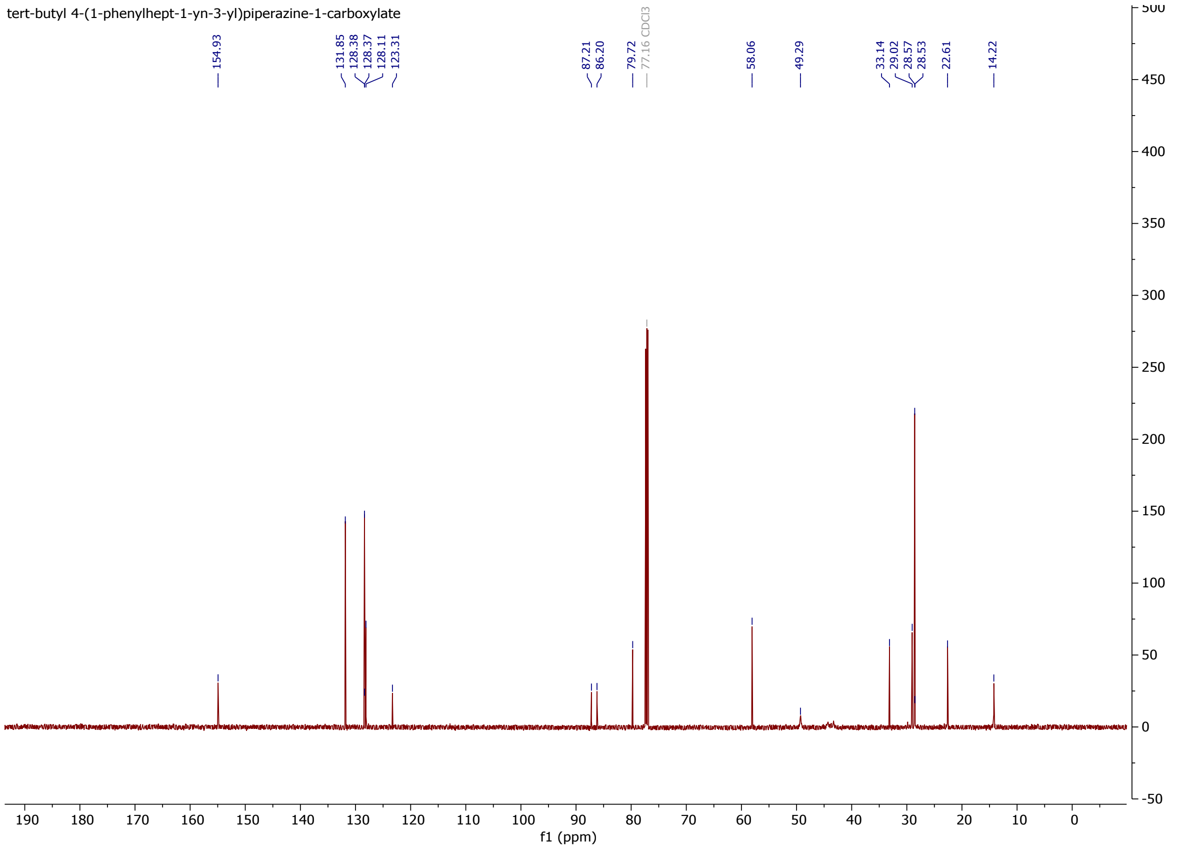

Figure S33: NMR spectra of (**2i**)

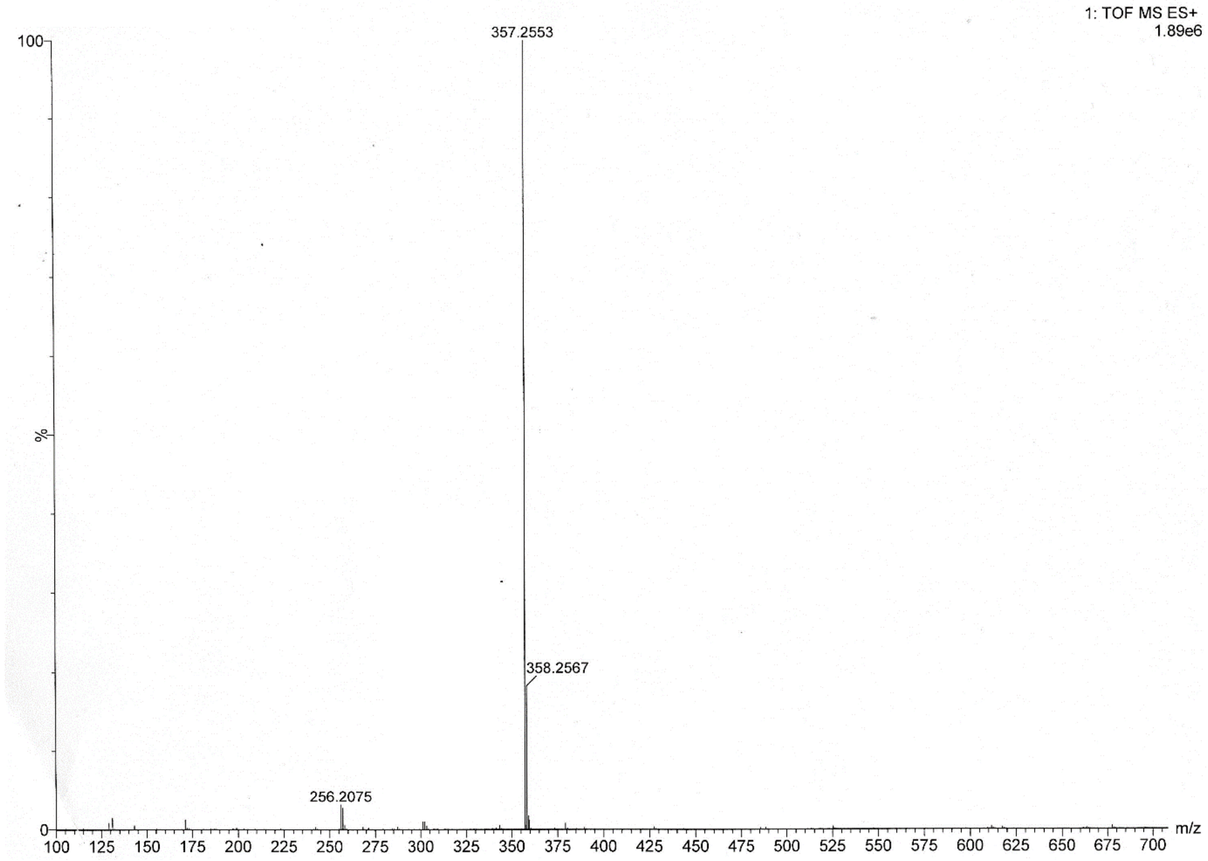

Figure S34: HRMS spectra of (**2i**)

furan-2-yl(4-(1-phenylhept-1-yn-3-yl)piperazin-1-yl)methanone (**2j**):

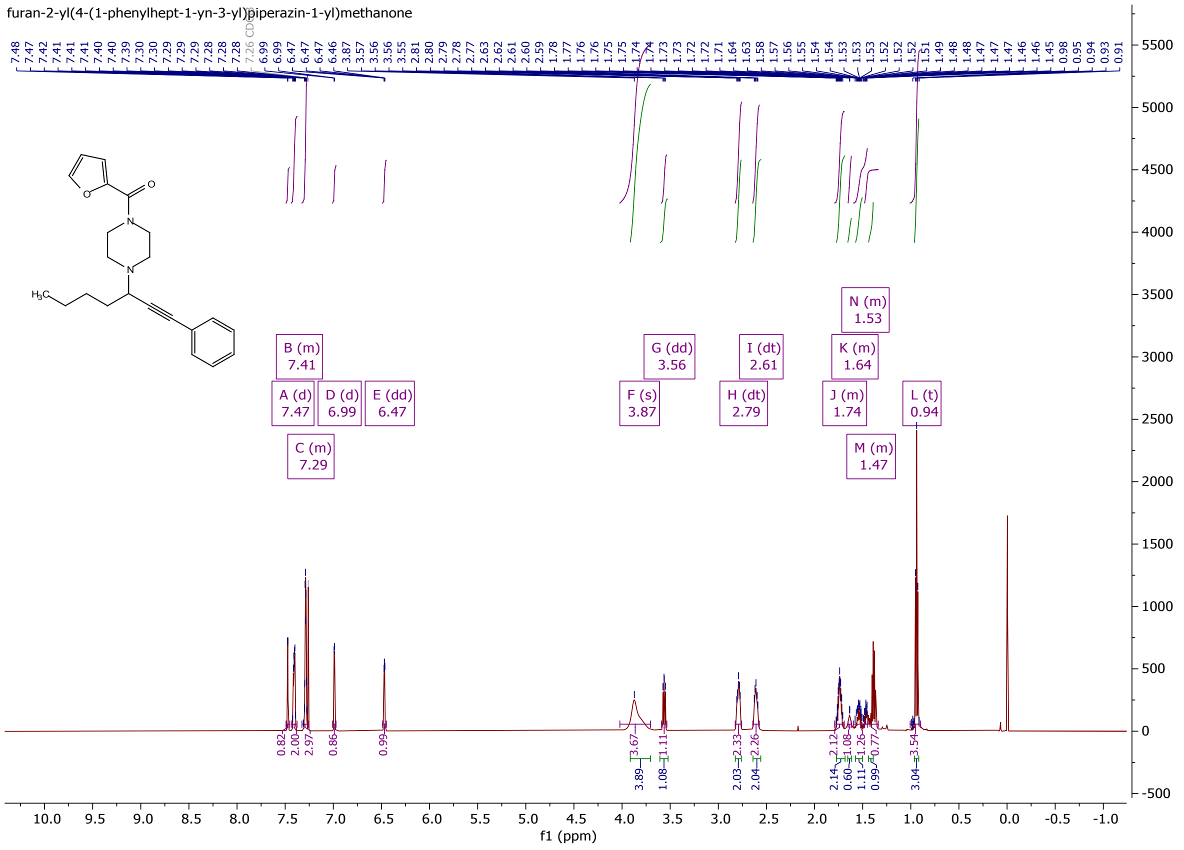

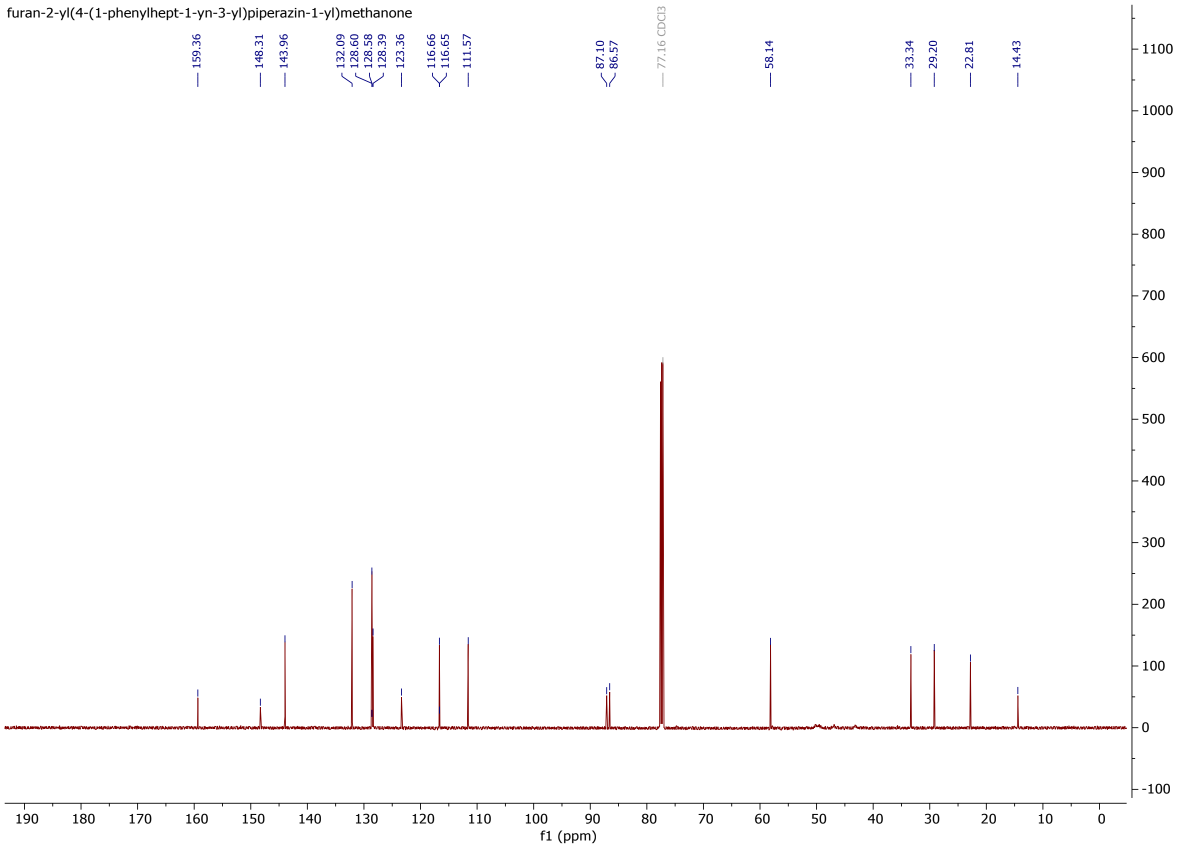

Figure S35: NMR spectra of (**2j**)

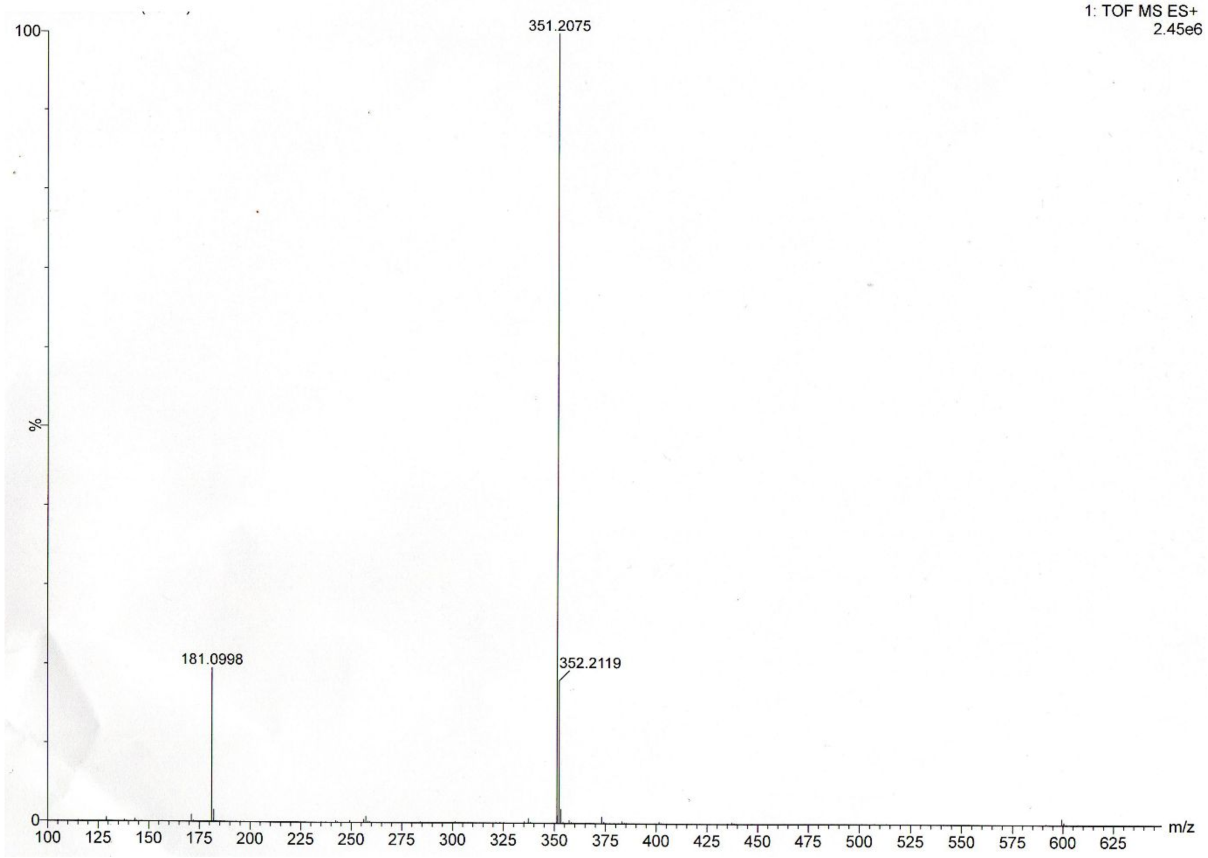

Figure S36: HRMS spectra of (**2j**)

7-cyclohexyl-7-(pyrrolidin-1-yl)hept-5-yn-1-ol (**2k**):

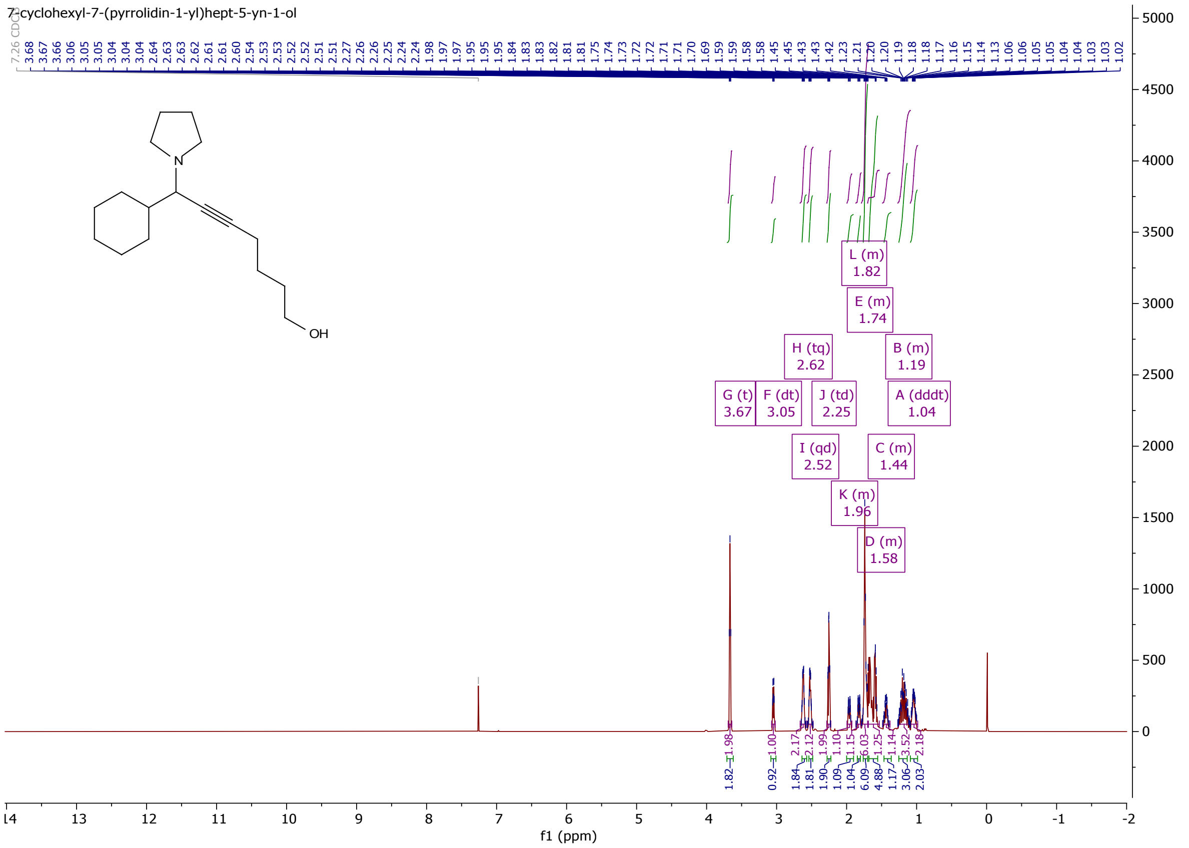

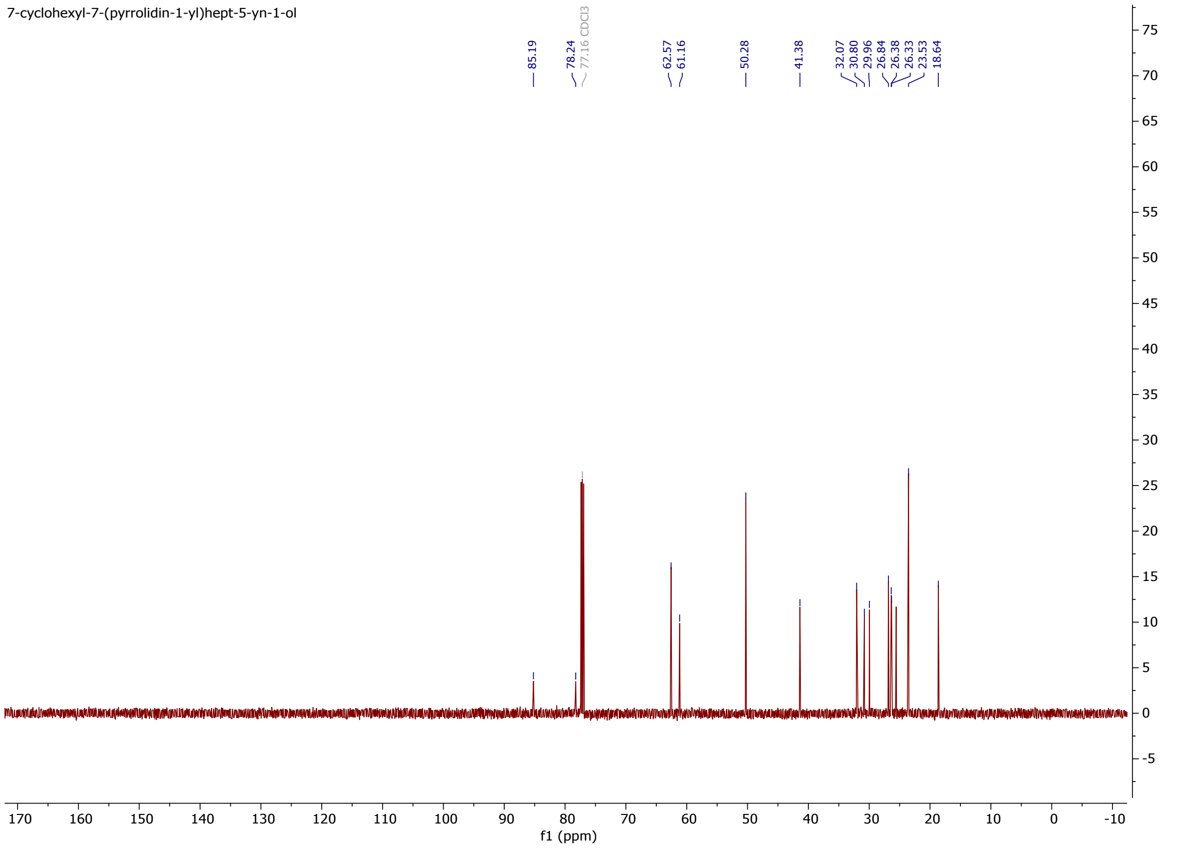

Figure S37: NMR spectra of (**2k**)

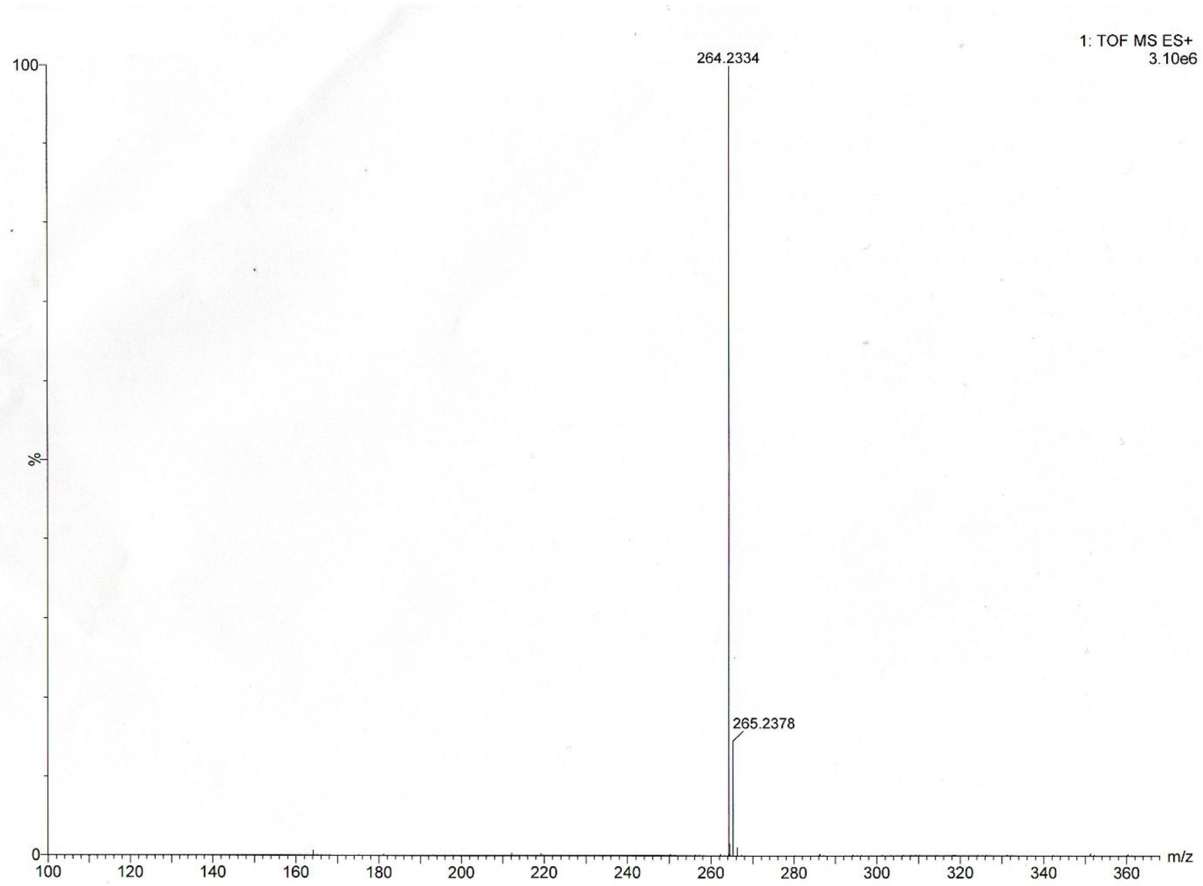

Figure S38: HRMS spectra of (**2k**)

6-cyclohexyl-6-(pyrrolidin-1-yl)hex-4-yn-1-ol (**2l**):

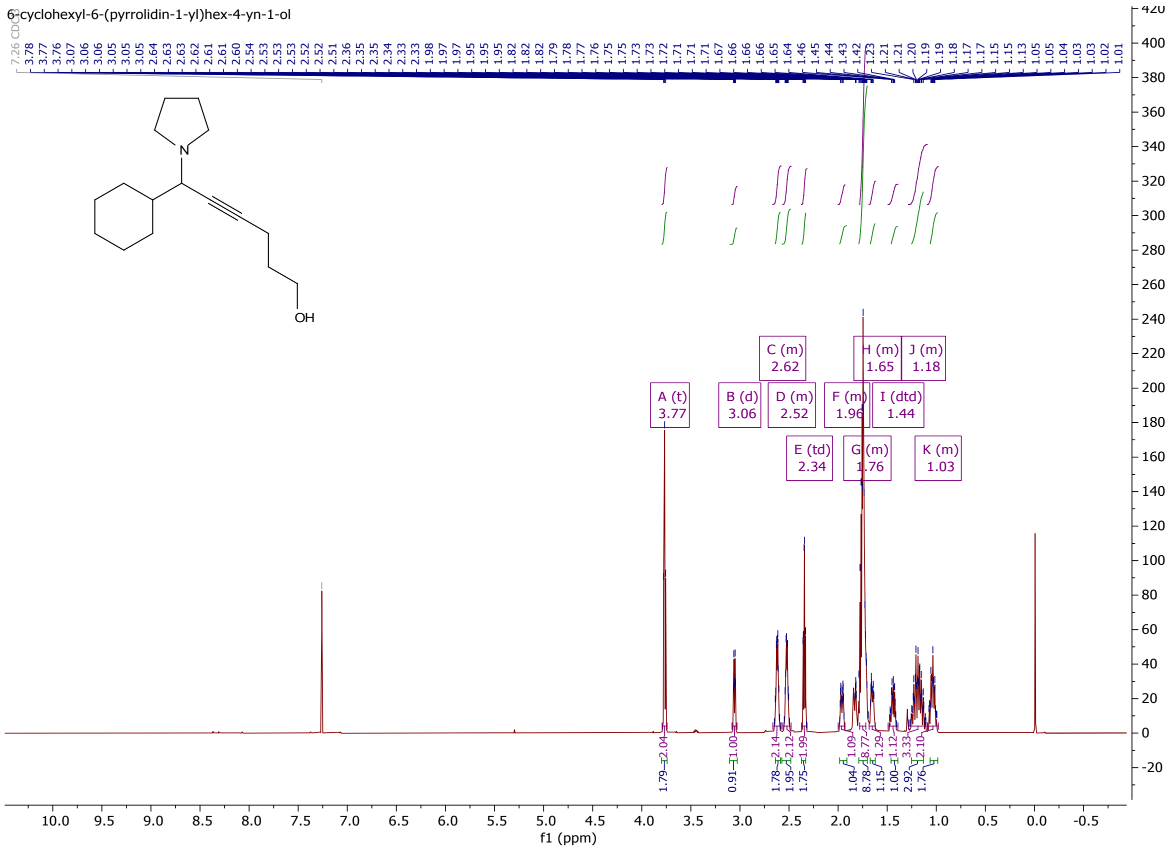

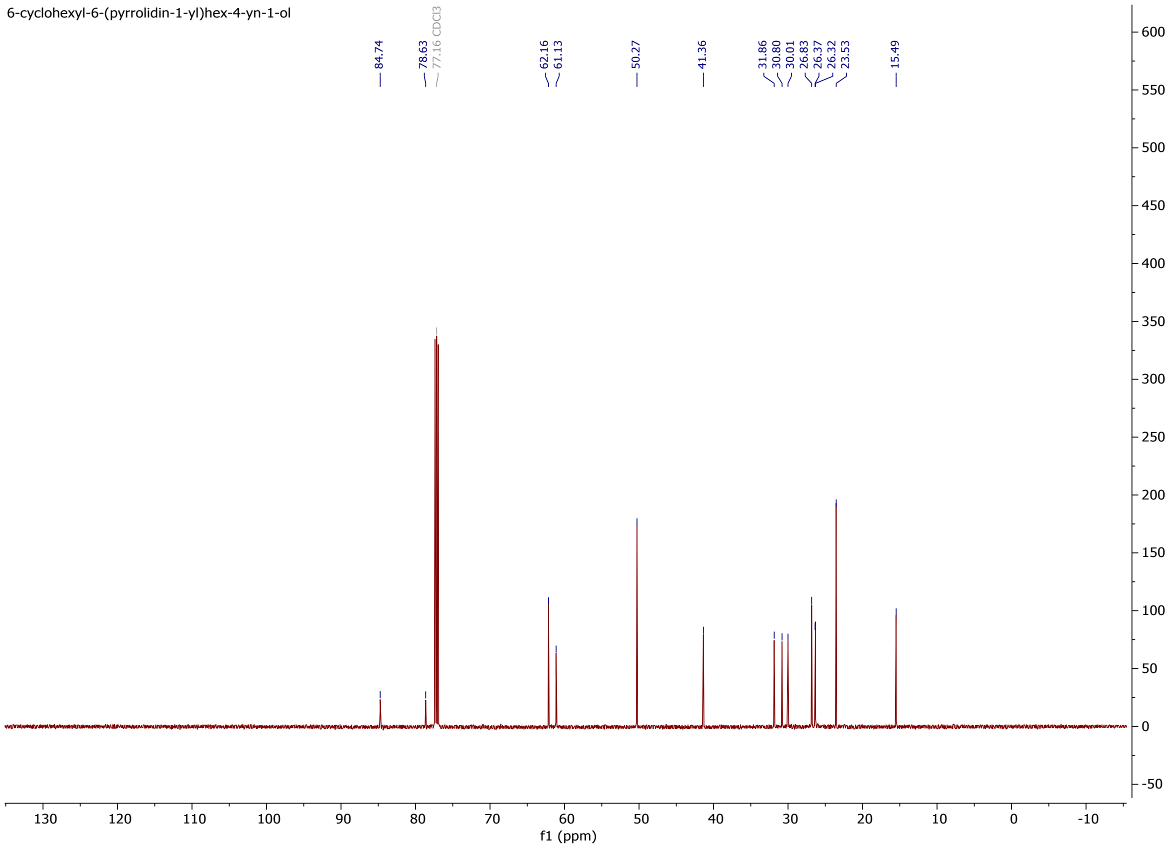

Figure S39: NMR spectra of (**2l**)

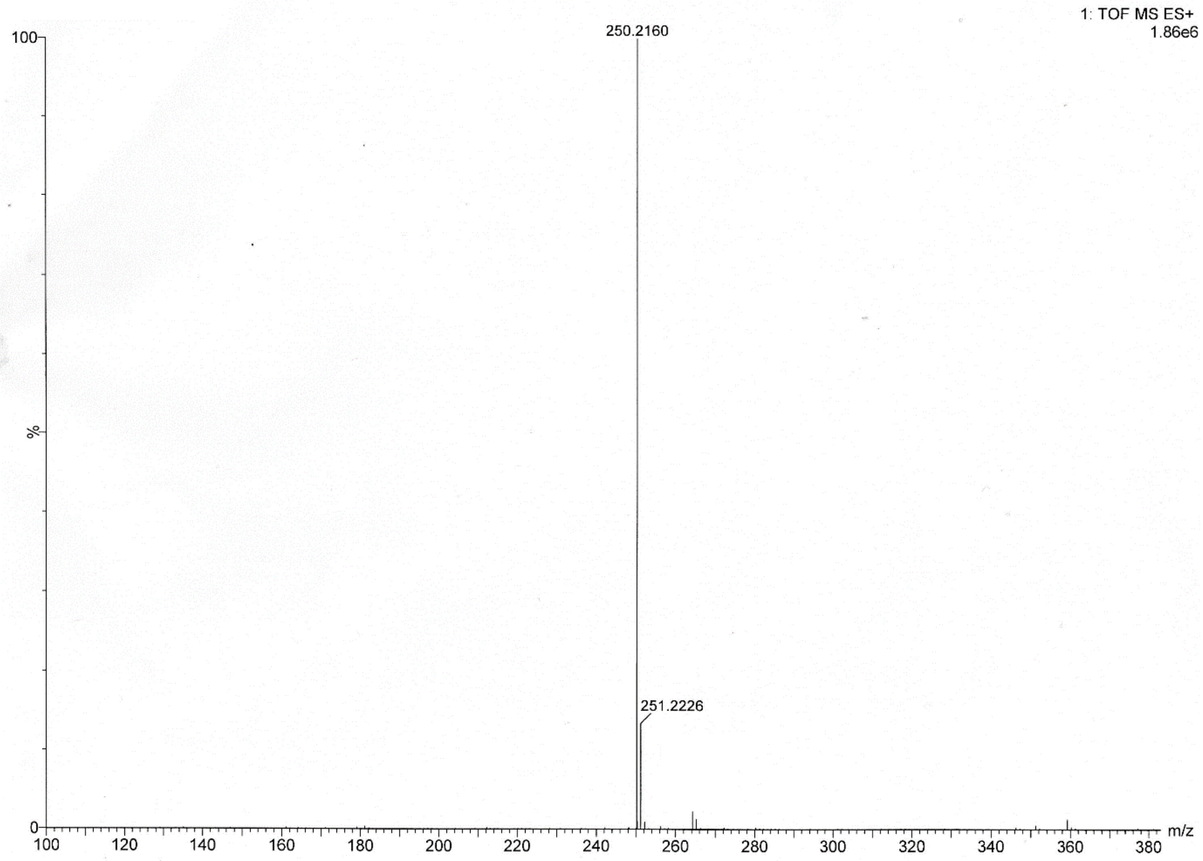

Figure S40: HRMS spectra of (**2l**)

5-cyclohexyl-5-(pyrrolidin-1-yl)pent-3-yn-1-ol (**2m**):

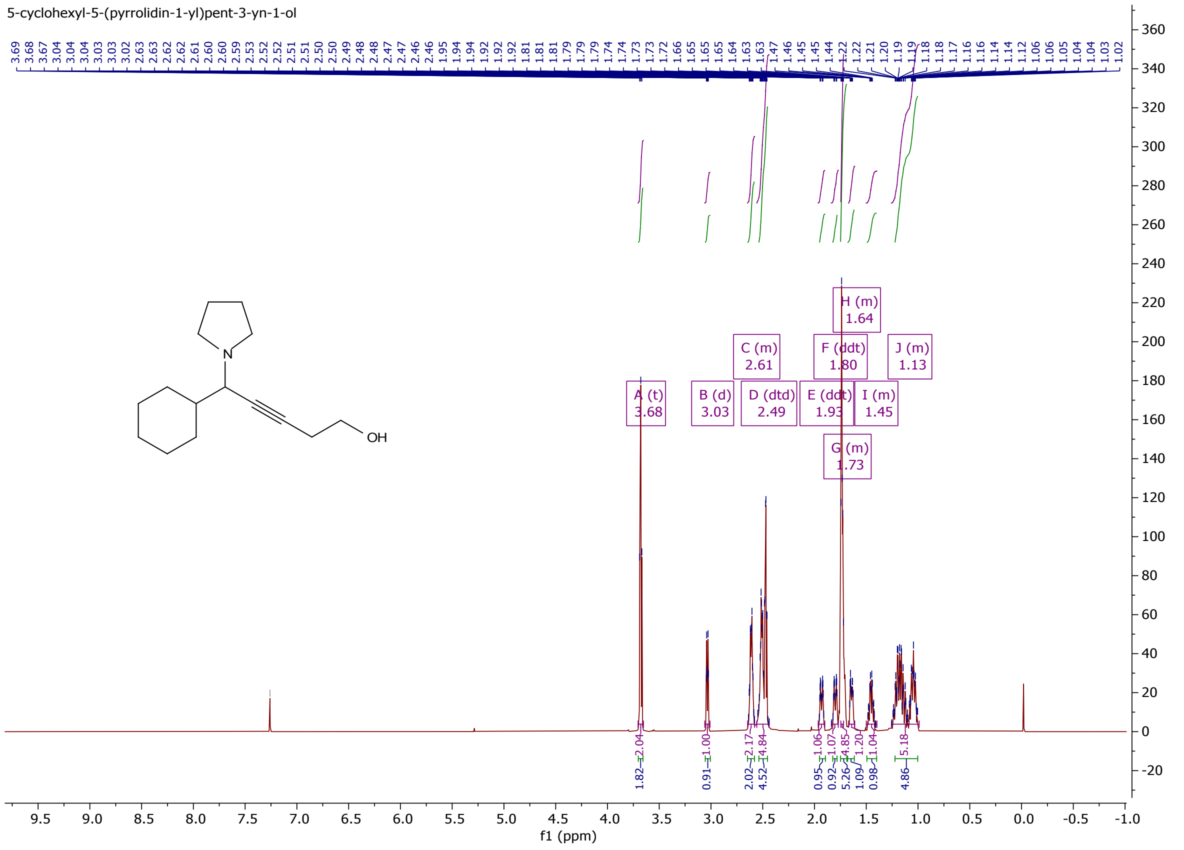

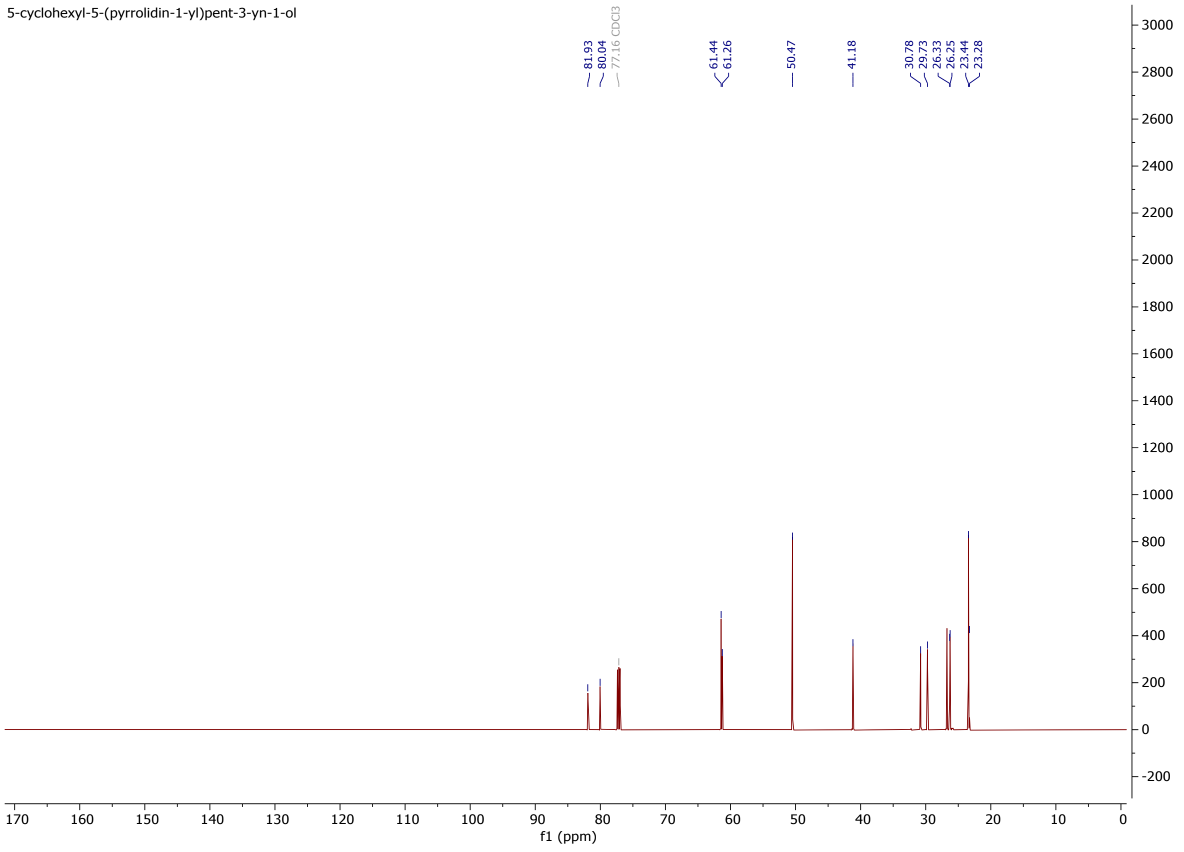

Figure S41: NMR spectra of (**2m**)

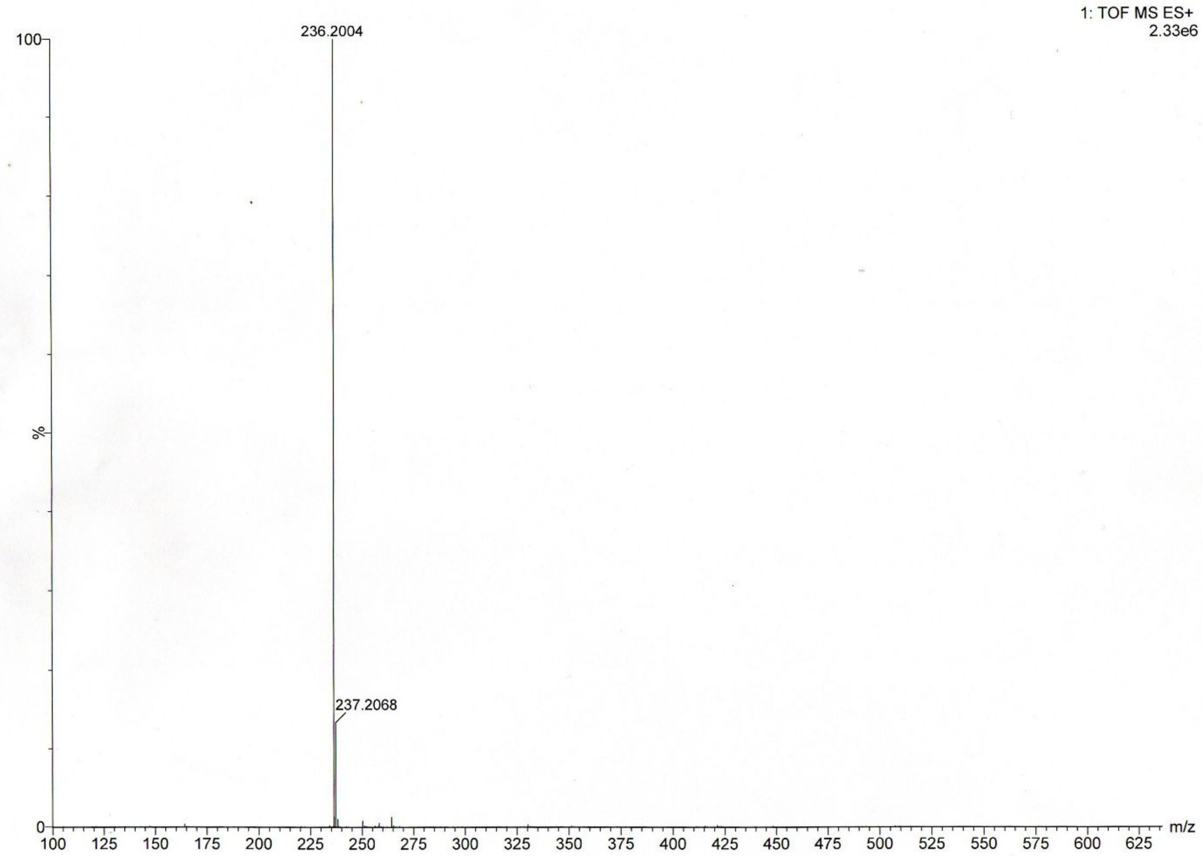

Figure S42: HRMS spectra of (**2m**)

1-(1-cyclohexyl-5-phenylpent-2-yn-1-yl)pyrrolidine (**2n**):

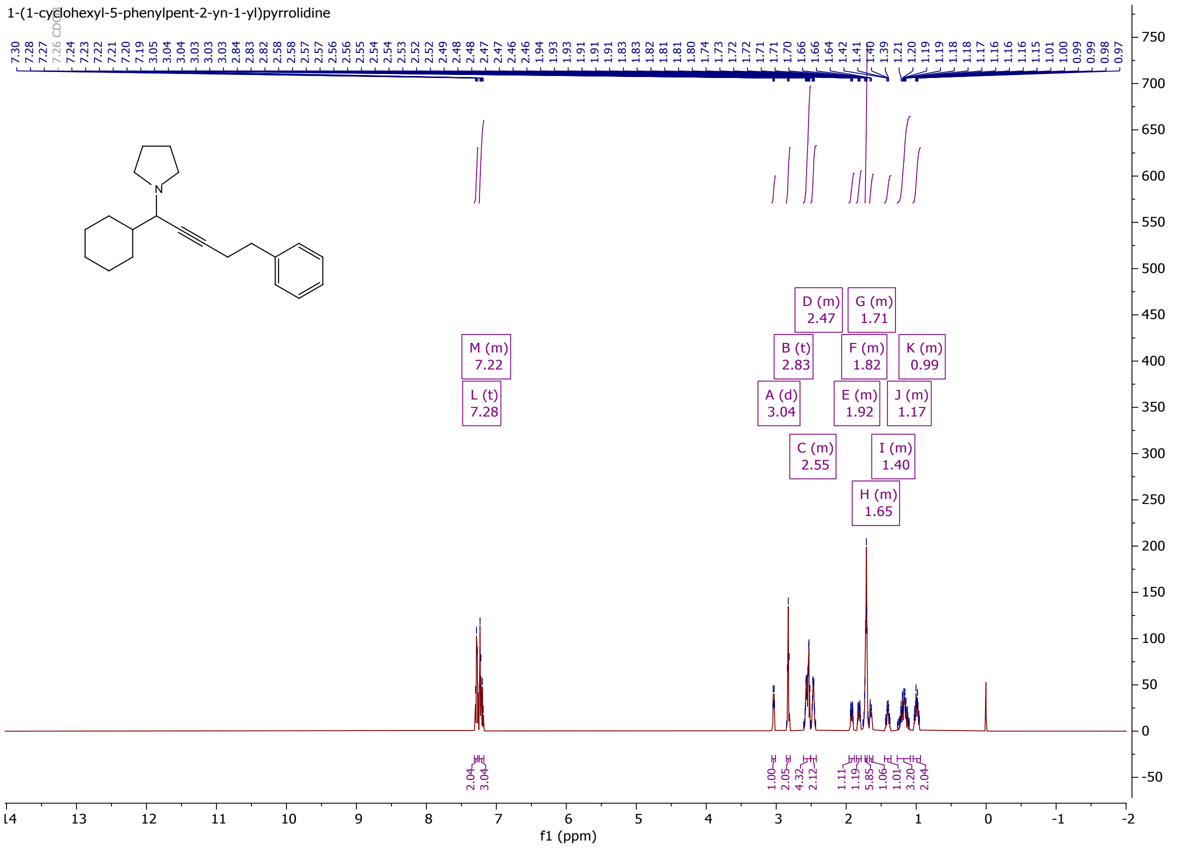

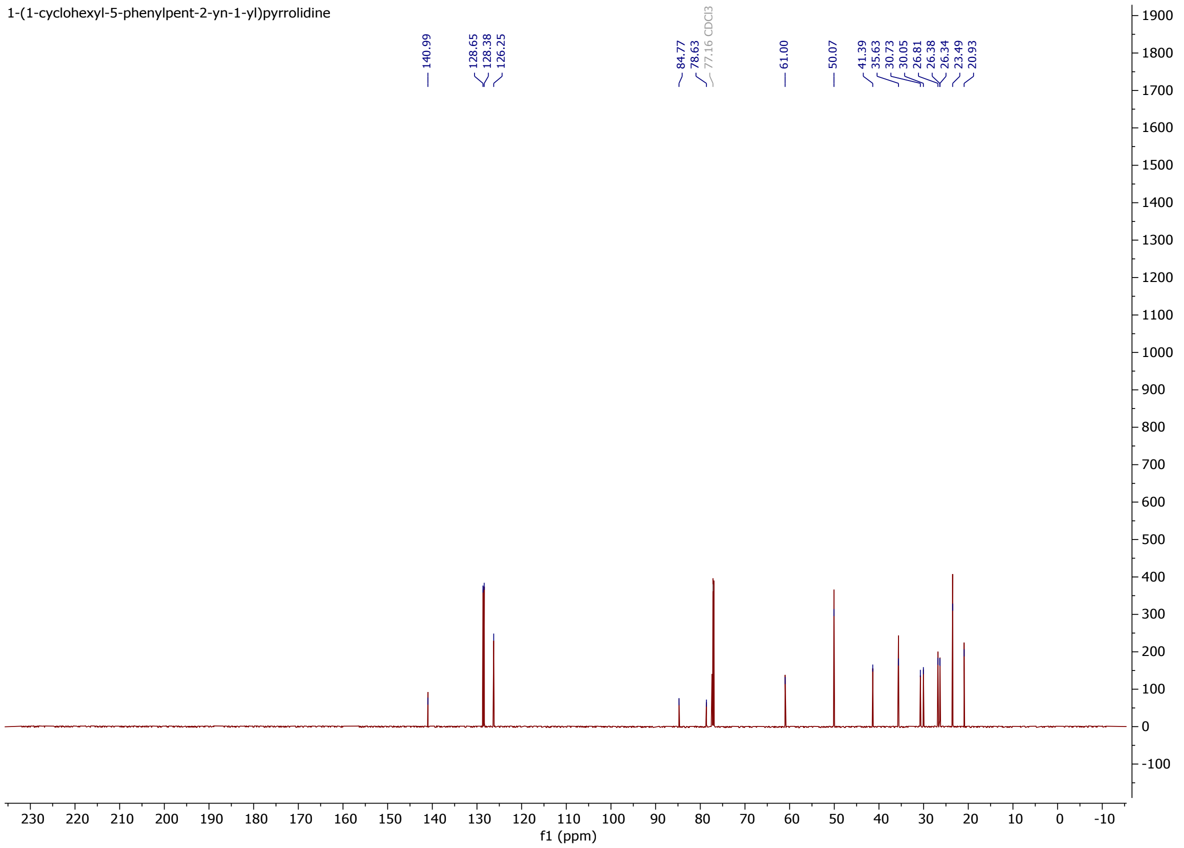

Figure S43: NMR spectra of (**2n**)

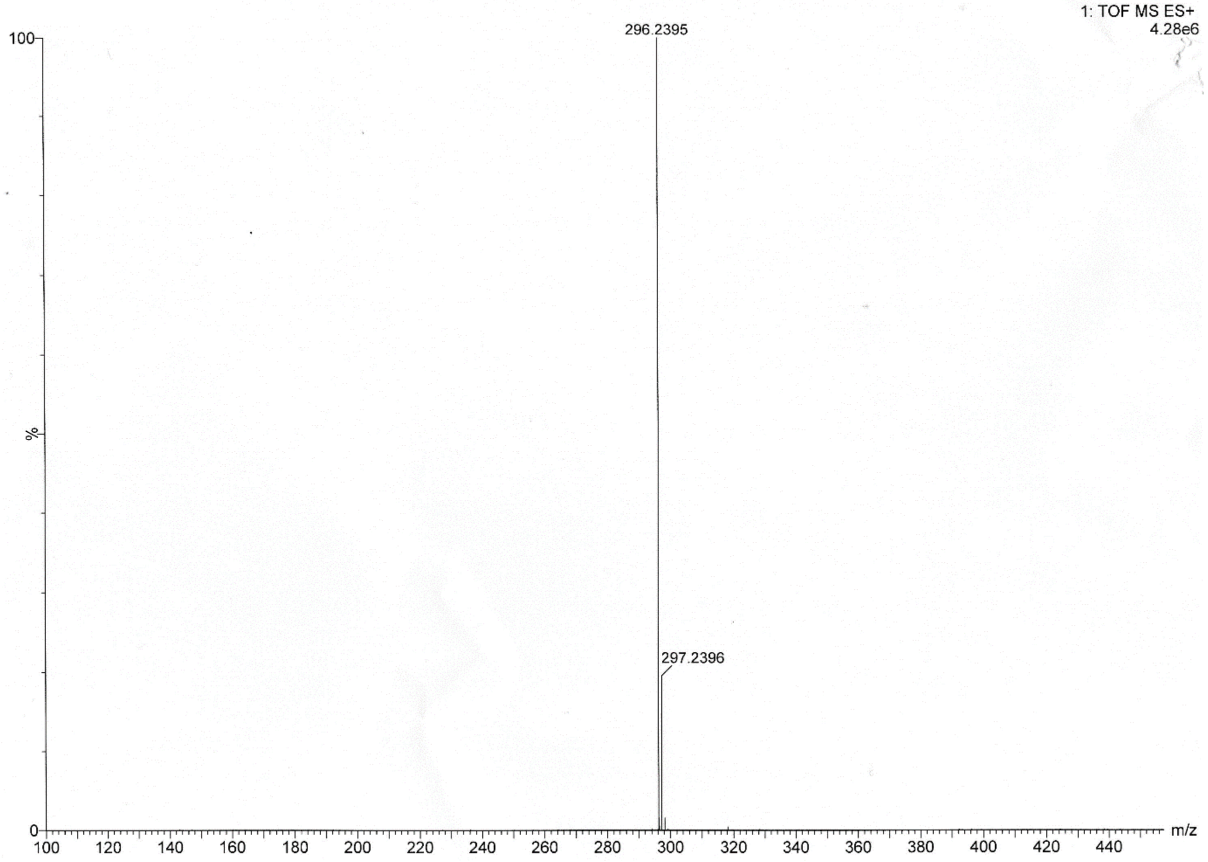

Figure S44: HRMS spectra of (**2n**)

1-(1-cyclohexyl-3-(4-methoxyphenyl)prop-2-yn-1-yl)pyrrolidine (**2o**):

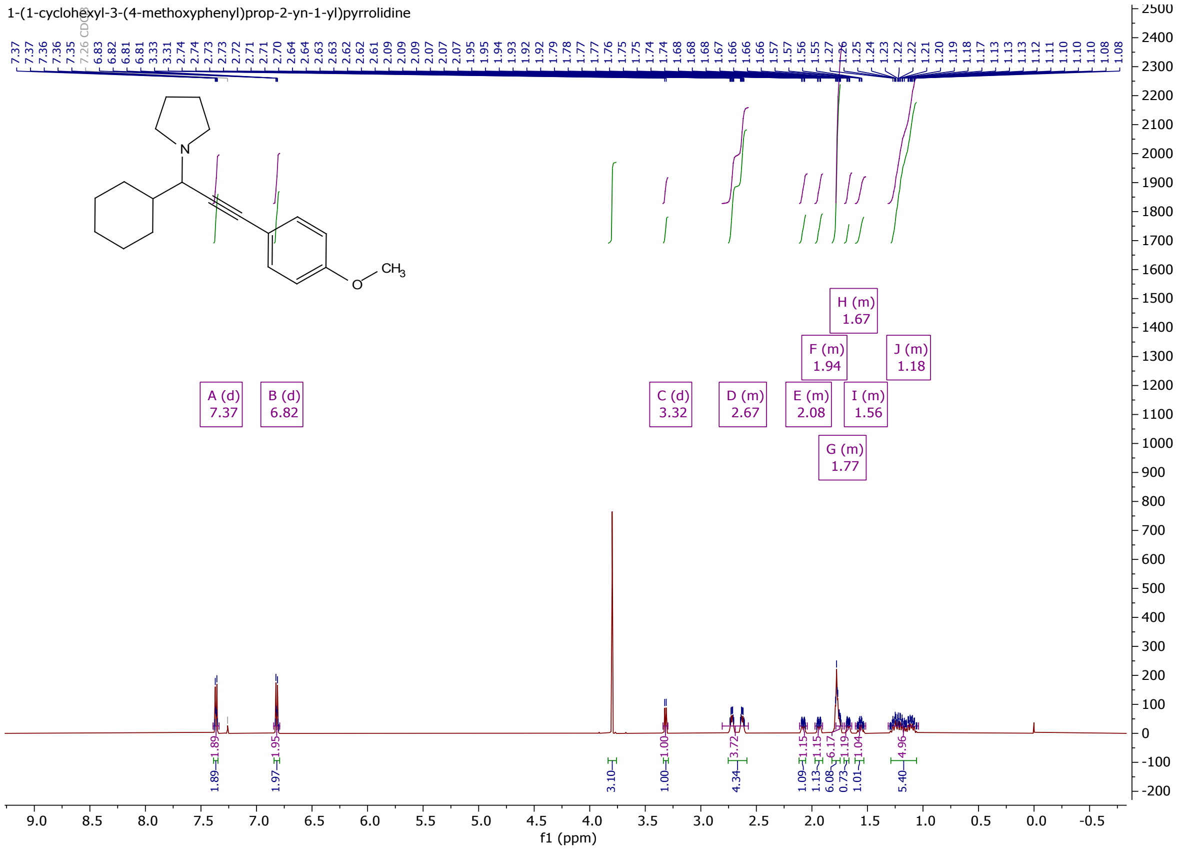

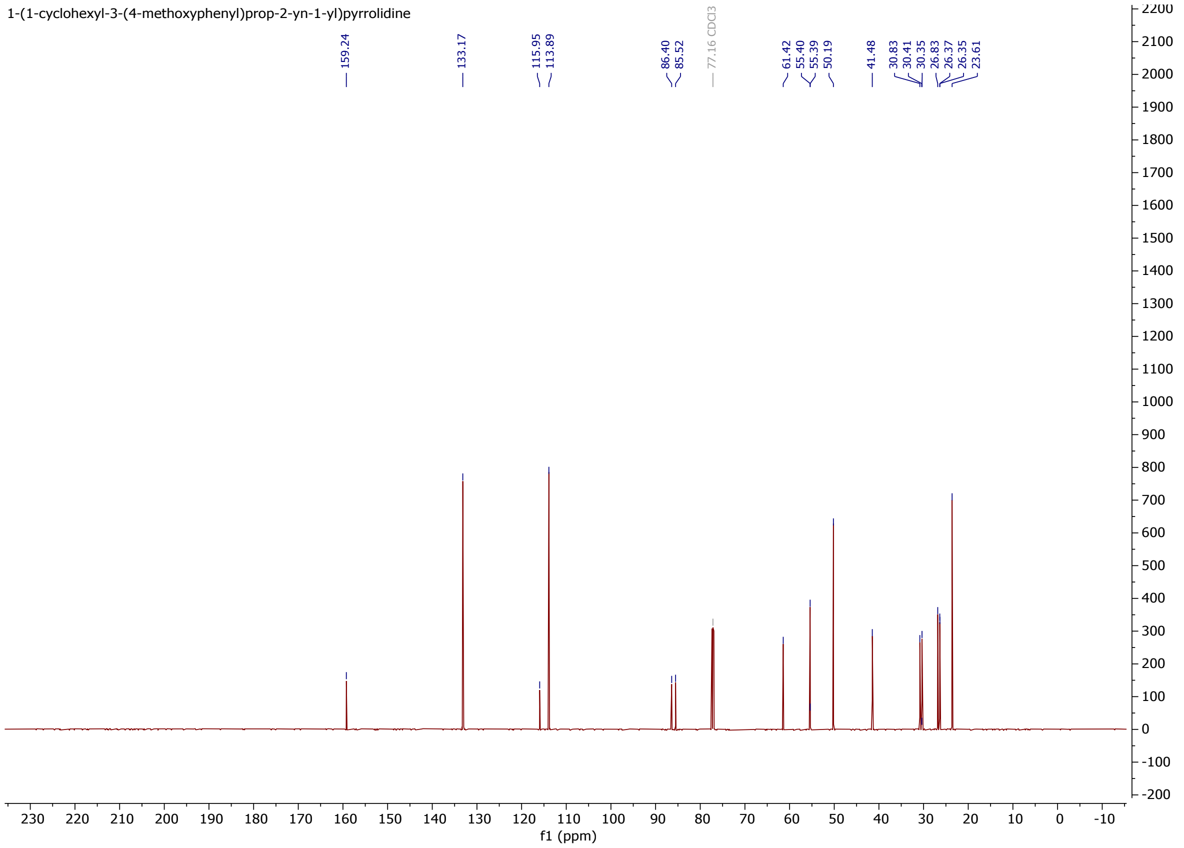

Figure S45: NMR spectra of (**2o**)

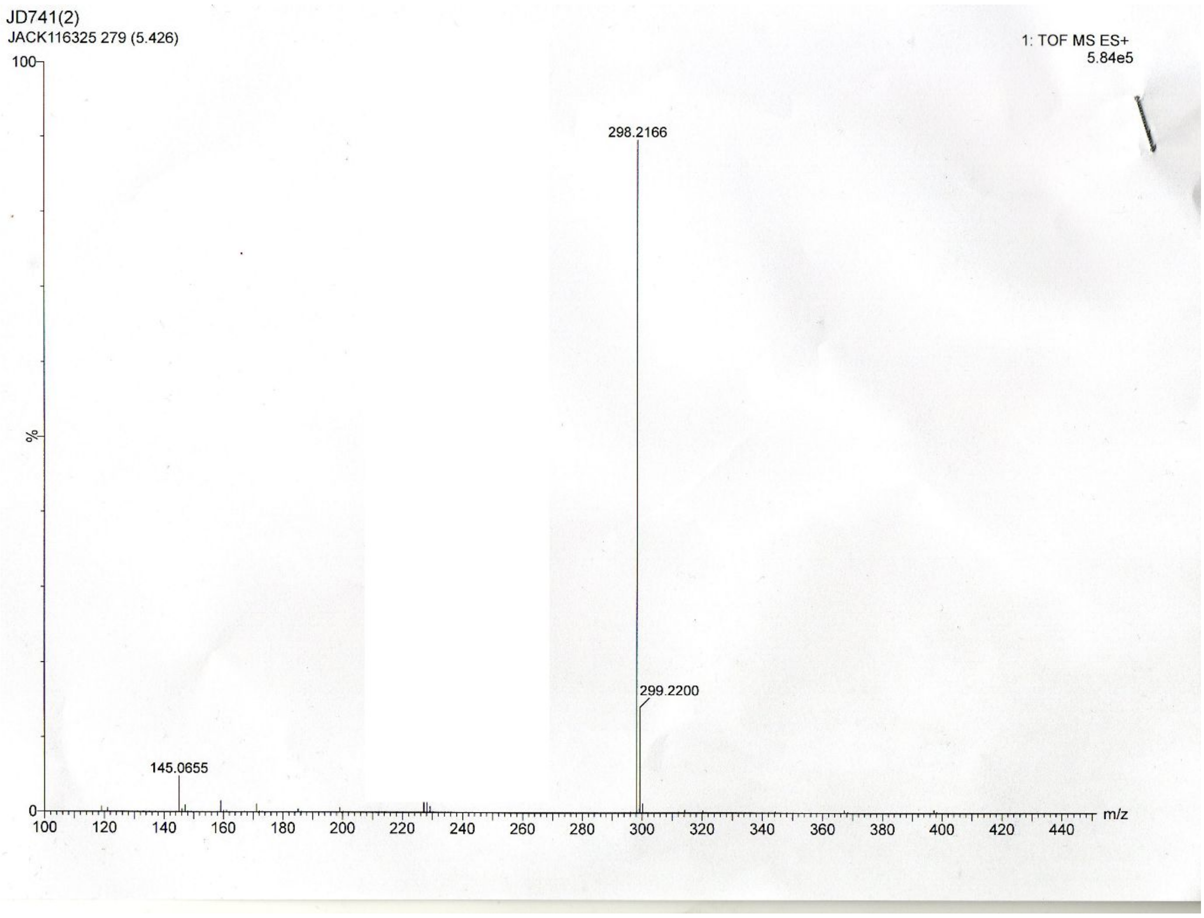

Figure S46: HRMS spectra of (**2o**)

(8S,9R,13R,14R,17R)-17-(3-cyclohexyl-3-(pyrrolidin-1-yl)prop-1-yn-1-yl)-13-methyl-7,8,9,11,12,13,14,15,16,17-decahydro-6H-cyclopenta[a]phenanthrene-3,17-diol (**2p**):

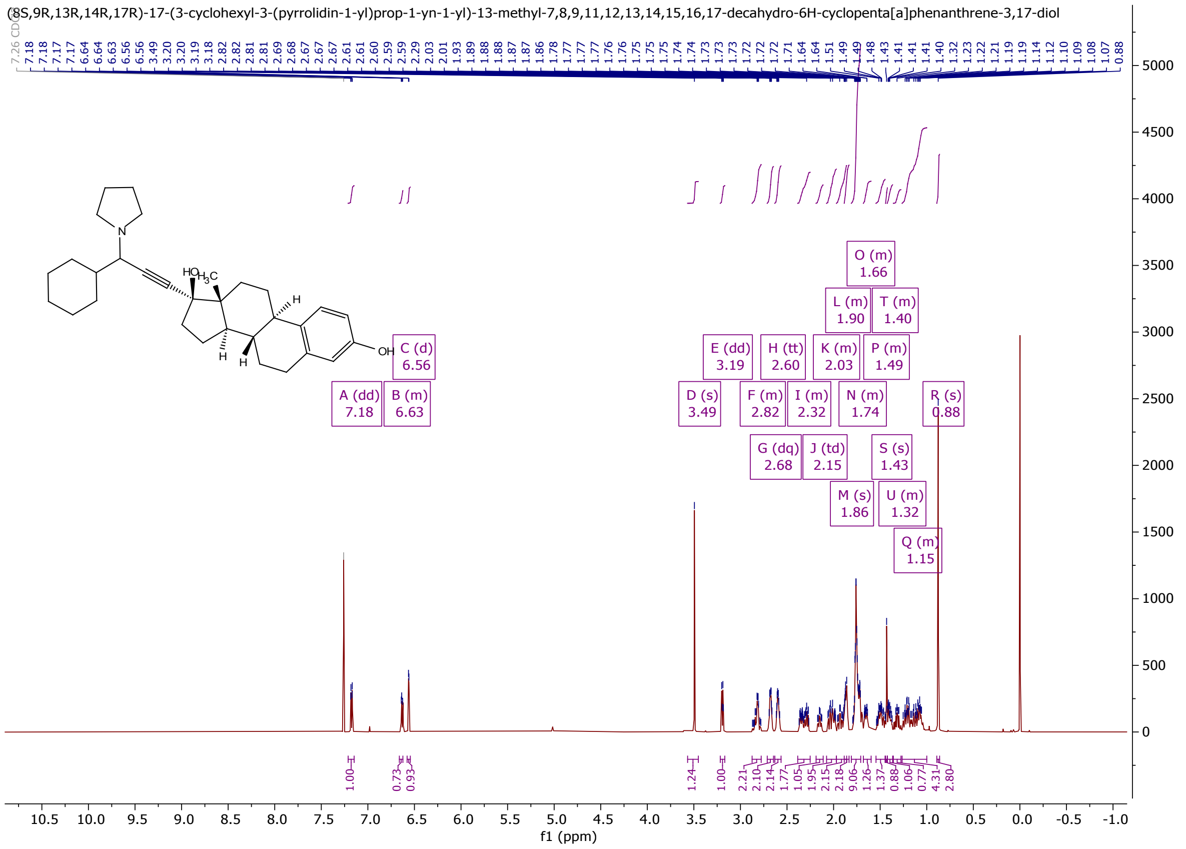

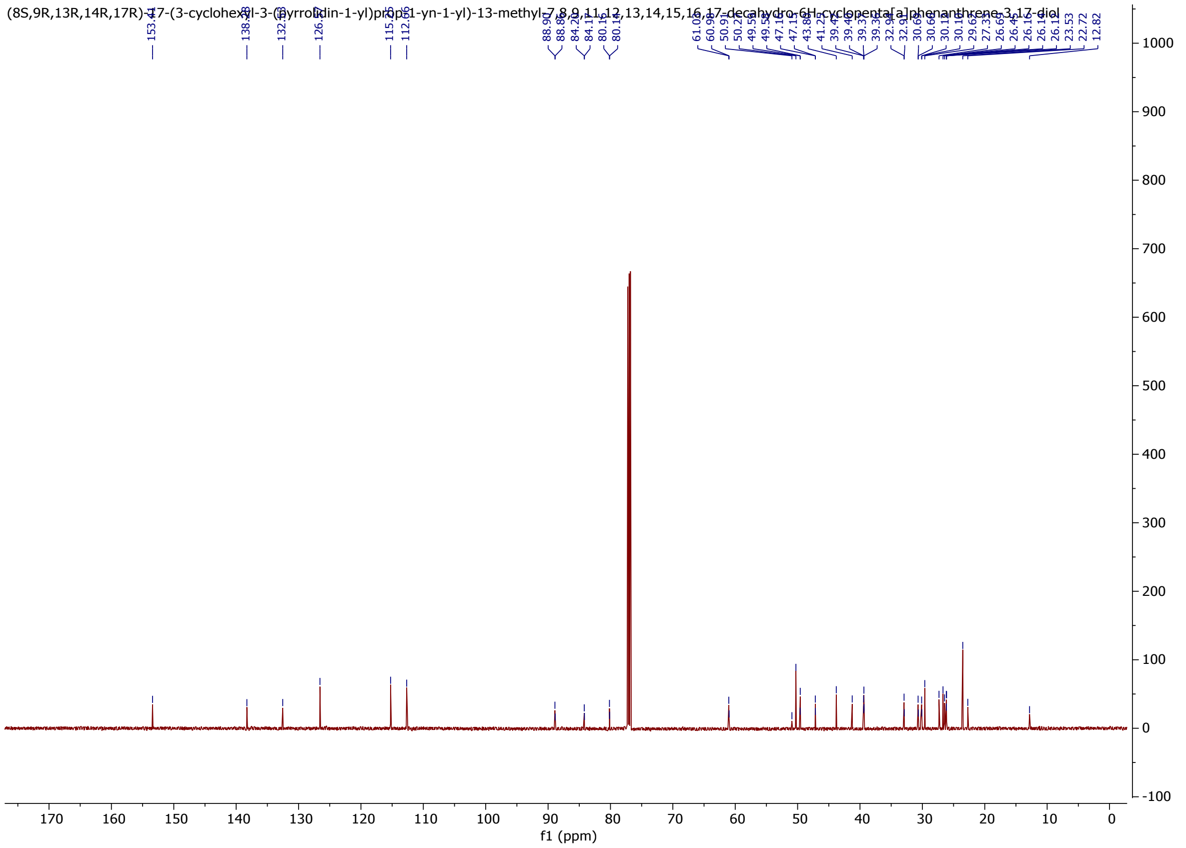

Figure S47: NMR spectra of (**2p**). Additional peaks correspond to a minor diastereomer

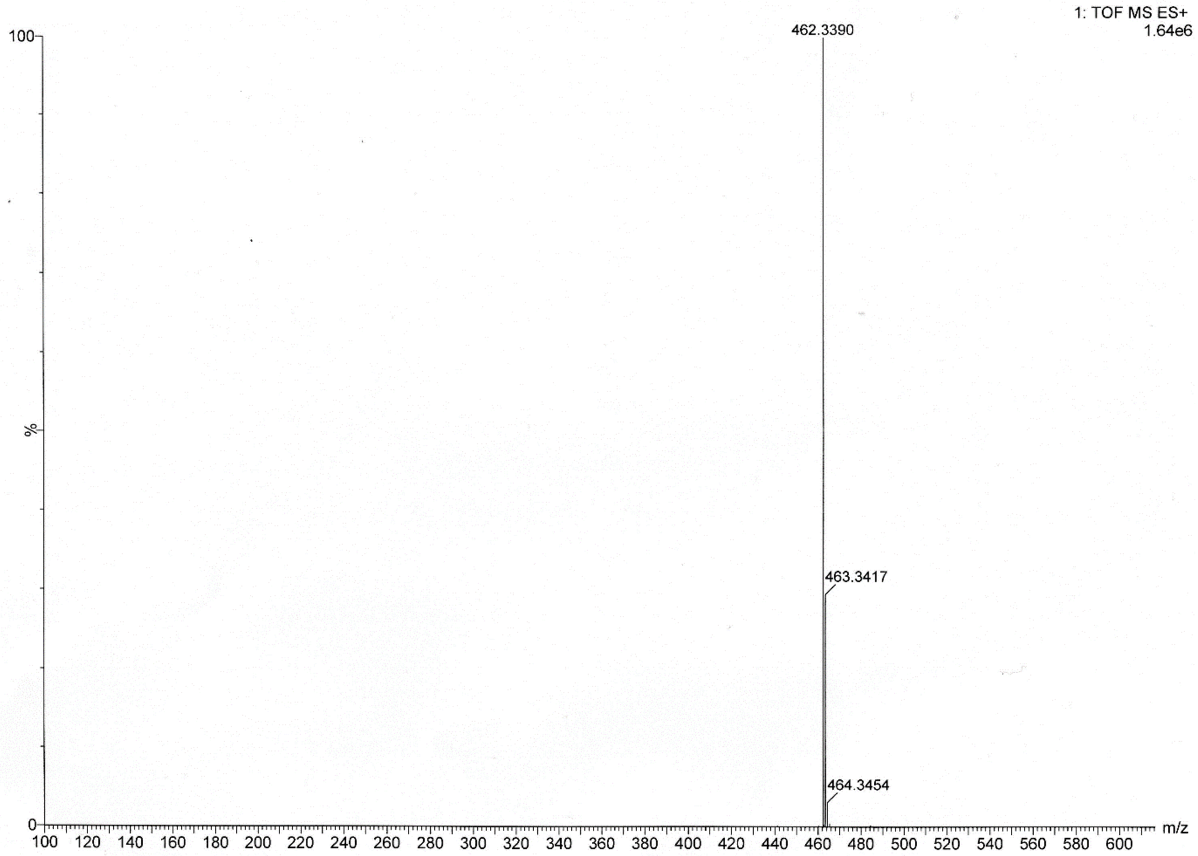

Figure S48: HRMS spectra of (**2p**)

2-(4-(4-Chlorophenyl)-2,3,9-trimethyl-6H-thieno[3,2-f][1,2,4]triazolo[4,3-a][1,4]diazepin-6-yl)-N-(4-cyclohexyl-4-(pyrrolidin-1-yl)but-2-yn-1-yl)acetamide

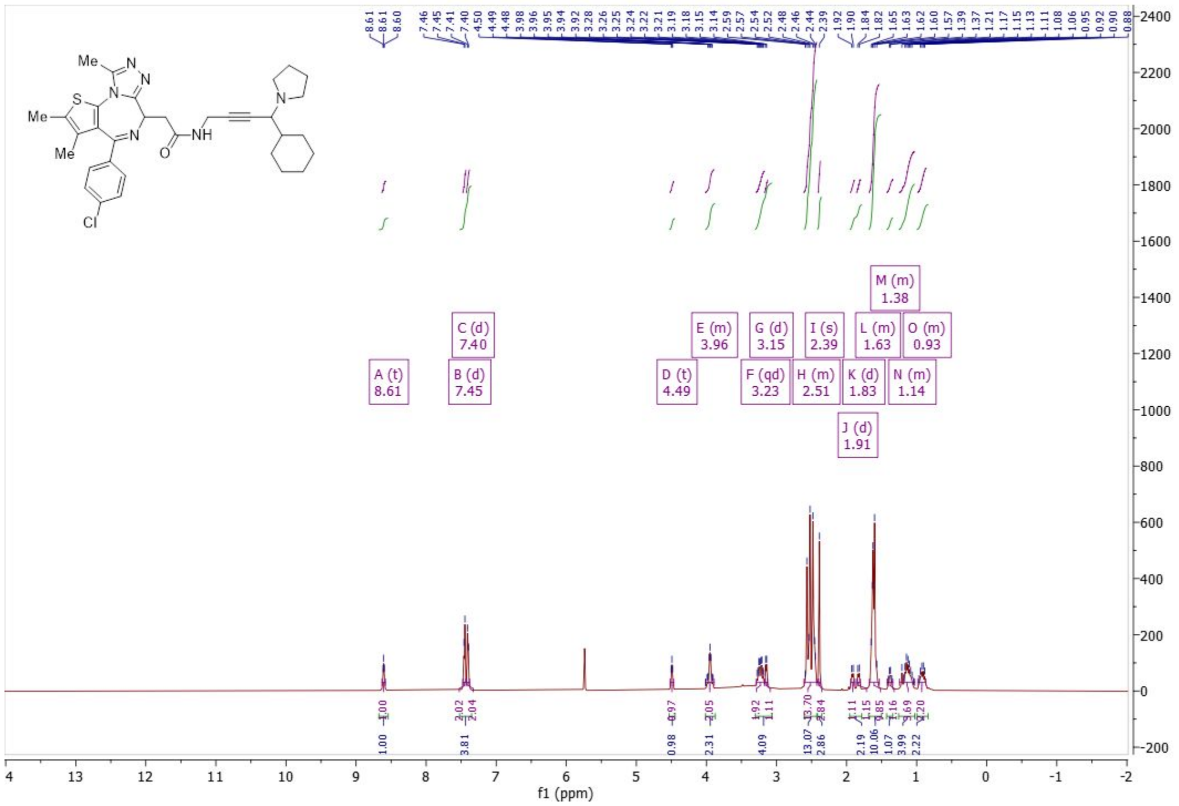

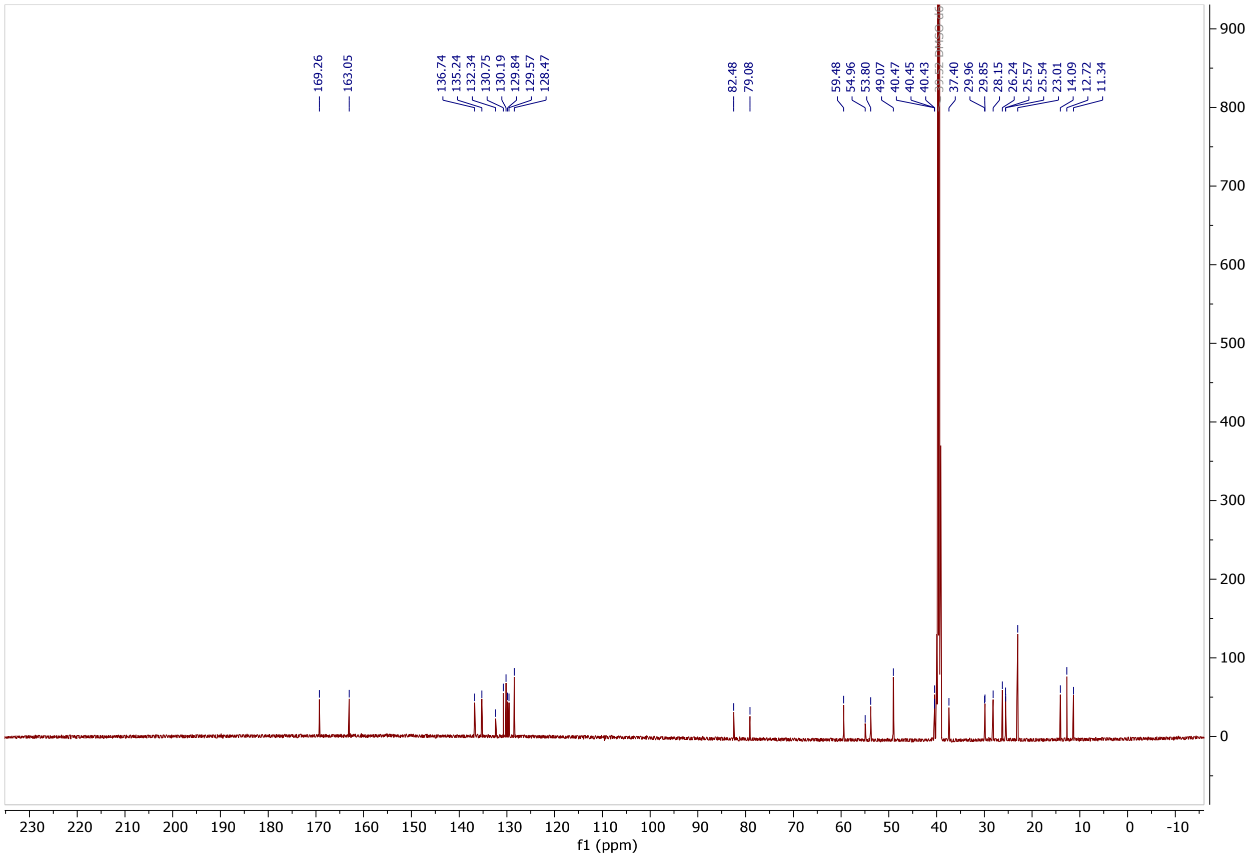

Figure S49: NMR spectra of (**2q**)

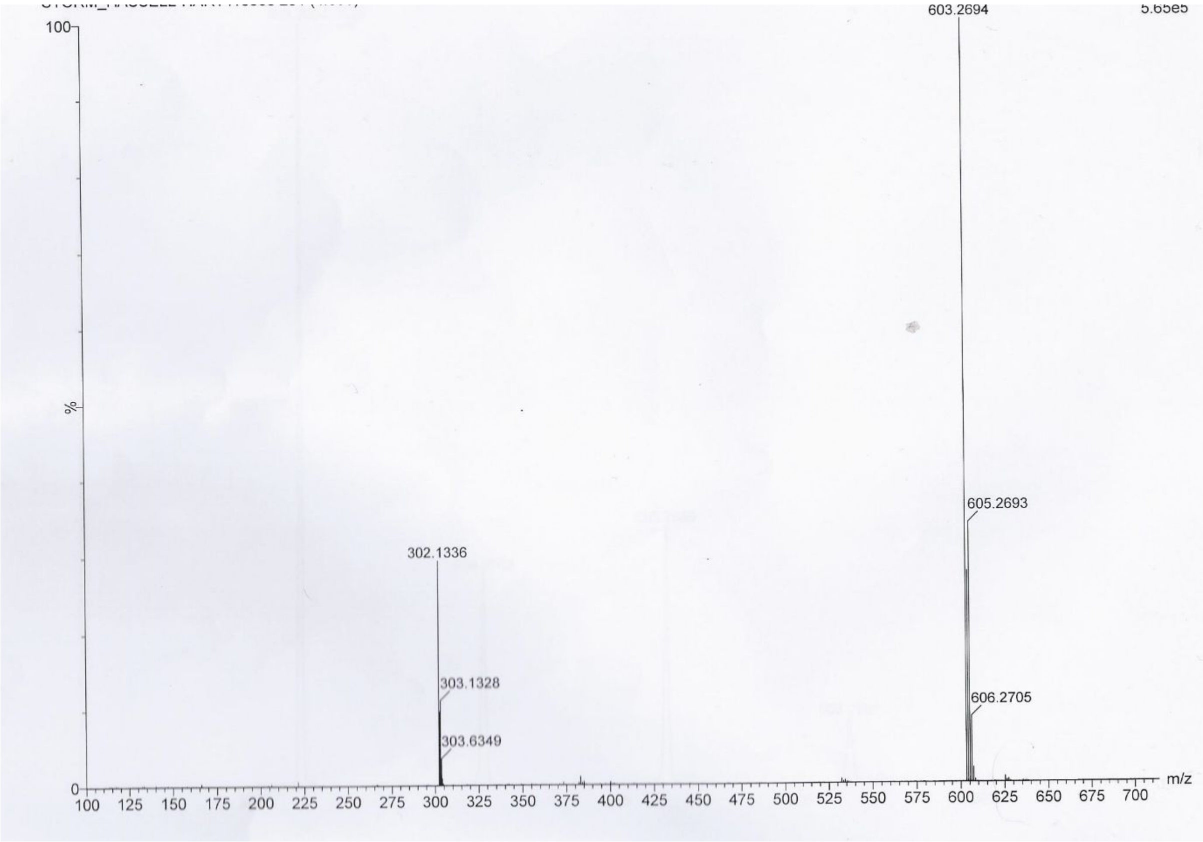

Figure S50: HRMS spectra of (**2q**)

N-(3-(3-Cyclohexyl-3-(pyrrolidin-1-yl)prop-1-yn-1-yl)phenyl)-6,7-bis(2-methoxyethoxy)quinazolin-4-amine

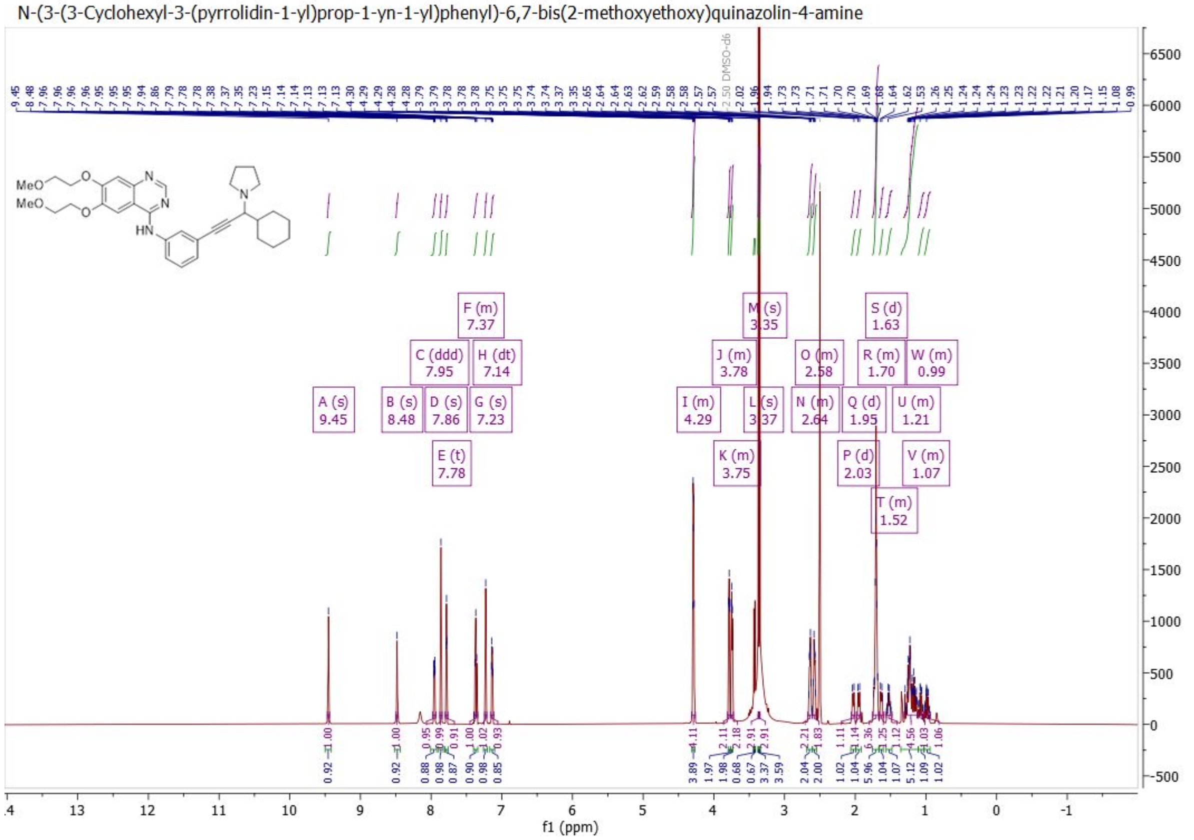

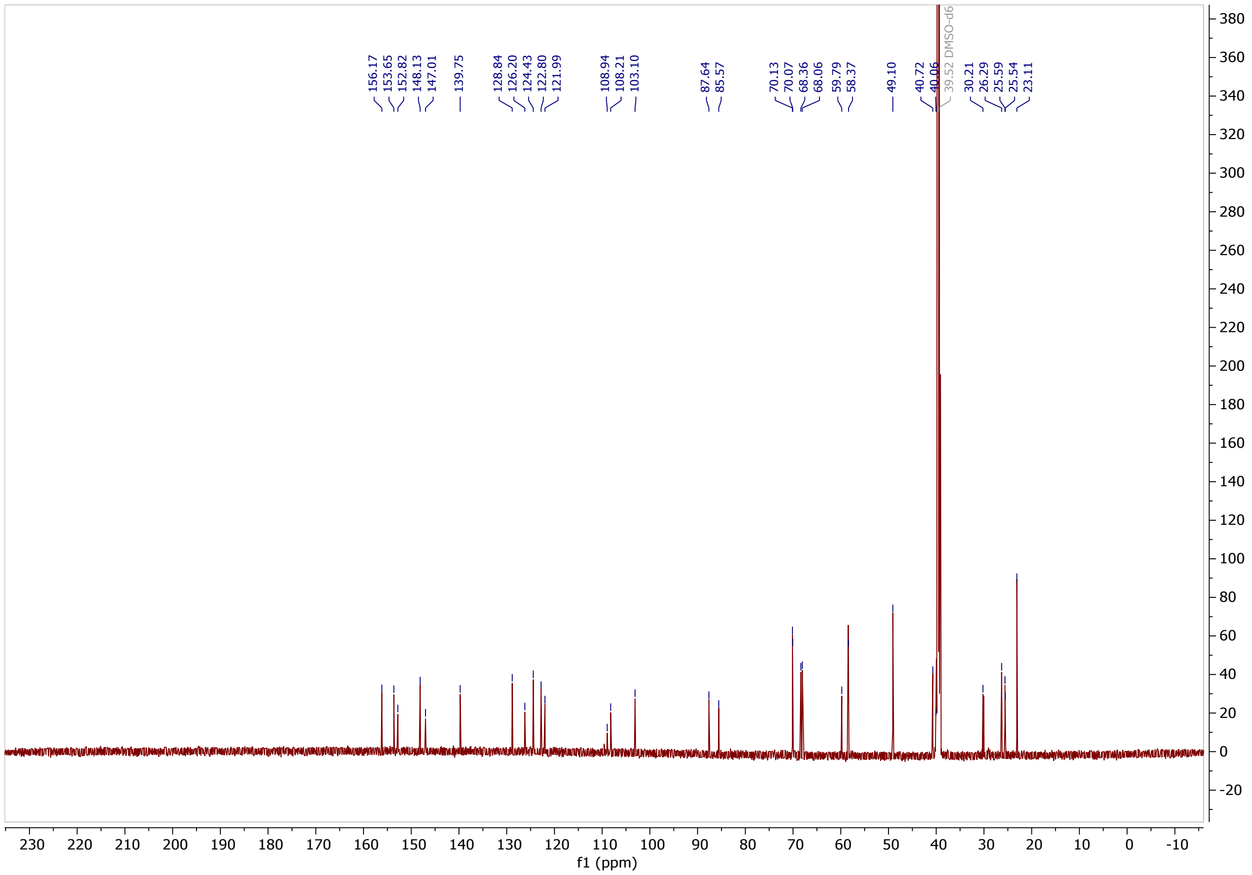

Figure S51: NMR spectra of (**2r**)

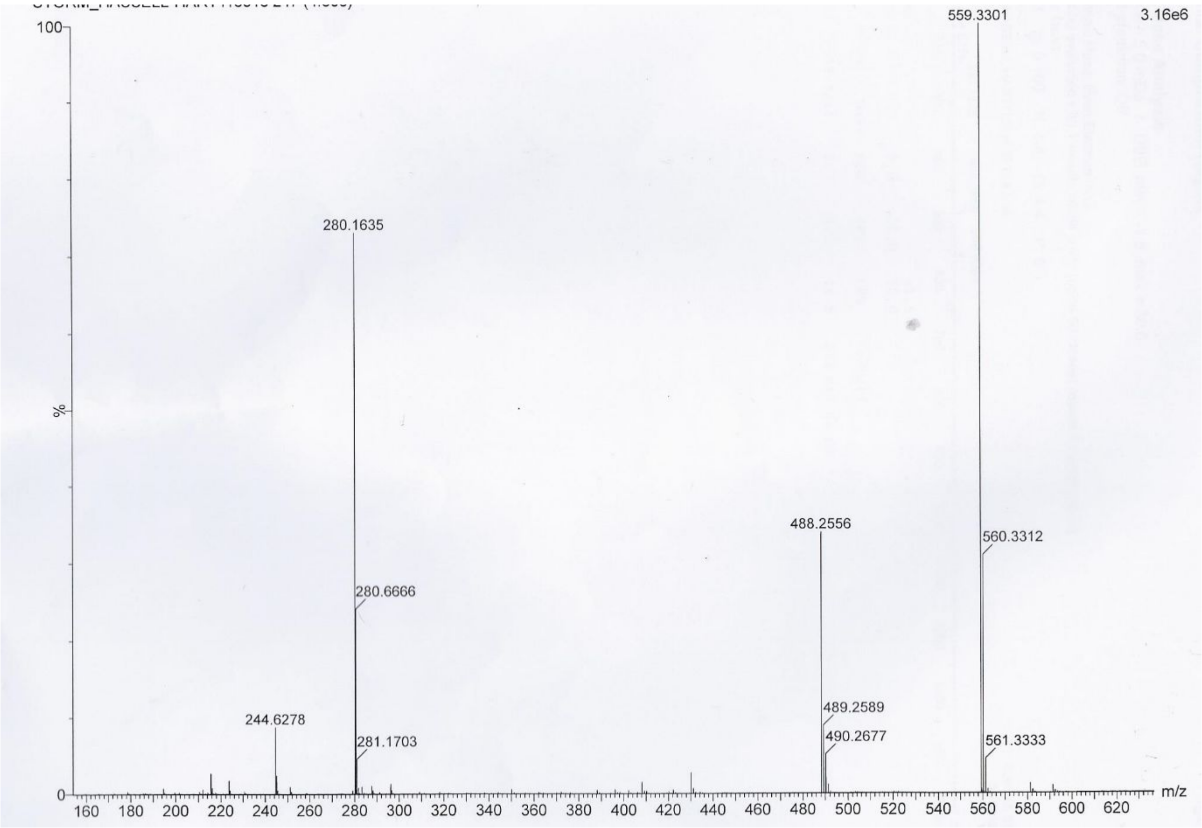

Figure S52: HRMS spectra of (**2r**)

References

(1) Stoll, S.; Schweiger, A. EasySpin, a Comprehensive Software Package for Spectral Simulation and Analysis in EPR. *J. Magn. Reson.* **2006**, *178* (1), 42–55. https://doi.org/10.1016/j.jmr.2005.08.013.

(2) Coles, S. J.; Gale, P. A. Changing and Challenging Times for Service Crystallography. *Chem. Sci.* **2012**, *3* (3), 683–689. https://doi.org/10.1039/c2sc00955b.

(3) Sheldrick, G. M. Crystal Structure Refinement with SHELXL. *Acta Crystallogr. Sect. C Struct. Chem.* **2015**, *71* (1), 3–8. https://doi.org/10.1107/S2053229614024218.

(4) Spek, A. L. Single-Crystal Structure Validation with the Program PLATON. *J. Appl. Crystallogr.* **2003**, *36*, 7–13. https://doi.org/10.1107/S0021889802022112.

(5) Dolomanov, O. V.; Bourhis, L. J.; Gildea, R. J.; Howard, J. A. K.; Puschmann, H. OLEX2 : A Complete Structure Solution, Refinement and Analysis Program. *J. Appl. Crystallogr.* **2009**, *42* (2), 339–341. https://doi.org/10.1107/S0021889808042726.

(6) Farrugia, L. J. WinGX and ORTEP for Windows : An Update. *J. Appl. Crystallogr.* **2012**, *45* (4), 849–854. https://doi.org/10.1107/S0021889812029111.

(7) Macrae, C. F.; Edgington, P. R.; McCabe, P.; Pidcock, E.; Shields, G. P.; Taylor, R.; Towler, M.; Van De Streek, J. Mercury: Visualization and Analysis of Crystal Structures. *J. Appl. Crystallogr.* **2006**, *39*, 453–457. https://doi.org/10.1107/S002188980600731X.

(8) M. Llunell, D. Casanova, J. Cirera, P. Alemany, S. A. SHAPE. *SHAPE version 2.0.* **2010**, *Barcelona*.

(9) Frisch, M. J.; Trucks, G. W.; Schlegel, H. B.; Scuseria, G. E.; Robb, M. A.; Cheeseman, J. R.; Scalmani, G.; Barone, V.; Mennucci, B.; Petersson, G. A.; Nakatsuji, H.; Caricato, M.; Li, X.; Hratchian, H. P.; Izmaylov, A. F.; Bloino, J.; Zheng, G.; Sonnenberg, J. L.; Had, M.; Fox, D. J. Gaussian 09, Revision B.01. Gaussian, Inc., Wallingford, CT. 2010.

(10) Handy, N. C.; Cohen, A. J. Left-Right Correlation Energy. *Mol. Phys.* **2001**, *99* (5), 403–412. https://doi.org/10.1080/00268970010018431.

(11) Hoe, W. M.; Cohen, A. J.; Handy, N. C. Assessment of a New Local Exchange Functional OPTX. *Chem. Phys. Lett.* **2001**, *341* (3–4), 319–328. https://doi.org/10.1016/S0009-2614(01)00581-4.

(12) Lee, C.; Yang, W.; Parr, R. G. Development of the Colle-Salvetti Correlation-Energy Formula into a Functional of the Electron Density. *Phys. Rev. B* **1988**, *37* (2), 785–789. https://doi.org/10.1103/PhysRevB.37.785.

(13) Weigend, F.; Ahlrichs, R. Balanced Basis Sets of Split Valence, Triple Zeta Valence and Quadruple Zeta Valence Quality for H to Rn: Design and Assessment of Accuracy. *Phys. Chem. Chem. Phys.* **2005**, *7* (18), 3297–3305. https://doi.org/10.1039/b508541a.

(14) Weigend, F. Accurate Coulomb-Fitting Basis Sets for H to Rn. *Phys. Chem. Chem. Phys.* **2006**, *8* (9), 1057–1065. https://doi.org/10.1039/b515623h.

(15) Grimme, S.; Antony, J.; Ehrlich, S.; Krieg, H. A Consistent and Accurate Ab Initio Parametrization of Density Functional Dispersion Correction (DFT-D) for the 94 Elements H-Pu. *J. Chem. Phys.* **2010**, *132* (15), 154104. https://doi.org/10.1063/1.3382344.

(16) Cossi, M.; Barone, V.; Cammi, R.; Tomasi, J. Ab Initio Study of Solvated Molecules: A New Implementation of the Polarizable Continuum Model. *Chem. Phys. Lett.* **1996**, *255* (4–6), 327–335. https://doi.org/10.1016/0009-2614(96)00349-1.

(17) Tomasi, J.; Mennucci, B.; Cammi, R. Quantum Mechanical Continuum Solvation Models. *Chemical Reviews*. American Chemical Society 2005, pp 2999–3093. https://doi.org/10.1021/cr9904009.

(18) Henkelman, G.; Arnaldsson, A.; Jónsson, H. A Fast and Robust Algorithm for Bader Decomposition of Charge Density. *Comput. Mater. Sci.* **2006**, *36* (3), 354–360.

https://doi.org/10.1016/j.commatsci.2005.04.010.
(19) Jmol: an open-source Java viewer for chemical structures in 3D, www.jmol.org. Date accessed 06.10.19
